# The SAMPL9 host–guest blind challenge: an overview of binding free energy predictive accuracy†

**DOI:** 10.1039/d3cp05111k

**Published:** 2024-03-04

**Authors:** Martin Amezcua, Jeffry Setiadi, David L. Mobley

**Affiliations:** a Department of Pharmaceutical Sciences, University of California, Irvine Irvine California 92697 USA dmobley@mobleylab.org; b Department of Chemistry, University of California, Irvine Irvine California 92697 USA; c Skaggs School of Pharmacy and Pharmaceutical Sciences, University of California, San Diego La Jolla California 92093 USA

## Abstract

We report the results of the SAMPL9 host–guest blind challenge for predicting binding free energies. The challenge focused on macrocycles from pillar[*n*]-arene and cyclodextrin host families, including WP6, and bCD and HbCD. A variety of methods were used by participants to submit binding free energy predictions. A machine learning approach based on molecular descriptors achieved the highest accuracy (RMSE of 2.04 kcal mol^−1^) among the ranked methods in the WP6 dataset. Interestingly, predictions for WP6 obtained *via* docking tended to outperform all methods (RMSE of 1.70 kcal mol^−1^), most of which are MD based and computationally more expensive. In general, methods applying force fields achieved better correlation with experiments for WP6 opposed to the machine learning and docking models. In the cyclodextrin-phenothiazine challenge, the ATM approach emerged as the top performing method with RMSE less than 1.86 kcal mol^−1^. Correlation metrics of ranked methods in this dataset were relatively poor compared to WP6. We also highlight several lessons learned to guide future work and help improve studies on the systems discussed. For example, WP6 may be present in other microstates other than its −12 state in the presence of certain guests. Machine learning approaches can be used to fine tune or help train force fields for certain chemistry (*i.e.* WP6-G4). Certain phenothiazines occupy distinct primary and secondary orientations, some of which were considered individually for accurate binding free energies. The accuracy of predictions from certain methods while starting from a single binding pose/orientation demonstrates the sensitivity of calculated binding free energies to the orientation, and in some cases the likely dominant orientation for the system. Computational and experimental results suggest that guest phenothiazine core traverses both the secondary and primary faces of the cyclodextrin hosts, a bulky cationic side chain will primarily occupy the primary face, and the phenothiazine core substituent resides at the larger secondary face.

## Abstract 2

We report the results of the SAMPL9 host–guest blind challenge for predicting binding free energies.

## Graphical abstract

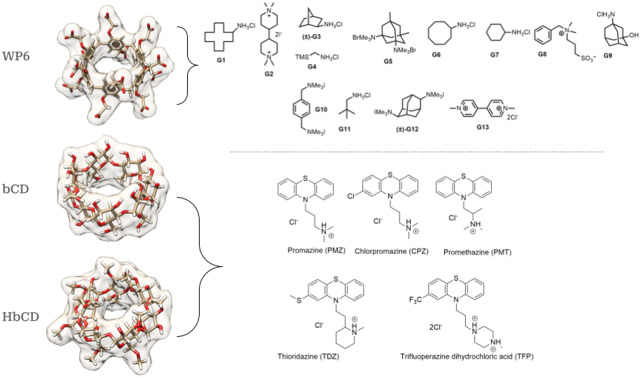

## 1 Introduction

The process of drug discovery has evolved from traditional iterations of trial-and-error synthesis of molecules and evaluation *via* assay to identify target binders. Even in modern drug discovery, getting a viable therapeutic or drug approved to help patients is costly both in terms of resource and time.^1^ Computational tools and software for computer-aided drug design (CADD) have seen an increase in application for early stage drug discovery campaigns to help circumvent these resource and time challenges.^2^ For example, quantitative simulation-based predictions of binding free energies (BFEs) or physical properties are more commonly used and have shown increased promise to help select candidate binders and accelerate lead optimization by prioritizing which compounds to synthesize.^3–8^ Although BFEs are computationally demanding, recent successes, advancement in methods, and the increase in computational resources and power, it is foreseen they will have an increasingly important role in drug discovery.^8,9^ For BFEs, such as the relative binding free energy (RBFE) or absolute binding free energy (ABFE), predictions must be accurate enough to be useful in guiding a drug discovery team's decisions. However, the accuracy of BFE calculations depends on several factors (some are described in ref. 10–19) including the accuracy of the chosen energy model and efficient sampling of the target (such as a protein) and ligand in the bound and unbound states.

There have been recent developments and extended applications of molecular dynamics (MD) to BFEs.^20^ These, coupled with continued increases in computing power, bring more excitement and promise for the role of BFEs in CADD. Common approaches for predicting binding affinity, particularly BFEs, rely on MD simulations and require the exploration of all relevant conformational spaces of a target and ligand of interest. Certain conformational dynamics of a biomolecule, such as a protein, could require simulations up to the millisecond timescales to capture such motions, and thus might not always be accessible. BFE simulations can also contain water, ions, and sometimes cofactors that make the system much larger and more challenging to achieve adequate sampling and convergence.^21^ In such cases, it would be difficult to assess whether the inaccuracy of a computational method is due to limitations in the chosen energy model, limited sampling, or other factors such as the protonation state of an ionizable residue. This can make BFE calculations for biomolecular systems less than ideal from the perspective of testing methods.

The SAMPL (statistical assessment of the modeling of proteins and ligands) blind challenges have been run since 2008 to test and improve computational methods, with the goal of innovating and advancing reliable predictive tools for drug design.^22–24^ SAMPL has served as a crowdsourcing platform for the computational community to test their methods on systems designed to focus on specific modeling challenges. Free energy techniques were among the most successful methods in the SAMPL challenges. As a result, SAMPL has raised awareness and understanding of some sources of error and understanding of important molecular recognition events such as the importance of adequate sampling of water rearrangements.^25^

Several iterations of SAMPL have had a host–guest component examining the binding of small molecule “guests” to supramolecular “hosts”, particularly macrocycles. Supramolecular chemistry has seen a substantial increase in popularity in recent years, particularly as hosts have industrial and medical applications through compound stabilization or enhancing solubility, drug delivery, and sequestration agents.^26–28^ In addition, since hosts are typically much smaller and more rigid than biomolecules, they make reasonable surrogates for proteins to help test and improve computational methods for BFEs. Recent SAMPL host–guest challenges have demonstrated that some methods can predict binding free energies within 1 kcal mol^−1^, but in some instances they continue to present some modeling difficulties.^11,29–31^ The lessons learned from the SAMPL host–guest challenges can push improvement and innovation of computational methods, and eventually with protein–ligand systems which is another phase of the SAMPL series of challenges.

### 1.1 SAMPL9 host–guest systems

The SAMPL9 host–guest blind challenge consisted of two datasets with each containing guests binding to one of two families of hosts, pillararene^32,33^ or a β-cyclodextrin (bCD)^34,35^ The hosts chosen from these families for the challenge were WP6, and two cyclodextrins – bCD and hexakis-2,6-dimethyl-β-cyclodextrin (H26DM-bCD or HbCD).

**Fig. 1:**
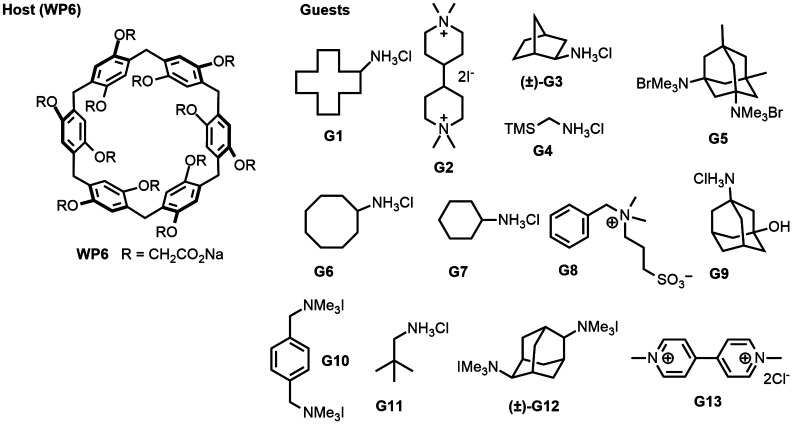
2D structures of host and guest series for the SAMPL9 WP6 challenge. Shown is the structure of the WP6 host with the carboxylic arms denoted with R groups (left).^40^ The guests were code named G1–G13 and were primarily hydrophobic and cationic/dicationic guests (right). Experimental binding measurements for guests G3 and G12 were obtained with racemic mixtures of both enantiomers (marked with a (±)).

In prior SAMPL challenges, the Isaacs group provided unpublished experimental binding thermodynamic values (free energy and enthalpy) of guests towards cucurbituril[*n*]-type receptors (*i.e.* CB[*n*] (*n* = 7, 8), acyclic CB[*n*] (trimer trip), and glycoluril-based molecular clips). Like the macrocyclic cucurbiturils, WP6 (a derivative of pillar[6]arene) is a macrocyclic molecular container used as a sequestration agent that has shown a high affinity for hydrophobic cationic guests^33,36^ and was sought after for SAMPL. Thus, for SAMPL9, the guest dataset consisted of thirteen hydrophobic cationic guests (Fig. 1) like, but not limited to, adamantanes and viologens.^36,37^ WP6 is composed of six phenylene groups containing CH_2_ linkers. Each phenylene group contains two anionic carboxylate arms, for a total of twelve arms (Fig. 1 and 3). This potentially gives WP6 twelve negatively charged groups at pH 7.4.

An issue that SAMPL host–guest challenge participants encountered in prior iterations was a poor conformational sampling of the host (particularly for CB[*n*]-type acyclic receptors). Using WP6, which is macrocyclic but has some degree of flexibility, can put focus on conformational sampling. The carboxylate arms of WP6 (OCH2CO2 groups) can all pass through the annulus (above or below the ring shape formed by the phenylene groups) to give a second host enantiomer.^38,39^ Thus, adequate sampling of these flexible regions may be necessary to achieve accurate predictions.

**Fig. 2:**
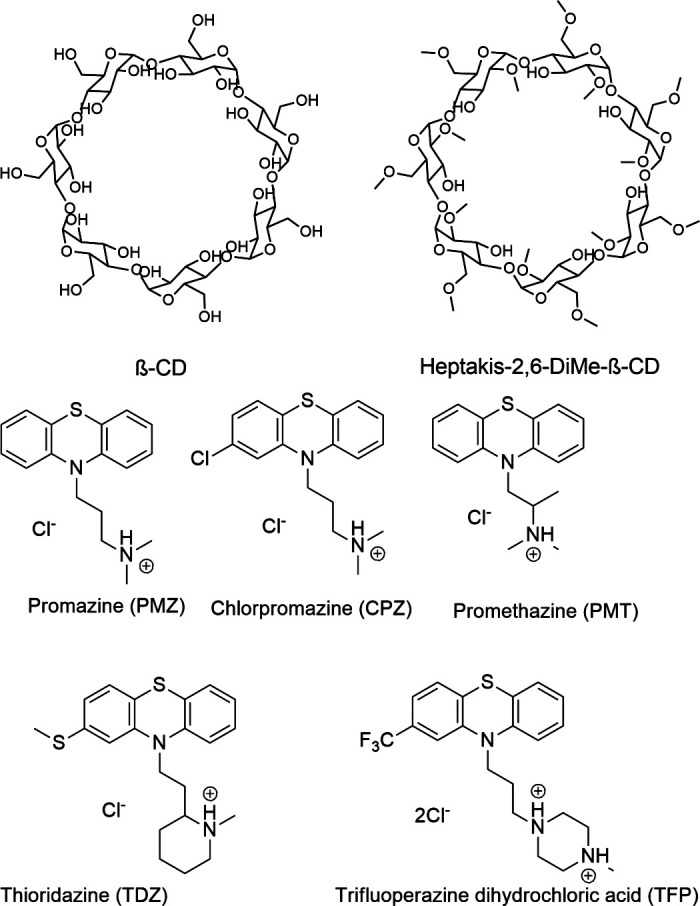
2D structures of the SAMPL9 bCD challenge hosts and guests series. (A) Shows the structure of the bCD and HbCD hosts. (B) The phenothiazine based guests (PMZ, CPZ, PMT, TDZ, and TFP) in the series.

**Fig. 3:**
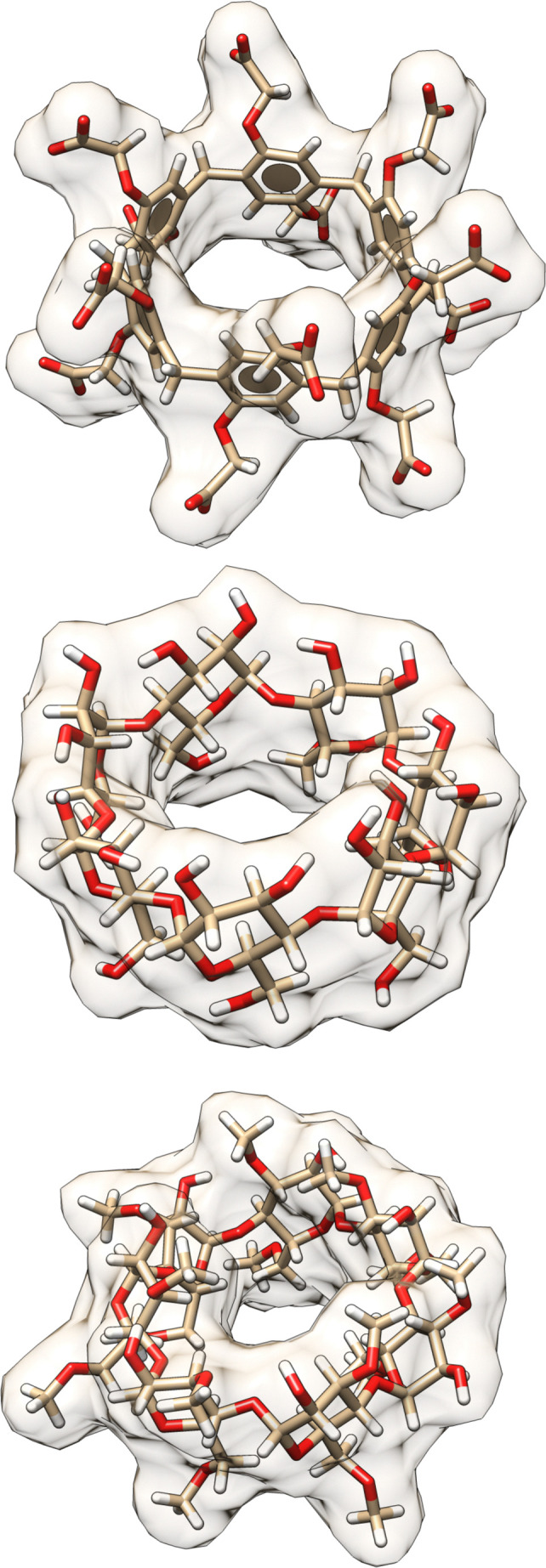
3D structures of hosts in the SAMPL9 host–guest challenge. Shown are the 3D structures of the WP6 (top), bCD (middle), and HbCD (bottom) hosts. Host atoms and bonds are represented in sticks, the shape of the host is depicted with a filled and transparent molecular surface representation showing each host cavity. Benzene rings are represented by aromatic discs (only present for WP6). Carbon, oxygen, and hydrogen atoms are shown in beige, red, and white colors, respectively.

Likewise, the Gilson group has provided experimental thermodynamic data on different cyclodextrins and cyclodextrin derivatives for SAMPL, previously in SAMPL7 and for this work. Cyclodextrins are of interest because of their application towards modification of molecular properties of target guests upon complexation and ultimately leading to their application in drug delivery.^41–44^ Cyclodextrins such as bCD and its derivative HbCD are macrocycles composed of seven α-d-(+)-glucopyranose units linked *via* 1,4-glycosidic bonds.^45^ In general, the center and cavity of the host are hydrophobic and the primary and secondary hydroxyl group ends are hydrophilic (Fig. 2). In the case of HbCD, there are primary and secondary methoxy groups at both ends of the donut-shaped host instead of hydroxyls (Fig. 2 and 3). The guests for this dataset are 5 phenothiazine based drugs: promazine (PMZ), chlorpromazine (CPZ), promethazine (PMT), thioridazine (TDZ), and trifluoperazine (TFP). (Fig. 2) Phenothiazines, particularly PMT, are widely prescribed in oral doses as an antihistamine and for sedation.^46^ The associated side effects of phenothiazines are described in the literature.^47,48^ bCD and HbCD can help stabilize and enhance the solubility of phenothiazines, and provide some controlled drug release to reduce toxic side effects.^48,49^

Computationally, these systems introduce some difficulties which can result in inaccurate BFE predictions for some methods and have been documented in the literature.^25,29,50,51^ Due to the asymmetric structure of bCD and HbCD hosts, a host–guest complex can exist with the guest in the primary, secondary, and/or perhaps a “surface” orientation.^30,35,52^ It is speculated that the surface orientation is not a true binding mode, but rather an unbinding event that may be necessary for a guest to flip orientation. Therefore, predicting the binding mode of these complexes would be part of the challenge, and it is not always trivial even for systems like these which are assumed to be simpler. As shown in SAMPL7, a specific functionalization of bCD designed to orient a guest in a particular orientation did not always lead to the guest occupying that specific orientation.^35^ Previous experimental studies of bCD complexation with PMT^48^ described a predicted binding mode with bCD, providing a starting point for this challenge. The other phenothiazines were expected to occupy a similar binding mode. In addition, some of the phenothiazines (CPZ, TDZ, TFP) have functionalization at the phenothiazine moiety, making the guest asymmetric. Thus, additional sub-orientations in each face may need to be considered or sampled.

## 2 Methods

In this section, we give the details on how the challenge is organized, briefly describe methods used by participants for their submissions, describe the details of reference calculations, summarize experimental details and methodologies (experimental studies are published separately^40,53^), and describe the statistical analysis approach and evaluate methods.

### 2.1 Challenge organization

The host–guest challenge of SAMPL9 was organized similar to SAMPL7-8, where the main part of the challenge (for ranking) allows participants to submit a single method as a ranked submission for any or all of the datasets (WP6 and bCD/HbCD). For the main analysis of the challenge, only ranked submissions were considered. Participants were encouraged to submit additional submissions, and any additional submissions were not included in the formal ranking. A requirement for a submission for either dataset, regardless if ranked or non-ranked, was to include all guests. Participants formally submitted blind predictions to the relevant challenge submission server prior to the deadline. In addition, MA conducted blind reference calculations which were submitted in the non-ranked category. These provide a point of comparison with a previous literature method.

SAMPL has encouraged participants to submit additional submissions as non-ranked, as this provides additional methods for cross-comparison and opportunities for benchmarking. Additionally, this can allow for sensitivity analysis between variants of a specific method and facilitate tuning or improvement of the method(s), force field, and/or protocol, while also highlighting successes and limitations. For these reasons, the reference calculations conducted for this challenge include several variants of the method utilized, varying a single parameter in the approach (*i.e.* force field, restraints used, guest orientation, *etc.*) to probe the impact on predictive accuracy.

Host and guest structure files in the forms of MOL2, PDB, and SDF, as well as SMILES strings of guests, were prepared and published on the GitHub repository at the official start of each challenge. Where applicable, host and guest structures were provided with reasonable protonation states. However, some protonation states were questionable, thus participants were advised to exercise caution, particularly with the WP6 host and guests in the bCD/HbCD datasets.

A submission template file was made available for participants to fill in with all needed information and instructions. We required participants to follow the submission templates since we would use automated scripts to parse and run statistical analyses. Each submission required the following: predictions, participant name(s), participant organization(s), name of their method, list of software used, detailed method description, method category, and a ranked or non-ranked classification. In the predictions section, we provided the code names for the host–guest systems of the challenge and required the predicted free-energy, free-energy SEM, and free energy model uncertainty in units of kcal mol^−1^. The predicted binding enthalpy, binding enthalpy SEM, and binding enthalpy model uncertainty were optional. All files, data, and instructions are made available in the SAMPL9 GitHub repository (https://github.com/samplchallenges/SAMPL9/tree/main/host_guest/) under the relevant challenge dataset (WP6 or bCD (bCD and HbCD)). All submission files are available in the SAMPL9 GitHub repository (https://github.com/samplchallenges/SAMPL9/tree/main/host_guest/Analysis/Submissions/) in the relevant host directory (WP6 or bCD (includes bCD and HbCD)).

SAMPL9 data collection concluded around August 20, 2021, for the WP6 challenge, and November 11, 2021, for the cyclodextrins and phenothiazines challenge. The submission deadlines were set for November 15, 2021 (pushed back from a deadline of October 30, 2021) and February 23, 2022, respectively. The WP6-G4 guest was made optional (updated around November 8, 2021) since it contained silicon and may introduce modeling difficulties with some software. All challenge deadlines were posted in the relevant SAMPL9 host–guest challenge repository.

### 2.2 Statistical analysis of submissions

Accuracy or error statistics such as RMSE (root-mean-squared error), ME (mean error), and MAE (mean absolute error) provide a measure of how well a computational method agrees with experimental results. Correlation statistics such as *R*^2^ (coefficient of determination) and *τ* (Kendall Tau correlation coefficient) provide information on how well a computational method ordered guests and ranked them from best to worst, relative to the experiment. The SAMPL9 host–guest challenge statistical analysis was carried out using Python scripts adapted from previous SAMPL iterations, and we used the same error and correlation metrics^11,17,31^ that include RMSE, *R*^2^, *m* (linear regression slope), *τ*, ME, and MAE. The uncertainty in the metrics was determined by using bootstrapping with replacement as described in the literature.^17,57^ To identify and compare the predictive accuracy of methods towards individual host–guest systems, the RMSE, MAE, and ME of each were computed while considering all methods.

Submissions were separated into two main categories, ranked and non-ranked, and statistical analysis was performed for each category. We note that the non-ranked category included all submissions, and methods denoted as ranked or non-ranked. The ranked category analysis, which was for the formal competition, only considered methods denoted as ranked. All data, analysis plots and tables for ranked and non-ranked categories were made available in the relevant SAMPL9 GitHub repository as “Ranked Accuracy” or “All Accuracy” (https://github.com/samplchallenges/SAMPL9/tree/main/host_guest/Analysis).

### 2.3 Participant methodologies

In SAMPL9, methods used to compute BFEs varied and included both alchemical and physical approaches with general small molecule fixed charged force fields (OpenFF1.2.0 or OpenFF2.0.0 (https://openforcefield.org/force-fields/force-fields/), GAFF or GAFF2 (Generalized Amber force field), CGenFF (CHARMM generalized Force Field), and GROMOS (GROningen MOlecular Simulation)), explicit water models (TIP3P (Transferable Interaction Potential three-point), TIP4P-EW (transferable interaction potential four-point Ewald), SPC (simple point charge), or OPC3 (3-point optimal point charge)) and with AM1-BCC (Austin Model 1 Bond Charge Correction) or RESP (Restrained ElectroStatic Potential) charging schemes. In one case, the CGenFF atom parameterization was carried out *via* the ParamChem server.^58^ For this challenge, there were some methods that deployed newer approaches. For example, a method used a newly developed ABCG2 (AM1-BCC-GAFF2) method.^59^ Another method (Alchemical Transfer Method (ATM)^60–62^) utilized an in house development at Roivant Discovery, a force field parameter engine termed here as FFEngine. Although fixed charge force fields were common, one approach used a standard double decoupling method (DDM) with the polarizable atomic multiplole AMOEBA force field.^63–65^ One group submitted several predictions using an expanded ensemble (EE) method with OpenFF2.0.0. Briefly, EE methods involve a single simulation where the EE algorithm allows the adaptive sampling of the thermodynamic ensemble, sampling across alchemical interaction potentials.^14,66,67^

Other methods used for predicting BFEs in SAMPL9 included non-equilibrium and end-point approaches. Briefly, in the non-equilibrium unidirectional alchemical approach known as the virtual Double System Single Box (vDSSB) method, a guest is annihilated while bound in the host cavity, and the unbound guest is then grown in bulk solvent and kept far away from the host *via* restraints.^54,68^ Multiple participants used end-point MM/PBSA (molecular mechanics Poisson–Boltzmann surface area) and MM/GBSA (generalized born surface area) methods, while one participant included ELIE (extended linear interaction energy) fitting to their end-point approach. The ELIE approach was inspired by both LIE (linear interaction energy) and MM/PBSA methods, where scaling coefficients obtained from fitting calculated BFEs to measured values of guests in a training set are applied to the energy terms in the MM/PBSA free energy equation to estimate BFEs.^59,69^ Note that this approach used ABCG2 mentioned in the previous paragraph. Another set of predictions submitted in this challenge was with a method based on docking, using the smina code for docking (https://sourceforge.net/projects/smina/) and the vinardo scoring function for scoring. A different method utilized available and published binding measurements of complexes to perform machine-learning based predictions. For a more detailed description of each method, please see the submission text files in the SAMPL9 GitHub repository (https://github.com/samplchallenges/SAMPL9/tree/main/host_guest/Analysis/Submissions/) or see Table 1 for available literature.

**Table 1:** Summary of methods (ranked and non-ranked) used in the SAMPL9 host–guest challenge for binding free energy calculations. Explicit and/or implicit solvents are flagged by an (E) or (I) respectively. If a correction approach was taken by a method, it is flagged with a (C). If the host (H) and guest (G) are parameterized with a different energy model, they will be flagged respectively. If a machine learning or docking approach was taken, certain categories are not relevant and flagged with an asterisk (*). Reference calculations are flagged with a double asterisk (**)

ID	sid	Energy model	Solvent model	Sampling	Ranked	SAMPL8 references
**WP6**						
DOCKING/SMINA/VINARDO	1	Vina	—*	—*	No	
vDSSB/GAFF2/OPC3/HREM (C)	2	GAFF2-AM1BCC	OPC3 (E)	HREM	Yes	54
MACHINE-LEARNING/NNET/DRAGON-descriptors	3	xTB-GFN2B	—*	—*	Yes	
EE/Openff-2.0/TIP3P/MD-EE/WL_RL.02_L.01	4	OFF2.0-AM1BCC	TIP3P (E)	MD	Yes	55
EE/Openff-2.0/TIP3P/MD-EE/All-data	5	OFF2.0-AM1BCC	TIP3P (E)	MD	No	55
EE/Openff-2.0/TIP3P/MD-EE/RL_8_only	6	OFF2.0-AM1BCC	TIP3P (E)	MD	No	55
DDM/AMOEBA/BAR	7	AMOEBA	AMOEBA (E)	MD	Yes	
ELIE/GAFF2-ABCG2/TIP3P/MD/MMPBSA	8	GAFF2-AM1BCC	TIP3P (E)	MD	Yes	
EE/Openff-2.0/TIP3P/MD-EE/WL_RL.02_L.01/corrected (C)	17	OFF2.0-AM1BCC	TIP3P (E)	MD	No	55
APR/GAFF2/TIP3P/MD-US/MBAR**	18	GAFF2-AM1BCC	TIP3P (E)	MD-US	No	
APR/OFF1.2.0/TIP3P/MD-US/MBAR**	19	OFF1.2.0-AM1BCC	TIP3P (E)	MD-US	No	
APR/OFF2.0.0/TIP3P/MD-US/MBAR**	20	OFF2.0.0-AM1BCC	TIP3P (E)	MD-US	No	

**CD-bCD and HbCD**						
PMF/GAFF-RESP/TIP4PEW/SMD	10	GAFF2-RESP	TIP4PEW (E)	MD	Yes	
DD/GROMOS-53A6_glyc/SPC/MD	11	GROMOS-53A6	SPC (E)	MD	Yes	
MD/GAFF-RESP/TIP4PEW/MM-GBSA	12	GAFF2-RESP	TIP4PEW (E/I)	MD	No	
MD/GAFF-RESP/TIP4PEW/MM-PBSA	13	GAFF2-RESP	TIP4PEW (E/I)	MD	No	
ATM/FFENGINE/TIP3P/HREM	14	FFEngine-GFN2-xTB/BCC	TIP3P (E)	HREM	Yes	56
MD/GAFF-RESP/TIP4PEW/MM-GBSA_2	15	GAFF2-RESP	TIP4PEW (E/I)	MD	No	
DDM/FEP/MBAR/ParamChem	16	CGenFF	TIP3P (E)	MD	Yes	
APR/GAFF2/TIP3P/MD-US/MBAR**	21	GAFF2-AM1BCC	TIP3P (E)	MD-US	No	
APR/OFF1.2.0/TIP3P/MD-US/MBAR**	22	OFF1.2.0-AM1BCC	TIP3P (E)	MD-US	No	
APR/OFF2.0.0/TIP3P/MD-US/MBAR**	23	OFF2.0.0-AM1BCC	TIP3P (E)	MD-US	No	

### 2.4 Reference calculations

Reference calculations using the Attach-Pull-Release (APR) method^70,71^ were set up and performed by M. A. and J. S. using the pAPRika 1.0.4 toolkit (https://github.com/GilsonLabUCSD/pAPRika/tree/v1.0.4) and OpenMM 7.4.2^72^ as a simulation engine. Overall, simulations were run using 15 windows for the attach and release phases, and up to 46 umbrella sampling windows during the pull phase. The pull phase umbrella windows were spaced at 0.4 Å intervals, starting at 6 Å from the non-interacting anchor particles (called dummy atoms) and extending to a maximum distance of 24 Å. Production runs of each window in all phases (attach, pull, and release) were run up to 50 ns.

The starting structures were obtained in a manner similar to that used for the SAMPL8 reference calculations,^31^ by docking guests to the host using OEDock with the Chemgauss4 scoring function from the OpenEye Toolkits (release 2019.10.2).^73^ To compare general force fields, the bonded and initial Lennard-Jones parameters were obtained from GAFF2 as implemented in Antechamber,^74^ or assigned based on OpenFF v1.2.0^75^ or v2.0.0^76^ using openff-toolkit release v0.10.1.^77^ Each host-guest system was solvated with 2500 TIP3P water molecules in a rectangular box whose dimensions were approximately 40 × 40 × 63 cubic A. Sodium or chloride counterions with parameters from Joung and Cheatham^78^ were added as needed to neutralize each host–guest system, and additional sodium and chloride ions were added to match the experimental ionic strength. AM1BCC partial atomic charges for hosts and guests were generated using oequacpac (with function oequacpac.OEAssignCharges(mol, oequacpac.OEAM1BCCCharges( ))) as implemented with OpenEye Toolkits.

The attach-pull-release windows were prepared using pAPRika 1.0.4 and consisted of adding three dummy atoms (non-interacting anchor particles), selecting host and guest anchor atoms, configuring orientational restraints^79^ (also called Boresch-style restraints), adding solvent and ions, and preparing OpenMM XML files. Briefly, three dummy atoms are added to each system to help define and impose different restraints, required for APR BFEs, that control the translation and orientation of the host and guest relative to the dummy atoms. The dummy atoms are not used in the context of defining other optional restraints discussed below. In general, host and guest anchor atoms were defined by selecting three heavy atoms for each host (H1, H2, and H3) and two for each guest (L1 and L2), as shown in the schematic Fig. S5 (ESI†). For specific atom selection see Table S1 (ESI†). L1 was then shifted to the axis origin, and the vector of L1 and L2 was aligned to the *z*-axis to orient the host–guest complex. Three dummy atoms (D1, D2, and D3) were added to the host–guest complex below the guest and along the *z*-axis (Fig. S5, ESI†). The distances for D1, D2, and D3 from the guest were 6, 9, and 11.2 Å, respectively, while D3 was also offset by 2.2 Å along the *y*-axis.

Boresch-style restraints, as described in ref. 71 and 80, were used to control the translation and orientation of the host molecule while providing a frame of reference. After the anchor atoms are defined, one distance, one angle, and one dihedral restraints are imposed to control the hosts' translational degrees of freedom *via* D1–H1, D2–D1–H1, and D3–D2–D1–H1, respectively. The hosts' orientational degrees of freedom were controlled by one angle and two dihedral restraints *via* D1–H1–H2, D2–D1–H1–H2, and D1–H1–H2–H3, respectively (Fig. S5–S8, ESI†). Together, these restraints were also called “static” restraints since they are constant throughout the entire APR simulations. Static restraints do not alter the internal coordinates of the host; therefore, the static restraints free energy was not included since it does not contribute to the binding free energy.

In addition, restraints were also applied to the guest molecules during the attach phase. Two restraints were used to control the guest translation, *r* and *θ*, and one restraint to control its orientation *β* (Fig. S5–S8, ESI†). Only the polar angle of the guest orientation was restrained. For these restraints, the restraint free energies were obtained by scaling force constants from *λ* 0 to 1 over 15 windows. The free energy of releasing the restraints on the guest in the unbound state was calculated semi-analytically and included a standard-state correction at 1 M. The force constants applied to host static and guest orientational restraints are shown in Table S2 (ESI†).

To create the umbrella windows of the pull phase, the guest molecule was pulled by the host along the reaction coordinate (*r*) by increasing the distance between D1–L1 (Fig. S5, S6 and S8, ESI†). The guest molecules were pulled in intervals of 0.4 Å and up to a distance of 18 Å, for a total of up to 46 windows. Throughout the pull phase, the two angles *θ* and *β* (D2–D1–L1 and D1–L1–L2) were restrained at 180° (Fig. S5, S6 and S8, ESI†).

The restraints described up to this point are required for APR calculations. However, there are also optional restraints which can be set. One optional type of restraints are flat-bottom potential restraints applied to keep the guest in the host binding pocket during the attach phase and help define the bound state. These set of restraints are referred to as “wall” restraints and help create a boundary(ies) at the binding site. Wall restraints are intended to help improve the calculations through quicker convergence, particularly for weak binders by preventing dissociation of the host–guest complex, and were only turned on if the guest leaves the host binding site past a threshold (Fig. S7 and S9, ESI†) during the attach phase, thus pulling the guest back towards the host binding site. In addition, when these restraints are applied they do not contribute to the final binding free energy.^71^ For WP6, twelve wall restraints were set on the guests (*via* the L1 anchor atom) relative to the host carboxylate arms to keep the guest in the host binding cavity (defined by the ether oxygen atoms O1, O31, O25, O19, 013, O7, O2, O32, O26, O20, O14, and O8), as shown in Fig. S7 (ESI†). For the bCD and HbCD hosts, 7 wall restraints were set on the guests (*via* the L1 anchor atom) relative to the oxygen atoms (O3, O33, O28, O23, O18, O13, and O8) of each glucopyranoside linker, as shown in Fig. S9 (ESI†). The force constant (“*k* wall”) and distance threshold (“*r* wall”) used for the wall restraints are shown in Table S2 (ESI†).

Another set of optional restraints for APR calculations is conformational restraints that can be applied to either or both host and guest to facilitate sampling during simulations of the pull phase.^18,71^ These types of restraints are also referred to as “jack” or dihedral restraints. Dihedral restraints were turned on in the attach phase (over 15 windows) by scaling the force constants using *λ* scaling coefficients from 0 to 1, as described earlier for guest restraints. The free energy contributions of applying the dihedral restraints on a host molecule were calculated. In the release phase, dihedral restraints were turned off over 15 windows by scaling the force constants from 1 to 0. The free energy cost of releasing the dihedral restraints in the unbound state was calculated explicitly.

For reference calculations, jack or dihedral restraints were not applied to WP6 or its relevant guests. bCD is a flexible macrocycle, and when certain guests are bound, bCD can get distorted and trapped in a conformational sub-state during a simulation that can make the calculation difficult to convergence or converge to an incorrect estimate. Imposing two dihedral restraints on the glucopyranoside linkers was shown to maintain the bCD shape and circumvent this issue. Thus, the same set of dihedral restraints was applied to bCD and HbCD on the glucopyranoside linkers based on previous work.^71^ A pair of dihedrals for each glucopyranoside linker (Fig. S10, ESI†) were selected (of 108.7° and −112.5°) resulting in a total of 14 dihedral restraints. Dihedrals of 108.7° were defined by atoms C4, O3, C37, O34, C40, O33, C31, O29, C34, O28, C25, O24, C28, O23, C19, O19, C22, O18, C13, O14, C16, O13, C7, O9, and C10, O8, C1, O4. The dihedrals of −112.5° were defined by atoms C5, C4, O3, C37, C41, C40, O33, C31, C35, C34, O28, C25, C29, C28, O23, C19, C23, C22, O18, C13, C17, C16, O13, C7, and C11, C10, O8, C1. The parameters used for the dihedral restraints are shown in Table S2 (ESI†).

The simulations were run using a Langevin thermostat^81^ at a constant temperature of 298.15 K and a collision frequency of 1.0 ps^−1^, using a Monte Carlo barostat^82^ with a constant pressure of 1 atm. Host–guest systems were minimized up to a maximum of 5000 steps and equilibrated in the NPT ensemble for 1 ns. Production simulations were also run in the NPT ensemble for up to 50 ns per window. The non-bonded interaction was truncated with a 9.0 Å cutoff. The long-range electrostatic interactions were handled with the particle mesh Ewald (PME) method,^83,84^ and the long-range van der Waals interactions were treated with an isotropic dispersion correction.^85–87^ Simulation time steps were set to 4 fs with hydrogen mass repartitioning (HMR).^71,88,89^ The binding free energy quantities were estimated with thermodynamic integration (TI) and/or the multistate Bennett acceptance ratio (MBAR)^90^ method. Uncertainties for the TI estimates were calculated using block analysis as described in the literature.^71^ However, for reference submissions only the estimates with MBAR were included.

For WP6, guests G1, G3, G5–G7, G9, and G11 had titratable nitrogens with p*K*_a_ values of 10.45, 10.48, 10.34, 10.45, 10.45, 10.34, and 9.93 (as determined *via* ChemAxxon Chemicalize), respectively. The population states for the protonated variants at the experimental pH were all above 99%, thus reference binding free energies were obtained from protonated guests. Reference binding free energy calculations for bCD systems were obtained using the protonated (charged) state of the guests. Guest G8 was modeled as a zwitterion, with its titratable nitrogen in the protonated state (charged) and its sulfonate group deprotonated (charged).

**Fig. 4:**
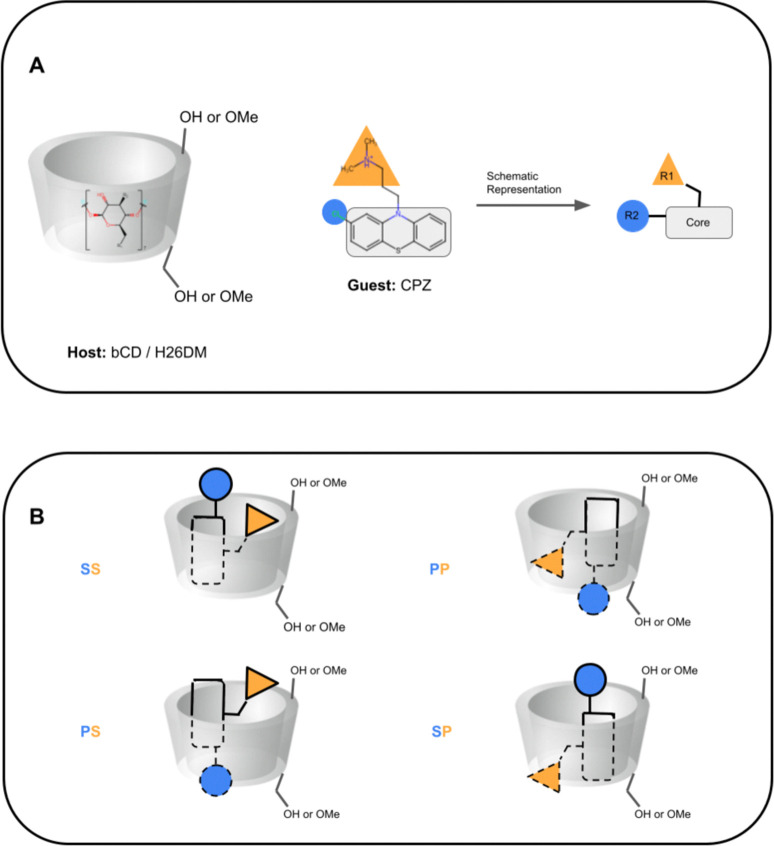
Schematic representation of cyclodextrin hosts and guests' primary and secondary orientation binding modes. (A) A schematic representation of cyclodextrin hosts and their general shape, and of phenothiazine guests. The primary face (smaller opening) of the host is at the bottom towards the primary alcohol (or methoxy) groups, and the secondary face (larger opening) is at the top of the host towards the secondary alcohol. The guest CPZ is used as an example, where the phenothiazine core is shown with a gray rectangle, the protonated amine tail is shown with a yellow triangle (R1), and phenothiazine functionalization is shown with a blue circle (R2). (B) Schematics of phenothiazine guest orientations and binding modes. The different binding modes are named SS (secondary-secondary), PP (primary-primary), PS (primary-secondary), and SP (secondary-primary), where the first letter is the orientation of the guest R1 group and the second letter for the guest R2 group. Dashed lines on the guest depict where it is inside the host.

**Fig. 5:**
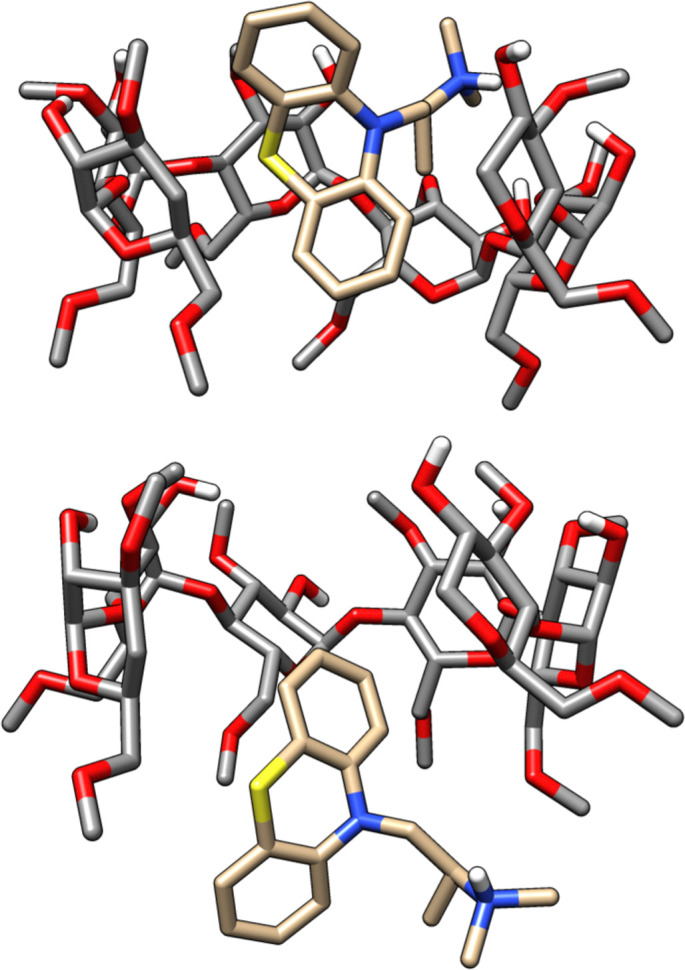
3D structures of the PMT guest in primary and secondary binding modes with bCD or HbCD. PMT binds to bCD and HbCD with the phenothiazine core in the cavity and the protonated amine tail points out to the solution *via* the wider secondary face (top) or the narrower primary face (bottom). A d-glucose monomer from the HbCD host was removed for visualization purposes.

In SAMPL7, experimental characterization of bound conformations *via* NOESY NMR, and some computational approaches, showed that polar head groups of guests can occupy either the primary (primary alcohol opening) or secondary (secondary alcohol opening) face of bCD and functionalized derivatives, and in some cases, both orientations were populated.^21,30,35^ For SAMPL9, we thought that most methods (if not all) needed to account for primary and secondary binding modes of phenothiazines individually, and due to the asymmetry of both guest and host, this may require additional binding modes in each face. For the different binding modes, we use the notation defined previously,^56^ so the binding modes are referred to here as SS (secondary-secondary), PP (primary-primary), PS (primary-secondary), and SP (secondary-primary), where the first word represents the orientation of guest's R1 amine group and the second word denotes the orientation of the R2 functionalization on the phenothiazine core (see Fig. 4). Even with some methods that deploy enhanced sampling techniques, these binding modes may never interconvert in simulations and thus simulations would give inaccurate binding free energies, thus requiring modeling each different metastable binding mode individually. For initial reference calculations, only one pose for each orientation (primary and secondary) was included (see Fig. 5), and will include additional orientations considered in retrospective studies. Moreover, other participants noted considering additional orientations post challenge.

### 2.5 Experimental thermodynamic measurements

Experimental binding measurements for SAMPL9 were obtained *via* isothermal titration calorimetry (ITC) by the Lyle Isaacs^40^ and Michael Gilson^53^ labs. All experimental thermodynamic data for host–guest systems were parsed and processed to be used for the analysis as shown in Table 2. The original experimental data provided by the Isaacs and Gilson groups (as.docx files) can be seen in the SAMPL9 GitHub Repository (https://github.com/samplchallenges/SAMPL9/tree/main/experimental_data).

**Table 2:** Experimental binding data for all host–guest systems

ID	*K*_a_ (M^−1^)	Δ*G* (kcal mol^−1^)	Δ*H* (kcal mol^−1^)	*T*Δ*S*^a^ (kcal mol^−1^)	*n*
WP6-G1^b^^i^	52000.0 ± 2000.0	−6.43 ± 0.02	−8.0 ± 0.2	−1.5 ± 0.2	1.00
WP6-G2^c^^i^	45000000.0 ± 3000000.0	−10.44 ± 0.04	−6.1 ± 0.2	4.3 ± 0.2	1.00
WP6-G3^b^^i^	640000.0 ± 30000.0	−7.91 ± 0.03	−4.7 ± 0.1	3.2 ± 0.1	1.00
WP6-G4^f^^i^	500000.0 ± 20000.0	−7.77 ± 0.02	−4.1 ± 0.1	3.7 ± 0.1	1.00
WP6-G5^g^^i^	9200.0 ± 400.0	−5.40 ± 0.02	−4.0 ± 0.1	1.4 ± 0.1	1.00
WP6-G6^d^^i^	720000.0 ± 50000.0	−7.99 ± 0.04	−7.0 ± 0.2	1.0 ± 0.2	1.00
WP6-G7^e^^i^	130000.0 ± 60000.0	−6.97 ± 0.03	−3.18 ± 0.10	3.8 ± 0.1	1.00
WP6-G8^f^^i^	23300.0 ± 900.0	−5.96 ± 0.02	−9.5 ± 0.3	−3.6 ± 0.3	1.00
WP6-G9^g^^i^	37000.0 ± 4000.0	−6.23 ± 0.06	−5.3 ± 0.2	1.0 ± 0.2	1.00
WP6-G10^c^^i^	16000000.0 ± 1000000.0	−9.83 ± 0.04	−6.2 ± 0.2	3.7 ± 0.2	1.00
WP6-G11^g^^i^	33000.0 ± 1000.0	−6.17 ± 0.02	−5.5 ± 0.2	0.6 ± 0.2	1.00
WP6-G12^h^^i^	89000000.0 ± 5000000.0	−10.84 ± 0.03	−7.4 ± 0.2	3.4 ± 0.2	1.00
WP6-G13^d^^i^	1600000.0 ± 100000.0	−8.47 ± 0.04	−5.0 ± 0.2	3.5 ± 0.2	1.00
bCD-PMZ^j^	4400.0 ± 100.0	−4.97 ± 0.02	−5.9 ± 0.1	−0.9 ± 0.1	1.09
bCD-PMT^j^	1900.0 ± 200.0	−4.47 ± 0.05	−3.9 ± 0.3	0.6 ± 0.3	0.94
bCD-CPZ^j^	9300.0 ± 300.0	−5.41 ± 0.02	−6.4 ± 0.2	−1.0 ± 0.2	0.77
bCD-TDZ^j^	15100.0 ± 800.0	−5.70 ± 0.03	−4.9 ± 0.2	0.8 ± 0.2	1.14
bCD-TFP^j^	5100.0 ± 500.0	−5.06 ± 0.06	−3.9 ± 0.4	1.2 ± 0.4	1.18
HbCD-PMZ^j^	5100.0 ± 200.0	−5.05 ± 0.02	−5.11 ± 0.05	−0.1 ± 0.1	0.99
HbCD-PMT^j^	8400.0 ± 100.0	−5.35 ± 0.01	−4.05 ± 0.06	1.3 ± 0.1	1.06
HbCD-CPZ^j^	9100.0 ± 500.0	−5.40 ± 0.03	−6.0 ± 0.2	−0.6 ± 0.2	0.77
HbCD-TDZ^j^	55000.0 ± 3000.0	−6.46 ± 0.03	−9.28 ± 0.7	−2.8 ± 0.7	0.87
HbCD-TFP^j^	11400.0 ± 600.0	−5.53 ± 0.03	−7.4 ± 0.9	−1.9 ± 0.9	0.56

a All experiments were performed at 298.15 K.

b Direct ITC titration. WP6 (0.1 mM) in the cell with guest (1.0 mM) in the syringe.

c Competitive ITC titration with G7 (0.2 mM) and WP6 (0.1 mM) in the cell with guest (1 mM) in the syringe.

d Direct ITC titration. WP6 (0.05 mM) in the cell with guest (0.5 mM) in the syringe.

e Direct ITC titration. WP6 (0.2 mM) in the cell with guest (2.0 mM) in the syringe.

f Direct ITC titration. WP6 (0.5 mM) in the cell with guest (5.0 mM) in the syringe.

g Direct ITC titration. WP6 (1.0 mM) in the cell with guest (10.0 mM) in the syringe.

h Competitive ITC titration with G7 (0.5 mM) and WP6 (0.1 mM) in the cell with guest (1 mM) in the syringe.

i Experiments were performed in duplicate.

j Experiments were performed in triplicate.

For WP6, ITC experiments were conducted in standard phosphate buffered saline (1× PBS solution containing 137 mM sodium chloride, 2.7 mM potassium chloride, and 10 mM phosphate buffer) at pH 7.4 and at 298.15 K. The concentration of the host used in a cell was between 50 μM and 1 mM, depending on the binding strength of the complex (*i.e.* a lower *K*_a_ value would require a higher concentration of WP6). ITC experiments for bCD complexes were conducted in triplicate on an ITC200 in 25 mM phosphate buffer (without potassium) at pH 7.4 and at 298.15 K. In each case, the guest was in the cell and the host in the syringe, and the concentration of solutions for each complex varied depending on the binding strength of the complex. 2D NOESY (Nuclear Overhauser Effect Spectroscopy) NMR (nuclear magnetic resonance) was conducted on bCD dataset host–guest complexes to identify host and guest atom pairs close enough to detect NOE cross-peaks. For more details, please refer to the associated experimental literature for WP6^40^ and bCD.^53^

## 3 Results and discussion

The results of the SAMPL9 host–guest challenge demonstrate that in general binding free energy predictions for WP6 hosts were more accurate compared to bCD and HbCD. We also observe an inexpensive (although not ranked) and simpler approach based on binding affinity predictions through docking, outperforms all methods for WP6. In this section, we will discuss the results and performance of ranked methods, identify the top performing approaches, compare ranked methods where appropriate, and discuss successes and apparent limitations. In addition, we will compare similar methods when appropriate (including non-ranked and reference calculations) and conduct a sensitivity analysis of changes in the method protocol for accuracy.

We received a total of 22 submissions, 12 for WP6 and 10 for bCD. Of these submissions, we received 4 and 5 ranked submissions for WP6 and bCD, respectively. All submissions and their computed error metrics are listed in Table 3. No group submitted predictions for both WP6 and bCD datasets.

**Table 3:** Computed error metrics of all methods with submissions for SAMPL9 host–guest systems. The error metrics computed include the root mean square error (RMSE), mean absolute error (MAE), signed mean error (ME), coefficient of correlation (*R*^2^), slope (*m*), and Kendall's rank correlation coefficient (Tau). The results shown are for each host category and are sorted in an ascending order based on RMSE. The metrics were computed *via* bootstrapping with replacement and the upper and lower bounds of 95%-percentile confidence intervals are shown in brackets. The statistics in this table do not include the optional host–guest system WP6-G4. Each unique method has an assigned submission ID (sid), a ranked submission is denoted with an asterisk next to the method name, and a reference submission is flagged with a double asterisk

ID	sid	RMSE (kcal mol^−1^)	MAE (kcal mol^−1^)	ME (kcal mol^−1^)	*R* ^2^	*m*	*τ*
WP6							
DOCKING/SMINA/VINARDO	1	1.70 [1.72, 5.27]	1.36 [1.35, 4.53]	−0.39 [−2.91, 2.23]	0.14 [0.00, 0.68]	0.18 [−1.22, 1.61]	0.33 [−0.55, 0.69]
MACHINE-LEARNING/NNET/DRAGON-descriptors*	3	2.04 [1.17, 3.03]	1.57 [0.90, 2.54]	0.62 [−0.64, 1.74]	0.15 [0.00, 0.79]	0.38 [−0.36, 1.13]	0.21 [−0.41, 0.80]
ELIE/GAFF2-ABCG2/TIP3P/MD/MMPBSA*	8	2.49 [1.38, 3.99]	1.93 [1.09, 3.25]	1.93 [0.78, 3.19]	0.40 [0.00, 0.88]	0.66 [−0.10, 1.39]	0.50 [−0.18, 0.87]
EE/Openff-2.0/TIP3P/MD-EE/All_data	5	2.59 [1.75, 3.48]	2.17 [1.38, 3.10]	0.52 [−1.05, 1.96]	0.64 [0.25, 0.89]	1.69 [0.89, 2.38]	0.64 [0.17, 0.93]
EE/Openff-2.0/TIP3P/MD-EE/WL_RL.02_L.01*	4	2.65 [1.71, 3.66]	2.16 [1.34, 3.18]	0.48 [−1.17, 1.90]	0.63 [0.25, 0.89]	1.68 [0.90, 2.41]	0.61 [0.17, 0.93]
DDM/AMOEBA/BAR*	7	2.74 [1.65, 5.14]	1.96 [1.30, 4.21]	−0.60 [−2.72, 1.59]	0.57 [0.10, 0.88]	1.60 [0.55, 2.93]	0.58 [0.05, 0.89]
EE/Openff-2.0/TIP3P/MD-EE/RL_8_only	6	2.82 [1.94, 3.75]	2.44 [1.62, 3.39]	1.19 [−0.45, 2.59]	0.71 [0.34, 0.91]	1.85 [1.11, 2.57]	0.70 [0.33, 1.00]
EE/Openff-2.0/TIP3P/MD-EE/WL_RL.02_L.01/corrected	17	2.96 [2.03, 3.87]	2.61 [1.72, 3.50]	2.08 [0.74, 3.29]	0.54 [0.05, 0.89]	1.27 [0.32, 2.04]	0.48 [−0.12, 0.90]
APR/OFF1.2.0/TIP3P/MD-US/MBAR**	19	3.13 [1.61, 5.00]	2.24 [1.28, 3.90]	1.50 [−0.17, 3.35]	0.09 [0.00, 0.71]	0.47 [−0.95, 1.43]	0.21 [−0.41, 0.70]
vDSSB/GAFF2/OPC3/HREM*	2	3.75 [2.57, 5.89]	3.39 [2.07, 5.28]	3.13 [1.15, 5.08]	0.44 [0.00, 0.83]	1.03 [−0.30, 2.06]	0.44 [−0.25, 0.84]
APR/OFF2.0.0/TIP3P/MD-US/MBAR**	20	3.81 [2.07, 5.91]	2.89 [1.69, 4.70]	2.75 [1.20, 4.57]	0.11 [0.00, 0.79]	0.49 [−1.00, 1.43]	0.21 [−0.42, 0.76]
APR/GAFF2/TIP3P/MD-US/MBAR**	18	6.66 [5.34, 8.04]	6.17 [4.60, 7.72]	6.10 [4.29, 7.70]	0.08 [0.00, 0.71]	0.42 [−0.94, 1.21]	0.27 [−0.37, 0.74]

CD – bCD and HbCD							
ATM/FFENGINE/TIP3P/HREM*	14	1.86 [1.29, 4.29]	1.60 [0.99, 3.69]	−0.94 [−2.93, 1.06]	0.14 [0.00, 0.74]	1.29 [−2.96, 6.10]	0.29 [−0.43, 0.80]
DDM/FEP/MBAR/ParamChem*	16	3.20 [2.65, 3.65]	3.07 [2.48, 3.59]	−0.41 [−2.32, 1.57]	0.13 [0.00, 0.50]	−2.16 [−6.87, 1.17]	−0.11 [−0.60, 0.41]
DD/GROMOS-53A6_glyc/SPC/MD*	11	4.12 [3.06, 5.35]	3.72 [2.50, 5.10]	−3.63 [−5.07, −2.08]	0.00 [0.00, 0.76]	−0.08 [−4.08, 4.24]	0.12 [−0.61, 0.70]
APR/GAFF2/TIP3P/MD-US/MBAR**	21	4.17 [2.90, 5.61]	3.83 [2.53, 5.22]	−3.83 [−5.22, −2.52]	0.22 [0.00, 0.80]	−1.25 [−3.61, 2.26]	−0.20 [−0.79, 0.51]
APR/OFF2.0.0/TIP3P/MD-US/MBAR**	23	4.32 [2.59, 6.06]	3.43 [1.83, 5.33]	−3.43 [−5.28, −1.59]	0.12 [0.00, 0.65]	−1.66 [−6.96, 1.85]	−0.16 [−0.76, 0.41]
APR/OFF1.2.0/TIP3P/MD-US/MBAR**	22	4.41 [2.82, 6.08]	3.74 [2.18, 5.44]	−3.74 [−5.43, −2.08]	0.08 [0.00, 0.64]	−1.20 [−5.99, 2.37]	−0.07 [−0.75, 0.45]
MD/GAFF-RESP/TIP4PEW/MM-PBSA	13	15.33 [13.94, 16.75]	15.23 [13.81, 16.63]	15.23 [13.81, 16.63]	0.78 [0.08, 0.96]	3.88 [1.15, 6.40]	0.45 [−0.15, 0.95]
MD/GAFF-RESP/TIP4PEW/MM-GBSA	12	18.47 [17.17, 19.78]	18.40 [17.06, 19.71]	18.40 [17.06, 19.71]	0.65 [0.06, 0.92]	3.25 [0.80, 7.10]	0.64 [−0.05, 1.00]
MD/GAFF-RESP/TIP4PEW/MM-GBSA_2	15	18.47 [17.15, 19.75]	18.40 [17.03, 19.69]	18.40 [17.03, 19.69]	0.65 [0.05, 0.92]	3.25 [0.74, 7.17]	0.64 [−0.05, 1.00]
PMF/GAFF-RESP/TIP4PEW/SMD*	10	27.26 [22.73, 31.33]	26.38 [22.10, 30.74]	26.38 [22.10, 30.74]	0.00 [0.00, 0.54]	−0.78 [−11.99, 13.21]	0.02 [−0.55, 0.56]

### 3.1 WP6 – ranked methods

**Fig. 6:**
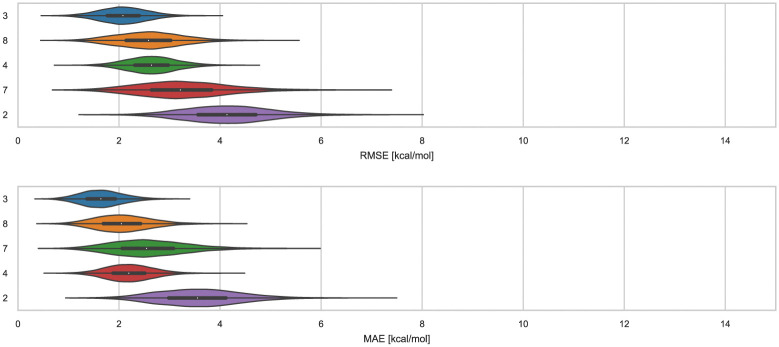
RMSE and MAE of ranked methods for the WP6 dataset. Violin plots showing the distribution of performance for predicting binding free energies of guests (optional system WP6-G4 not included) for WP6. The median is indicated by a white circle in the horizontal black bar of the violin plot. The black bars in the violins represent the first and third quartiles. RMSE, MAE, and their associated plots were generated by bootstrapping samples with replacement.

Here, we compare error and correlation metrics to evaluate the performance of ranked methods for WP6. The computed error metrics (RMSE and MAE) for ranked methods ranged from about 2.04 to 3.75 kcal mol^−1^ and 1.57 to 3.39 kcal mol^−1^, respectively. The top performing method for the WP6 challenge by RMSE and MAE was a knowledge based method MACHINE-LEARNING/NNET/DRAGON-descriptors, with RMSE of 2.04 kcal mol^−1^ and MAE of 1.57 kcal mol^−1^. Fig. 6 shows the performance of the remaining methods ELIE/GAFF2-ABCG2/TIP3P/MD/MMPBSA, EE/Openff-2.0/TIP3P/MD-EE/WL_RL.02_L.01, DDM/AMOEBA/BAR, and vDSSB/GAFF2/OPC3/HREM, sorted by RMSE.

The fact that MACHINE-LEARNING/NNET/DRAGON-descriptors had the best error metrics (though statistically similar to the next method by ranking) highlights an advantage of using machine learning approaches when sufficient experimental data is available. The other ranked methods for WP6 were approaches relying on MD simulations that would require much more time and computing resources compared to this ML approach, yet the MD based approaches obtained BFEs of similar or worse accuracy. In each of the last three SAMPL iterations, we have received one submission which made use of an ML-based method for predicting BFEs with some success. In this span, these ML approaches have achieved RMSEs of 2.39 kcal mol^−1^ or below (1.67,^91^ 2.39,^31^ and 2.04 kcal mol^−1^) for Octa-Acids (SAMPL7 and SAMPL8) and WP6 hosts (SAMPL9), respectively. In the more recent challenges (SAMPL8 and SAMPL9), the ML-based predictions have been among the best performing models as measured by the RMSE and MAE error metrics. However, correlation metrics of ML-based models in SAMPL have been inconsistent and relatively poor, with *R*^2^ values of 0.01,^30^ 0.60,^31^ and 0.15 in the present challenge.

**Fig. 7:**
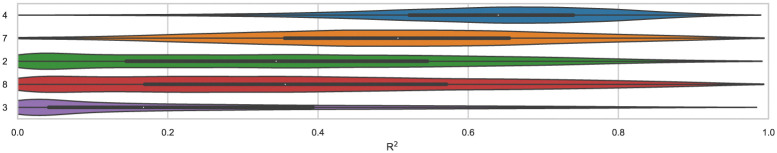
*R* ^2^ of ranked methods for the WP6 dataset. Violin plots showing the distribution of performance for predicting binding free energies of guests (optional system WP6-G4 not included) for WP6. The median is indicated by a white circle in the horizontal black bar of the violin plot. The black bars in the violins represent first and third quartiles. *R*^2^ and their associated plots were generated by bootstrapping samples with replacement.

We find that ranked methods using force fields (OpenFF2.0.0, AMOEBA, and GAFF2) had better *R*^2^ than ML and docking-based methods, and only the top two approaches had values greater than 0.50. Fig. 7 shows *R*^2^ for ranked methods with the highest coefficient of determination coming from EE/Openff-2.0/TIP3P/MD-EE/WL_RL.02_L.01 followed by DDM/AMOEBA/BAR and vDSSB/GAFF2/OPC3/HREM. The MACHINE-LEARNING/NNET/DRAGON-descriptors had the lowest *R*^2^ value of 0.15. Ranking molecules by binding free energy or affinity (from strongest to weakest) to identify and prioritize potential leads is also of great importance, perhaps more important than accuracy, since in a drug discovery setting free energy methods would likely be employed to help prioritize synthesis. In such a workflow, correct rank ordering can be more important than absolute accuracy. When only considering ranked methods for WP6, 3 out of 5 methods had *τ* values greater than 0.50, and all methods used a force field (Table 4). The EE/Openff-2.0/TIP3P/MD-EE/WL_RL.02_L.01 and DDM/AMOEBA/BAR methods had the highest *τ* values of 0.61 and 0.58, followed by 0.50 and 0.44 for methods using GAFF2 (ELIE/GAFF2-ABCG2/TIP3P/MD/MMPBSA and vDSSB/GAFF2/OPC3/HREM respectively), while MACHINE-LEARNING/NNET/DRAGON-descriptors had the lowest at 0.21.

**Table 4:** Computed error metrics of ranked submissions for SAMPL9 WP6 host–guest systems The error metrics computed include the root mean square error (RMSE), mean absolute error (MAE), signed mean error (ME), coefficient of correlation (*R*^2^), slope (*m*), and Kendall's rank correlation coefficient (Tau). The results shown are for the WP6 host category and are sorted in an ascending order based on RMSE. The metrics were computed *via* bootstrapping with replacement and the upper and lower bounds of 95%-percentile confidence intervals are shown in brackets. The statistics in this table do not include the optional host–guest system WP6-G4. Each unique method has an assigned submission ID (sid)

ID	sid	RMSE (kcal mol^−1^)	MAE (kcal mol^−1^)	ME (kcal mol^−1^)	*R* ^2^	*m*	*τ*
**WP6**							
MACHINE-LEARNING/NNET/DRAGON-descriptors	3	2.04 [1.17, 3.01]	1.57 [0.90, 2.52]	0.62 [−0.66, 1.76]	0.15 [0.00, 0.79]	0.38 [−0.38, 1.12]	0.21 [−0.42, 0.80]
ELIE/GAFF2-ABCG2/TIP3P/MD/MMPBSA	8	2.49 [1.38, 3.93]	1.93 [1.09, 3.23]	1.93 [0.80, 3.16]	0.40 [0.00, 0.88]	0.66 [−0.09, 1.40]	0.50 [−0.16, 0.87]
EE/Openff-2.0/TIP3P/MD-EE/WL_RL.02_L.01	4	2.65 [1.68, 3.63]	2.16 [1.30, 3.14]	0.48 [−1.12, 1.90]	0.63 [0.24, 0.89]	1.68 [0.89, 2.39]	0.61 [0.16, 0.93]
DDM/AMOEBA/BAR	7	2.74 [1.66, 5.22]	1.96 [1.28, 4.25]	−0.60 [−2.73, 1.62]	0.57 [0.09, 0.88]	1.60 [0.57, 2.93]	0.58 [0.05, 0.87]
vDSSB/GAFF2/OPC3/HREM	2	3.75 [2.59, 5.87]	3.39 [2.06, 5.26]	3.13 [1.15, 5.05]	0.44 [0.00, 0.83]	1.03 [−0.31, 2.07]	0.44 [−0.23, 0.85]

Predictions with methods using the AMOEBA force field have been among the most accurate (if not the most accurate) in SAMPL7 (OA, exoOA, and TrimerTrip) and SAMPL8 (TEMOA and TEETOA). These results have shown the importance of modeling explicit polarization for host–guest systems, particularly when the host or guest (or both) are highly polar or charged; such as host–guest systems present in SAMPL7-8. For WP6 in SAMPL9, AMOEBA had considerable success in achieving one of the highest correlation metrics (Fig. 7). In terms of accuracy, DDM/AMOEBA/BAR was not among the top 2 methods and did not have superior performance across all metrics, for a change. Perhaps, this is because other issues were more accuracy-limiting for this particular set of compounds. In the past, torsion tuning of the host has led to improved agreement with experimental BFE measurements.

### 3.2 The force field limits the ability to model some host–guest systems

WP6-G4 was made an optional system because guest G4 had silicon and present modeling difficulties with some tools. Nevertheless, 4 of 5 ranked submissions and an additional 4 non-ranked submissions included these predictions. Predicted binding free energies had wide variability with ΔΔ*G* errors ranging from −3.58 to 3.02 kcal mol^−1^ (see Table S3, ESI†). MACHINE-LEARNING/NNET/DRAGON-descriptors was the only method to predict WP6-G4 within 1 kcal mol^−1^ of the experiment (−7.77 ± 0.02 kcal mol^−1^), with a slightly more favorable binding free energy as shown by a ΔΔ*G* error of −0.57 kcal mol^−1^. Of all other methods in Table S3 (ESI†), only one other method (vDSSB/GAFF2/OPC3/HREM) had a ΔΔ*G* error below 2 kcal mol^−1^. The rest of the methods with predictions for WP6-G4 had ΔΔ*G* errors above an absolute value of 2.66 kcal mol^−1^. The EE methods used GAFF2.11 (OpenFF 2.0.0 parameters were unavailable for WP6-G4 silane group) and predicted binding affinities were too favorable with errors in the −3 kcal mol^−1^ range. The results demonstrate potential force field limitations and the ability of an ML model (based on molecular descriptors) to accurately describe and predict BFEs of silicon-based small molecules like WP6-G4.

Some of the methods are physics-based and attempt to calculate true binding free energies. In theory, such methods (when using the same underlying energy model) should give consistent predictions even if the details of the approach are somewhat different. However, in practice, this has not always been the case. Indeed, after surveying the predicted binding free energies for WP6-Guest systems, we find that predictions for the same host–guest complex varied between methods, in some cases, the absolute ΔΔ*G* error had a range of nearly 10 kcal mol^−1^ (Fig. S3, ESI†), illustrating predictive variability across methods for most WP6 systems. This observation remains true for the dataset when we consider non-ranked approaches as well, thus a total of 12 methods. For example, the WP6-G8 complex had the highest RMSE of 3.94 kcal mol^−1^ and may not come as a surprise for methods using generalized force fields since the guest contains a sulfonate group (Fig. 1). Development and/or optimization of parameters for sulfur containing small molecules have been ongoing work for small molecule force fields.^92–94^ These results hint towards force field limitations and/or inaccuracies, but other factors such as sampling, solvent effects, or buffer effects can't be eliminated.

The closest agreement across methods was for WP6-G10, WP6-G3, and WP6-G11, with RMSEs below 2 kcal mol^−1^ (1.38, 1.79, and 1.84 kcal mol^−1^ respectively); while RMSEs on the remaining WP6–guest complexes were between 2 to 4 kcal mol^−1^ (Table 5). WP6 guests that had the highest RMSE (between 3 to 4 kcal mol^−1^, Table 5) were WP6-G13, WP6-G9, WP6-G1, WP6-G12, and WP6-G8. These guests contain an unusual ring system (WP6-G1), an adamantane like core (WP6-G9, WP6-G12), or a nitrogen conjugated system (WP6-G13). In SAMPL7,^30^ calculations for TrimerTrip with adamantane-like molecules (clip-g11, clip-g6, clip-g9, clip-g10) had poor predictive accuracy with RMSE values greater than 4 kcal mol^−1^. In addition, guest clip-g18 of SAMPL7 is the same as guest WP6-G13; this guest contains a nitrogen conjugated system and had among the highest RMSEs (across methods) of 6.70 and 3.33 kcal mol^−1^, respectively. Thus, this particular guest was also challenging in TrimerTrip in SAMPL7. We note that predictive accuracy for SAMPL7 TrimerTrip binding was in general worse, as measured by RMSE. Some participants argued that SAMPL7 was particularly difficult or challenging due to the additional flexibility and poor sampling of the acyclic clip-like host. However, whether the inaccuracies for adamantane-like and nitrogen conjugated system molecules were a result of force field limitations, sampling issues, other environmental factors modeled (buffer conditions, protonation state, *etc*), or a combination of remains inconclusive and would require further investigation.

**Table 5:** Error metrics of each SAMPL9 host–guest system considering only ranked predictions. The error metrics computed (across all submissions) include the root mean square error (RMSE), mean absolute error (MAE), and signed mean error (ME). The results shown are for each host–guest system considering only ranked predictions and are sorted in an ascending order based on MAE; they provide a measure of the difficulty of each particular host–guest complex. The metrics were computed *via* bootstrapping with replacement and the upper and lower bounds of 95%-percentile confidence intervals are shown in brackets. The statistics do not include optional host–guest system WP6-G4

ID	RMSE (kcal mol^−1^)	MAE (kcal mol^−1^)	ME (kcal mol^−1^)
WP6-G10	1.38 [0.36, 3.89]	0.97 [0.30, 3.19]	0.83 [−1.01, 2.89]
WP6-G3	1.79 [0.56, 4.23]	1.48 [0.46, 3.59]	0.74 [−1.60, 2.94]
WP6-G11	1.84 [0.96, 3.88]	1.68 [0.78, 3.48]	1.44 [−0.73, 3.24]
WP6-G5	2.91 [0.69, 5.35]	1.95 [0.57, 4.66]	1.21 [−1.35, 4.18]
WP6-G6	2.22 [0.77, 4.74]	1.97 [0.64, 4.09]	0.53 [−2.05, 3.11]
WP6-G2	2.19 [1.01, 4.21]	2.00 [0.82, 3.87]	2.00 [0.16, 3.83]
WP6-G7	2.52 [0.90, 5.20]	2.10 [0.70, 4.42]	1.28 [−1.27, 3.84]
WP6-G13	3.33 [0.80, 6.66]	2.41 [0.65, 5.44]	1.79 [−1.04, 5.08]
WP6-G9	3.16 [1.47, 5.89]	2.61 [1.15, 5.14]	1.81 [−1.14, 4.69]
WP6-G1	3.25 [1.50, 5.12]	2.74 [1.26, 4.83]	0.13 [−3.23, 3.27]
WP6-G12	3.63 [1.76, 6.23]	2.91 [1.35, 5.52]	1.13 [−2.28, 4.62]
WP6-G8	3.94 [2.10, 6.59]	3.60 [1.75, 6.03]	0.46 [−3.44, 4.26]
bCD-TDZ	9.58 [0.95, 16.19]	6.09 [0.84, 14.33]	3.88 [−2.68, 13.90]
bCD-PMT	10.75 [1.11, 18.48]	6.44 [0.85, 16.47]	5.47 [−0.93, 16.09]
HbCD-PMT	10.17 [2.16, 17.03]	6.94 [1.33, 15.56]	2.85 [−3.92, 13.78]
HbCD-TDZ	10.62 [2.20, 17.89]	7.29 [1.92, 16.30]	2.98 [−3.67, 14.52]
bCD-PMZ	10.10 [2.71, 16.68]	7.32 [2.17, 15.34]	4.22 [−3.10, 14.04]
bCD-CPZ	14.11 [2.10, 23.98]	9.13 [2.08, 21.32]	6.78 [−2.96, 21.21]
HbCD-CPZ	18.16 [1.76, 31.24]	10.68 [1.01, 27.77]	7.34 [−3.25, 26.28]
bCD-TFP	16.89 [2.82, 28.78]	10.84 [2.35, 25.80]	8.31 [−2.87, 25.23]
HbCD-TFP	17.07 [2.49, 29.07]	10.91 [2.31, 25.94]	5.84 [−4.50, 24.32]
HbCD-PMZ	17.43 [2.90, 29.69]	11.27 [2.89, 26.46]	5.85 [−4.46, 24.90]

We cross-compared methods employing different force fields to assess how much the force field and/or method impacted computed binding free energies. In particular, the RMS difference between free energies calculated with different reference BFE calculation protocols with the APR method provides evidence that force-field selection, may dictate the level of success for ABFEs for certain systems. As described earlier in Section 2.4, these methods differed only in the force-field used to parameterize the host–guest complex. In the APR approach, different force fields led to different calculated binding free energies for the WP6 dataset (Fig. S4, ESI†). Using GAFF2 with the APR approach to predict the affinity of WP6 complexes resulted in a greater error compared to OpenFF 1.2.0 and OpenFF 2.0.0, with RMS differences of 5.38 (GAFF2 *versus* OpenFF 1.2.0) and 4.19 kcal mol^−1^ (GAFF2 *versus* OpenFF 2.0.0), respectively. In comparison, the different iterations of OpenFF (OpenFF 1.2.0 and OpenFF 2.0.0) with the APR approach gave a lower RMS difference of 1.62 kcal mol^−1^. In contrast, force field differences did not result in disparate predictions for the bCD/HbCD dataset with the APR approach. Using GAFF2 with the APR approach compared to OpenFF 1.2.0 and OpenFF 2.0.0 had RMS differences of 1.22 (GAFF2 *versus* OpenFF 1.2.0) and 1.43 kcal mol^−1^ (GAFF2 *versus* OpenFF 2.0.0), while the RMS difference between OpenFF versions was 0.52 kcal mol^−1^. On the other hand, using GAFF2 with a different method (*i.e.* ELIE/GAFF2-ABCG2/TIP3P/MD/MMPBSA) achieved better performance compared to APR, and we also observed that the use of OpenFF2.0.0 with a different method (*i.e.* EE/Openff-2.0/TIP3P/MD-EE/All_data) had a better statistical performance. In the case of the latter, however, we speculate the discrepancy may be due to 3 systems in particular (WP6-G1, WP6-G5, and WP6-G8) that were consistent outliers between the two approaches (discussed in Section 3.3).

### 3.3 Nearly equivalent methods predicting WP6 binding affinity have high agreement, and reveal consistent outliers

In-house reference calculations varied only in the energy model chosen while all other simulation parameters remained constant. As seen in Table 3, the performance of a reference method with a particular energy model varied across hosts. For example, we see greater predictive accuracy for WP6 systems when using OpenFF 1.2.0 followed by the newer OpenFF 2.0.0, as opposed to GAFF2.11. Another group used an EE approach with OpenFF 2.0.0 and submitted additional predictions with variations of the method (method names with EE in Table 3), granting an opportunity to cross compare EE methods with reference method (APR/OFF2.0.0/TIP3P/MD-US/MBAR) for the WP6 dataset. Briefly, differences in the EE methods were in the application of Wang–Landau (WL) condition thresholds (0.02 for host–guest complex and 0.01 for guest), whether all data were used in weighted averages to include contributions of host and guest microstates to BFEs, or if all host microstates are considered, or when only the WP6 host with a formal charge of −8 is considered. For more details on these methods, readers are directed to https://github.com/samplchallenges/SAMPL9/main/host_guest/Analysis/Submissions/WP6/ and https://vvoelz.github.io/sampl9-voelzlab/.

**Fig. 8:**
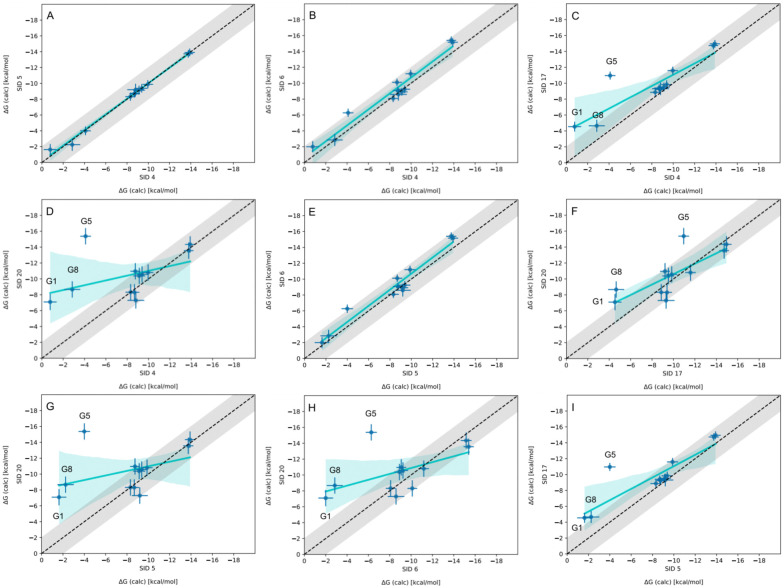
Correlation plots for methods using OpenFF2.0.0 on the WP6 dataset. Plots comparing methods using OpenFF2.0.0 for predicting BFEs (kcal mol^−1^) in SAMPL9 WP6 host–guest challenge. The gray shaded region represents 2 kcal mol^−1^ error, the cyan solid line is linear regression, and the cyan shaded region is the confidence interval. The submission IDs (SIDs) are shown in the respective x and y axes. Methods being compared in each plot are SIDs: (A) 4 and 5, (B) 4 and 6, (C) 4 and 17, (D) 4 and 20, (E) 5 and 6, (F) 17 and 20, (G) 5 and 20, (H) 6 and 20, and (I) 5 and 17.

When comparing BFE predictions of reference calculations and the EE methods, there is agreement between the approaches. The majority of BFE predictions from these two methods for WP6 systems are within 2 kcal mol^−1^ of one another, however, 3 common outliers persist with guests WP6-G1, WP6-G5, and WP6-G8 (Fig. 8). Major differences between reference calculations and EE approaches are in the microstates of guests and host used, and the sampling technique. From the correlation plots (Fig. 8C, D, F–I), BFEs for guests G1, G5, and G8 are sensitive to the system set-up and how the guest, or host, or both are modeled. The sensitivity of these systems would suggest some unique molecular recognition characteristics. Another possibility for the BFE inconsistencies can be due to inadequate sampling of the WP6 host or guests (particularly G1 and G8). In future work, it would be interesting to see whether using the same starting structures are used in EE methods, whether these outliers persist or whether there is better agreement with the reference approach.

### 3.4 Cyclodextrins and phenothiazines – ranked methods

**Fig. 9:**
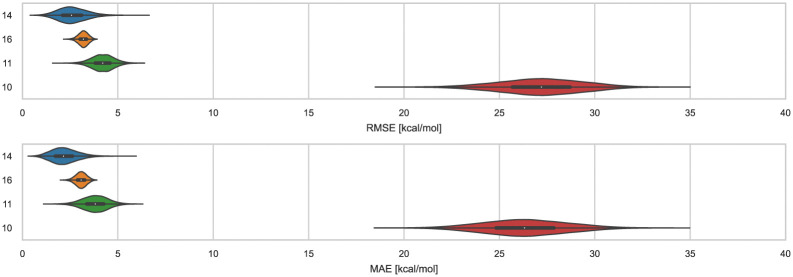
RMSE and MAE of ranked methods for the combined cyclodextrin dataset. Violin plots showing the distribution of performance for predicting binding free energies of guests for bCD and HbCD. The median is indicated by a white circle in the horizontal black bar of the violin plot. The black bars in the violins represent first and third quartiles. RMSE, MAE, and their associated plots were generated by bootstrapping samples with replacement.

Distinct alchemical methods differing in approach, energy model, and/or sampling technique were used to submit ranked predictions for the cyclodextrin and phenothiazine challenge. The methods used were ATM, DDM, and potential of mean force (PMF). The accuracy of ranked methods as measured by RMSE and MAE ranged from 1.86 to 27.26 kcal mol^−1^, and 1.60 to 26.38 kcal mol^−1^, respectively. The ranked methods were ordered based on these accuracy metrics as shown in Fig. 9, with ATM/FFENGINE/TIP3P/HREM emerging as the top performing method. Ranked 2nd and 3rd were DDM/FEP/MBAR/ParamChem and DD/GROMOS-53A6_glyc/SPC/MD (Fig. 9). The PMF/GAFF-RESP/TIP4PEW/SMD ranked 4th, estimating binding free energies that were too favorable and leading to RMSE and MAE values of 27.26 and 26.38 kcal mol^−1^, respectively. Due to the multiple differences between methods employed, it is difficult to compare or determine if and what factor or combination of factors contributed to the differences in model BFE accuracy.

**Fig. 10:**
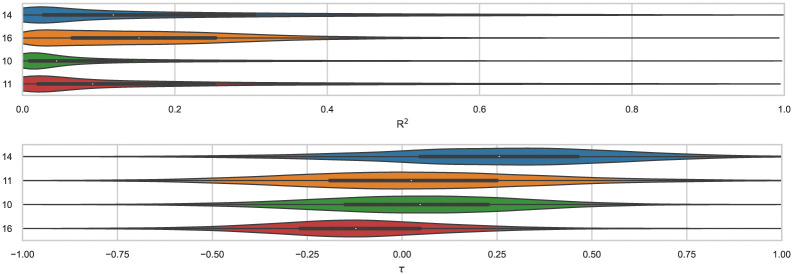
*R* ^2^ and *τ* of ranked methods for the combined cyclodextrin dataset. Violin plots show the distribution of correlation and ranking performance of ranked predictive models for binding free energies of guests for bCD and HbCD. The median is indicated by a white circle in the horizontal black bar of the violin plot. The black bars in the violins represent first and third quartiles. *R*^2^, *τ*, and their associated plots were generated by bootstrapping samples with replacement.

Correlation statistics for ranked methods in the cyclodextrin phenothiazine challenge were low, with the *R*^2^ values ranging from 0.00 to 0.14 and *τ* −0.11 to 0.29 (Fig. 10). The ATM/FFENGINE/TIP3P/HREM method achieved the best *R*^2^ and *τ* values of 0.14 and 0.29 among ranked approaches. When considering ranked and non-ranked methods, end-point calculations with MM-PBSA/GBSA had the highest *R*^2^ and *τ* values (Table 3). The overall low correlation is likely at least partially associated with the narrow dynamic range of the dataset, as we discuss further below.

### 3.5 Multiple binding orientations are necessary for accurate cyclodextrin-phenothiazine BFEs

The asymmetry of bCD and HbCD, as well as the asymmetry of some phenothiazine guests, necessitates accounting for binding modes where the phenothiazine alkyl amine side chains are occupying distinct primary and secondary face binding orientations; otherwise, these may not interconvert at simulation time scales. The binding free energy of each binding mode can be calculated separately and the results combined to obtain an overall binding free energy of the host–guest system.^51,56,71^ Most participants only modeled one binding mode, except ATM/FFENGINE/TIP3P/HREM (up to 8 binding modes depending on guest) and APR reference calculations (1 primary and 1 secondary binding mode). All methods obtained starting poses and structures *via* different docking software (GalaxyDock, MOE, OEDock, and Maestro).

The phenothiazine moiety of TFP, CPZ, and TDZ had functionalization; thus, these substituents would also occupy distinct primary and secondary face orientations giving rise to separate binding modes (see Fig. 4). In addition, the nitrogen on the phenothiazine moiety of TFP, CPZ, and TDZ undergoes induced chirality, so binding modes to account for these conformational enantiomers may also be necessary.^56^ Considering all primary and secondary face orientations due to the asymmetric nature of these host–guest systems, the number of binding modes that may not interconvert or sampled with some methods becomes 8. Indeed, the top performing ATM/FFENGINE/TIP3P/HREM method did exactly that (while also accommodating guests with multiple protomer states) with success.

For all methods where a single binding mode was used and with reference calculations that considered two binding modes, the models have RMSE and MAE values higher than 3 kcal mol^−1^. In some cases, it is true that using only the most favorable binding mode (which contributes the most to the overall BFE) and ignoring other binding modes yields accurate predictions and incurs only small and negligible errors. However, the most favorable binding mode cannot necessarily be predicted *a priori* making this strategy often impractical. The SAMPL9 bCD dataset results showcase instances where predictions can be accurate even without considering additional binding modes, and instances where that is not the case, highlighting the remaining importance of methods for binding pose selection and thus binding mode prediction and/or sampling of binding modes.

The method using the GROMOS 53A6_GLYC_ force field (DD/GROMOS-53A6_glyc/SPC/MD), optimized for hexopyranose-based carbohydrate molecules,^95^ had some success while using a single binding mode. For bCD-PMT and HbCD-PMT, the model predicted their BFEs within 0.5 kcal mol^−1^; with predicted values of −4.9 and −5.0 kcal mol^−1^ (experimental values were −4.47 and −5.35 kcal mol^−1^) respectively. The accuracy achieved with GROMOS 53*A*6_GLYC_ with a single binding mode, points towards a dominant binding mode for the PMT guest which is consistent with the experimental observation that PMT binds the secondary face.^48^ The participants for this method (DD/GROMOS-53A6_glyc/SPC/MD) noted that other binding modes may be necessary from some systems and may be considered in future work. Most of the other systems in the bCD dataset were predicted to be non-binders (0 kcal mol^−1^) with this method. It would be interesting to see if considering other binding modes in these systems with 53*A*6_GLYC_ can provide further evidence of certain binding modes as dominant or have greater weight on the BFE.

A different approach (DDM/FEP/MBAR/ParamChem) generated CGenFF parameters for each host–guest system *via* the ParamChem webserver. BFEs for all bCD systems were estimated to be too favorable by 2.0 to 4.0 kcal mol^−1^, and all HbCD systems were estimated to be too unfavorable by 3.0 to 4.5 kcal mol^−1^. Both the CGenFF and 53*A*6_GLYC_ models' statistical performance was similar relative to one another and to that of reference calculations. In other words, the choice of CGenFF parameters did not result in a significant difference in predictive power overall. However, a closer look at BFE predictions with 53*A*6_GLYC_ (DD/GROMOS-53A6_glyc/SPC/MD) for specific systems compared to experiments show instances where on the one hand modeling a dominant binding mode had considerable success, and on the other hand, poorly predicted BFEs where predicted binding free energies seem to err in a way consistent with the use of an incorrect or non-dominant binding mode.

We note that two methods (DD/GROMOS-53A6_glyc/SPC/MD and DDM/FEP/MBAR/ParamChem) predicted some phenothiazine guests to be weak or non-binders to the cyclodextrin hosts (bCD and HbCD), with BFEs ranging from −2 to 0 kcal mol^−1^. BFEs reflecting non-binding or weak binding may be associated with phenothiazine modeled in the smaller face (primary orientation) of either host, but more so with the narrower HbCD, while more favorable BFEs should result for a binding mode in the secondary face. The effect of the modeled phenothiazine binding mode would be more relevant for TFP, CPZ, and TDZ which have larger substituents, so a PP binding mode with both substituents oriented towards the smaller primary face would be unfavorable due to sterics. A binding mode with the pose occupying primarily the secondary face would be consistent with the experimental results and the previously predicted PMT binding mode,^48^ which was suggested to have the phenothiazine core at the larger secondary face and intruding deep into the primary face while the alkyl amine side chain also occupied the secondary face of bCD (Fig. 5). Predicting BFEs appeared to be challenging for most approaches, particularly for HbCD. In general, most methods tended to produce BFEs which were too positive relative to experimental values, and the origin of this discrepancy is not clear. However, it is tempting to speculate that underestimations are due to modeling of a non-dominant binding mode or conformational trapping of the host and/or guest.

As mentioned in Sections 2.5, 2D NOESY NMR was used to obtain NOE peaks for cyclodextrin complexes, providing a cross check for modeling and giving insight on host–guest interactions. As expected, experimental NOEs revealed at least one end of the phenothiazine core is at the secondary face of the host while the opposite end penetrates deep towards the primary face for all but one complex.^53^ In addition, NOEs also suggest that the bulky cationic side chain ends of the TDZ and TFP guests reside at the primary face of both hosts. The authors speculate that all cationic side chains reside at the primary face of the hosts, but only TDZ's and TFP's bulkier side chains are tightly fixed in the host binding site to generate observable NOEs.^53^ To our knowledge, whether the functionalized region of the phenothiazine core for TFP, TDZ, and CPZ reside at the secondary or primary face remains unknown and unexplored, experimentally.

### 3.6 Binding free energy predictions based on docking outperform or are comparable to more expensive approaches

Docking is commonly used for initial screening to identify potential ligand candidates and leads, to then be assessed by more computationally expensive approaches to prioritize and advance ligands through the pipeline. In SAMPL, docking has rarely been used to predict binding strength, although docking is commonly used in some methods to obtain a starting pose of a complex. Recently, a systematic assessment examined how well docking scores can predict BFEs,^24^ using the Autodock4^96^ and Vina^97^ docking programs. This retrospective study examined all host–guest systems from SAMPL6 to SAMPL8. In this study, docking (with Autodock4) outperformed most methods including expensive and complex MD-based or QM-based computational methods used in those SAMPL iterations.^24^ For SAMPL9, the same authors submitted predictions obtained using Vina, where binding scores were calculated using the smina code (from https://sourceforge.net/projects/smina/) and the improved vinardo scoring function (method DOCKING/SMINA/VINARDO). Similar to the results in ref. 24, when considering all methods including non-ranked, DOCKING/SMINA/VINARDO outperforms most methods in several of the calculated statistical metrics (see Table 3). The RMSE and MAE (1.70 and 1.36 kcal mol^−1^, respectively) for this approach were the best of all submissions, but had a poor *R*^2^ of 0.14. At ranking WP6 systems, DOCKING/SMINA/VINARDO performed at about mid-tier among challenge submissions with a *τ* of 0.33. The reported performance of scoring *via* docking across different host–guest system categories (from SAMPL6 through SAMPL9) suggests that docking may be suitable for supramolecular system studies and design and indeed could potentially serve as a reference model in future SAMPL challenges.

To some extent, we find that both docking and ML methods do particularly well in terms of error statistics at the expense of correlation, as we discussed in Section 3.7.

### 3.7 Host–guest affinity ranking was relatively difficult across methods

In general, recent SAMPL challenges highlight that accurately ranking host–guest binding free energies remains difficult. In addition, methods tend to fare better at identifying the tightest binder for each dataset, but methods tend to be unable to predict the weakest binder. When considering all predictions (ranked and non-ranked) in each dataset (WP6 and CD), methods performed better at ranking WP6 complexes by affinity as opposed to CD. As measured by *τ*, ranking ranged between 0.18 to 0.66 for WP6 and between −0.19 to 0.64 for CD. For WP6, 5 out of 12 (approximately 42%) methods achieved *τ* values over 0.50, while 2 out of 10 (20%) for CD (see Table 3). The top performing methods, as determined *via* MAE and RMSE, in each host category did not give the best *τ*.

Given that the accuracy of ranking host–guest complexes was low in SAMPL9, we surveyed the capability of a method (ranked or non-ranked) to predict the strongest and weakest binder correctly. For WP6, 5 out of 12 methods (EE/Openff-2.0/TIP3P/MD-EE/All_data, EE/Openff-2.0/TIP3P/MD-EE/WL_RL.02_L.01, EE/Openff-2.0/TIP3P/MD-EE/WL_RL.02_L.01/corrected, vDSSB/GAFF2/OPC3/HREM, and APR/GAFF2/TIP3P/MD-US/MBAR) predicted WP6-G12 as the strongest binder and 2 out of 12 methods (APR/OFF1.2.0/TIP3P/MD-US/MBAR, and APR/OFF2.0.0/TIP3P/MD-US/MBAR) predicted WP6-G5 as the weakest binder. No method correctly predicted the strongest or weakest binders for bCD and HbCD systems.

Overall, we find that both docking and ML-based methods do particularly well in terms of error statistics on WP6 host–guest binding, but have relatively lower correlation statistics than force field based methods. This may be because the larger degree of empirical training (to binding data) in ML and docking methods results in predictions which tend to be near the center of the true range of affinities, resulting in good error statistics, but at the expense of correlation with experimental values. (As a thought experiment, consider a simple null model which predicted that all binding free energies were equal but had the correct mean; such a model could achieve a relatively low error but the correlation would be zero).

### 3.8 Realistic model performance based on the dynamic range and uncertainty of the experimental measurements

We computed several metrics to assess the success of a method and compare methods by accuracy and correlation statistics with the experiment. However, the usefulness and interpretability of statistical performance measurements obtained depend on the experimental dynamic range, the experimental error, and the number of data points.^98,99^ Accurate predictive models become more difficult to build or assess when the experimental dynamic range is small or if there are large experimental uncertainties. In the SAMPL9 host–guest challenge, the bCD dataset has a low dynamic range; thus, we obtain a realistic or upper-limit of model performance by quantifying the experimental uncertainty^98,99^ using the equation:
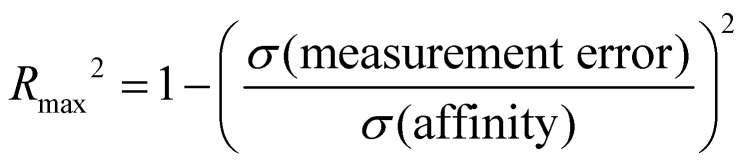
where *R*_max_^2^ is the upper-limit *R*^2^ that can be obtained for a dataset with a standard deviation of affinities (*σ*(affinity)) and experimental uncertainty (*σ*(measurement error)). The bCD dataset has a dynamic range of 1.99 kcal mol^−1^ (−4.47 to −6.46 kcal mol^−1^), with *σ*(affinity) of 0.545 and *σ*(measurement error) of 0.015. Although the dynamic range is lower than the recommended 5–7 kcal mol^−1^ for a dataset,^99^ with a very low experimental uncertainty we can expect a *R*_max_^2^ of 0.999. Similarly, with a WP6 experimental *σ*(measurement error) of 0.012, a *σ*(affinity) of 1.803, and a dynamic range of 5.44 kcal mol^−1^ (−5.40 to −10.84 kcal mol^−1^), we can expect a *R*_max_^2^ of 1.000. However, it would be reasonable to speculate that the experimental uncertainties were underestimated, and if we guess a placeholder value of 0.5 as the *σ*(measurement error), *R*_max_^2^ would be 0.158 and 0.923 for the two datasets (bCD and WP6), respectively. Using this placeholder value for the bCD dataset results in *R*_max_^2^ value (0.158) that is lower than what some participants achieved (see Table 3). Therefore, it is likely the true uncertainty is less than 0.5. A *σ*(measurement error) value of 0.12 would yield *R*_max_^2^ of 0.779, roughly matching the best *R*^2^ a method achieved (0.78).

## 4 Conclusions and lessons learned

The SAMPL9 host–guest challenge provided a platform to test the accuracy and reliability of computational methods for predicting BFEs on a set of 13 guests to WP6, and 5 phenothiazine based drugs for bCD and HbCD. Participants had some level of success on the WP6 dataset whereas the cyclodextrin challenge was particularly troublesome. In general, the ranked BFE predictions for WP6 were more accurate and had a better correlation with the experiment compared to the cyclodextrin-phenothiazine set. Correlation metrics for ranked methods in the cyclodextrin dataset were poor with *R*^2^ and *τ* values well below 0.5. Overall, the predictive performance of the majority of methods in the WP6 dataset was statistically similar to one another, and likewise for the methods in the cyclodextrin dataset. SMD and MM-PBSA/GBSA end-point calculations in the cyclodextrin challenge were an exception, where BFEs were estimated significantly too favorable by over 10 kcal mol^−1^. These differences in performance across host families are in keeping with prior SAMPL challenges, where often predictions for one host family seem easier than others, and this remained true here.

We find that the predictive accuracy of less computationally expensive models (machine learning and docking) was better or at least similar to more expensive and complex simulation based methods on the WP6 dataset. Using an ML model with molecular descriptors or an empirical approach from AutoDock Vina's smina scoring function for host–guest systems has a speed and perhaps cost effective advantage for BFE accuracy. ML based models have been among top performing methods (based on predictive accuracy) in recent SAMPL iterations, with an improvement in correlation metrics for Octa Acid hosts (from SAMPL7-8), but otherwise correlation metrics have been inconsistent in other host families (CB8 and WP6) and typically among the lowest. In contrast, simulation based approaches had a much better correlation to WP6 experimental measurements. Force field-based methods also show particularly severe errors for some chemistry, *e.g.* a sulfur-containing guest was among the worst performers and larger errors were obtained for a silicon-containing guest (discussed in Section 3.2). The combined strengths of these approaches – low errors in ML approaches but good correlation with force field based approaches – make a hybrid approach interesting. Some work in this direction has already been done; for example, some recent approaches have combined RBFEs (relative binding free energy calculations) and ML with success.^100–103^ Further work in this direction may thus be interesting for host–guest systems in SAMPL.

**Fig. 11:**
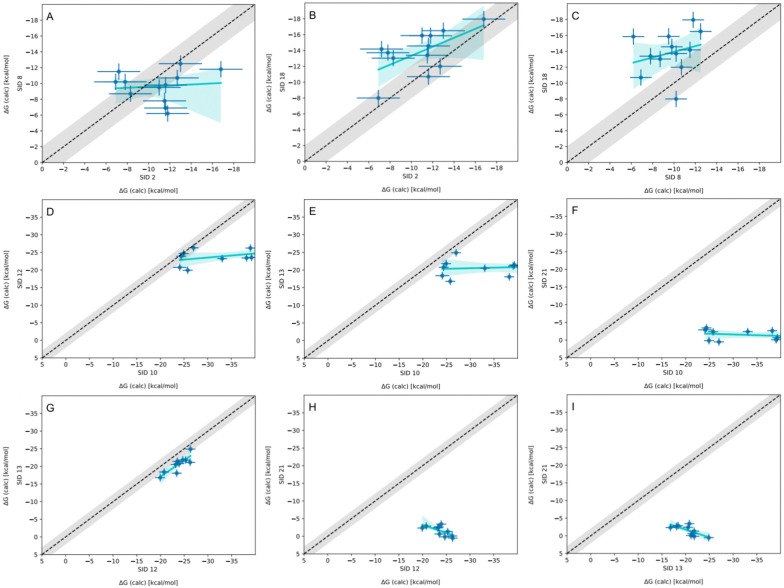
Correlation plots for methods using GAFF2.11 on WP6 or bCD datasets. Plots comparing methods using GAFF2.11 for predicting BFEs (kcal mol^−1^) in SAMPL9 host–guest challenge. The gray shaded region represents 2 kcal mol^−1^ error, the cyan solid line is a linear regression, and the cyan shaded region is the confidence interval. The submission IDs (SIDs) are shown in the respective *x* and *y* axes. (A)–(C) Compares methods using GAFF2.11 for WP6, and (D)–(I) are for bCD. The axis range and limit in plots D–I were adjusted to fit all points. Methods being compared in each plot are SIDs: (A) 2 and 8, (B) 2 and 18, (C) 8 and 18, (D) 10 and 12, (E) 10 and 13, (F) 10 and 21, (G) 12 and 13, (H) 12 and 21, and (I) 13 and 21.

Among physical methods, different methods using the same force field did not always agree with the predicted BFE value for the same systems. This was observed for methods using GAFF2.11 for both the WP6 and CD datasets and for methods using OpenFF2.0.0 for WP6 predictions. When we directly compared methods using GAFF2.11 (11), in the majority of cases at least half of the predicted BFEs differ by more than 2 kcal mol^−1^ (Fig. 11). The origins of the difference in results remain unclear.

In at least one case, the origin of the method differences is clear. In particular, several different free energy methods used OpenFF 2.0.0 and largely agreed with one another, except for three host–guest combinations (WP6-G1, WP6-G5, and WP6-G8; Fig. 8). In these specific cases, the methods – which should give equivalent results given the same model, setup, and enough simulation time – agree for all host–guest combinations except these three. For these three, they disagree, at least in part, because the participants selected different protonation states for the host and/or the guest for these specific complexes. Here, these discrepancies show the sensitivity of predicted BFEs to the chosen microstate(s) (specifically, protonation state) for the guest or host and help identify the likely dominant bound state(s). In addition, guests WP6-G1 and WP6-G8 have greater flexibility, thus differences or inadequate sampling of conformational space between the methods may have contributed, especially as the protonation states were varied.

From the SAMPL9 host–guest challenge, we learned several lessons related to system setup and guest binding modes.

One lesson is that careful system set-up is important, but not always straightforward even for systems that are thought to be simple. Part of the difficulty in this challenge was choosing microstate(s), and predicting and handling different potential binding poses when the binding mode(s) are not known. For example, there were questions on whether some of the WP6 carboxylate arms would remain protonated, thus reducing the WP6 formal charge from −12 to −10 or −8 or −6. The TFP guest had additional nitrogen at its alkyl amine side chain, adding protonation states that may need to be considered. In addition, some guests had induced chirality at nitrogen centers, and unique enantiomers that may not interconvert at simulation timescales and perhaps needed to be considered. In these cases, methods varied in how they modeled the relevant protonation states of guests and hosts at experimental pH, and binding modes considered for calculations.

We also learned important lessons about host binding modes in these systems. The use of multiple binding orientations, and/or microstates with the ATM approach had success in the SAMPL9 bCD dataset, and the method's previous success in SAMPL8 shows general applicability. The method thus has been among the most accurate across host categories. From the success of this method, we learned that multiple binding orientations in the primary and secondary face need to be considered (at least when binding mode(s) are unknown *a priori*) for most approaches; otherwise, relevant configurations would not be sampled at simulation time scales leading to inaccurate BFEs. However, results with the GROMOS 53*A*6_GLYC_ (DD/GROMOS-53A6_glyc/SPC/MD) force field had great agreement with the experimental binding free energy for PMT guest in complex with both bCD and HbCD, despite only considering one orientation, possibly due to this force field's more careful treatment of the hexopyranose based carbohydrates. These seemingly contradictory findings may indicate that systems with the PMT guest have a single and distinct dominant binding mode, whereas other cyclodextrin systems have a different binding mode and perhaps multiple equally dominant modes.

Despite the overall success of the method using the AMOEBA force field in SAMPL9, modeling more accurate electrostatic potentials around WP6 host–guest molecules did not provide additional accuracy (statistically) compared to methods using fixed point-charge force fields. This observation tells us that there are likely other issues which were more accuracy-limiting for this dataset.

In general, accurately ranking host–guest systems was rather difficult for most methods, even for methods that had the best error metrics. Methods had greater success at predicting the tightest binding guest for the WP6 host. Accurately predicting weaker binders remains a challenge for almost all methods as has been the case in previous SAMPL iterations. Interestingly, no method correctly predicted the tightest or weakest binders for bCD or HbCD hosts. Given that the dynamic range of experimental measurements (from strongest to weakest) for the bCD-phenothiazine dataset was less than 2 kcal mol^−1^, greater BFE accuracy would be needed to accurately rank the complexes.

For some host–guest systems, nearly equivalent methods gave significantly different BFEs, which remains rather puzzling. It is likely that such BFE discrepancies originate from differences in system set-up and/or sampling of relevant configuration space. Not surprisingly, methods which differed by multiple simulation parameters, despite using the same energy model, also gave different BFEs. In comparing methods with multiple differences, we can't draw any conclusions. Overall, then, further work is needed to ensure that differences in host–guest binding predictions can be traced to their source.

Despite progress in modeling host–guest binding interactions, these systems remain a challenging test case for binding free energy calculations, and capture some of the same challenges as protein–ligand binding free energy calculations. Thus, they are likely to remain a helpful test case for the field for some time to come.

## 5 Code and data

https://github.com/samplchallenges/SAMPL9/tree/main/host_guest. An archive copy of the SAMPL9 GitHub repository host–guest challenge directory is also available in the Supplementary Documents bundle (SAMPL9-supplementary-documents.tar.gz). Some useful files from this repository are highlighted below.

• Table of participants' submission filenames and their submission ID: https://github.com/samplchallenges/SAMPL9/tree/main/host_guest/Analysis/SAMPL9-user-map-HG.csv

• Submission files of prediction sets: https://github.com/samplchallenges/SAMPL9/tree/main/host_guest/Analysis/Submissions

• Python analysis scripts: https://github.com/samplchallenges/SAMPL9/tree/main/host_guest/Analysis/Scripts

• Table of performance statistics calculated for ranked methods for the WP6 dataset: https://github.com/samplchallenges/SAMPL9/tree/main/host_guest/Analysis/Ranked_Accuracy/WP6/StatisticsTables/statistics.csv

• Table of performance statistics calculated for all methods for the WP6 dataset: https://github.com/samplchallenges/SAMPL9/tree/main/host_guest/Analysis/All_Accuracy/WP6/StatisticsTables/statistics.csv

• Table of performance statistics calculated for ranked methods for the CD dataset: https://github.com/samplchallenges/SAMPL9/tree/main/host_guest/Analysis/Ranked_Accuracy/CD/StatisticsTables/statistics.csv

• Table of performance statistics calculated for all methods for the CD dataset: https://github.com/samplchallenges/SAMPL9/tree/main/host_guest/Analysis/All_Accuracy/CD/StatisticsTables/statistics.csv

• Table of performance statistics calculated for ranked methods for WP6 (without optionals) dataset: https://github.com/samplchallenges/SAMPL9/tree/main/host_guest/Analysis/Ranked_Accuracy/WP6_no_optional/StatisticsTables/statistics.csv

• Table of performance statistics calculated for all methods for WP6 (without optionals) dataset: https://github.com/samplchallenges/SAMPL9/tree/main/host_guest/Analysis/All_Accuracy/WP6_no_optional/StatisticsTables/statistics.csv

## Author contributions

The manuscript was written through the contributions of all authors. All authors have approved the final version of the manuscript.

## Conflicts of interest

DLM is a member of the Scientific Advisory Boards of OpenEye Scientific Software, Cadence Molecular Sciences and Anagenex and an Open Science Fellow with Psivant Therapeutics.

## Supplementary Material

CP-026-D3CP05111K-s001

CP-026-D3CP05111K-s002
