# Supplementary figures and images for: The SAMPL9 host–guest blind challenge: an overview of binding free energy predictive accuracy (part 2 of 2)
Source: Phys Chem Chem Phys. 2024 Mar 4;26(12):9207–25. doi: 10.1039/d3cp05111k (PMC10954238; doi:10.1039/d3cp05111k)

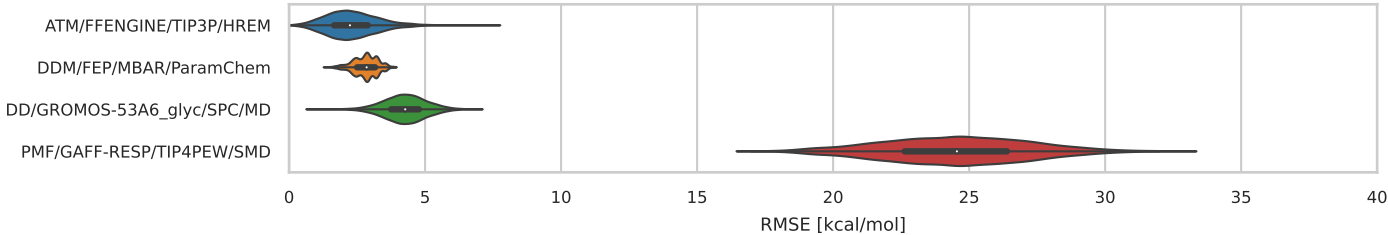

Supplement: CP-026-D3CP05111K-s001 [file CP-026-D3CP05111K-s001.zip › sampl9-supplementary-documents/Users/amezcum1/Desktop/SAMPL9/host_guest/Analysis/Ranked_Accuracy/bCD/StatisticsPlots/RMSE_bootstrap_distributions.pdf]

PMF/GAFF-RESP/TIP4PEW/SMD - CD (10)

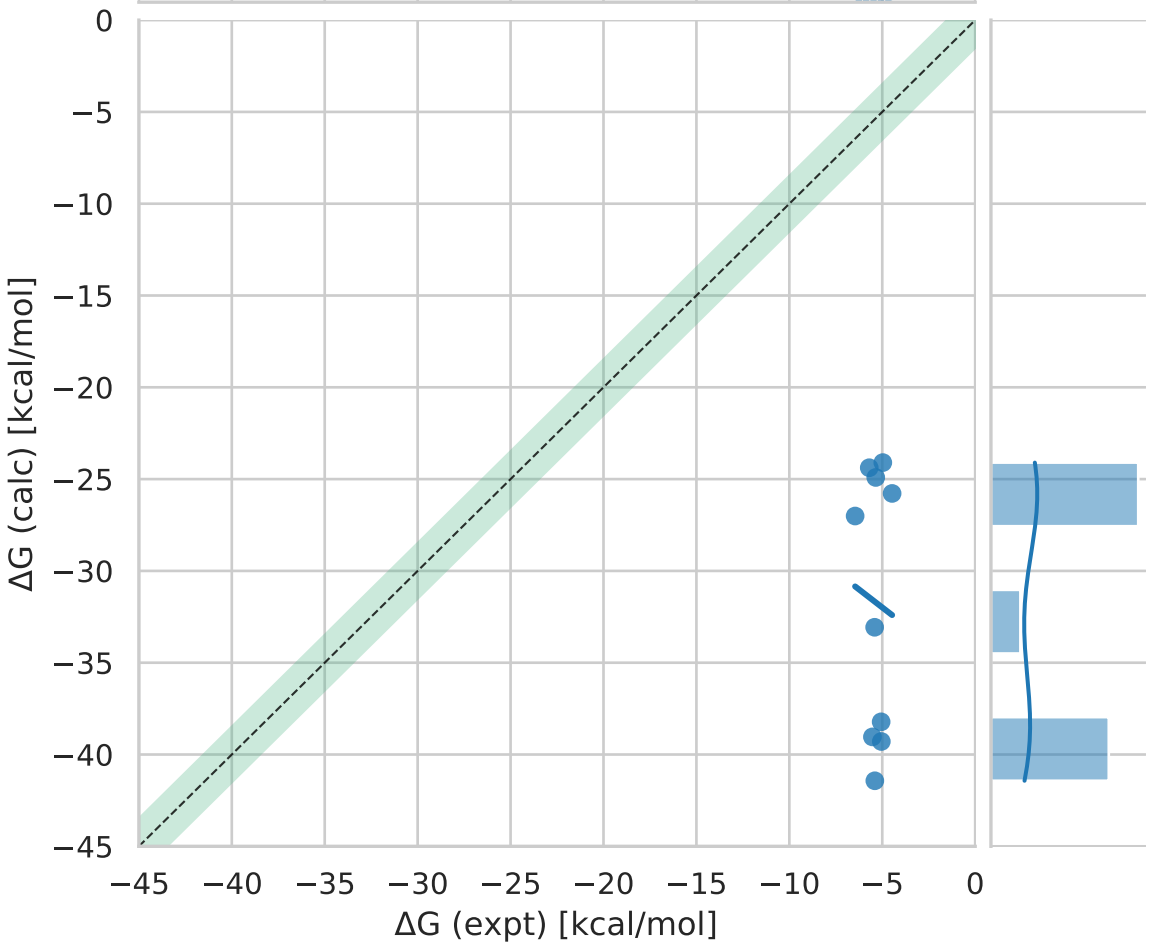

Supplement: CP-026-D3CP05111K-s001 [file CP-026-D3CP05111K-s001.zip › sampl9-supplementary-documents/Users/amezcum1/Desktop/SAMPL9/host_guest/Analysis/Ranked_Accuracy/CD/FreeEnergyCorrelationPlots/10.pdf]

DD/GROMOS-53A6\_glyc/SPC/MD - CD (11)

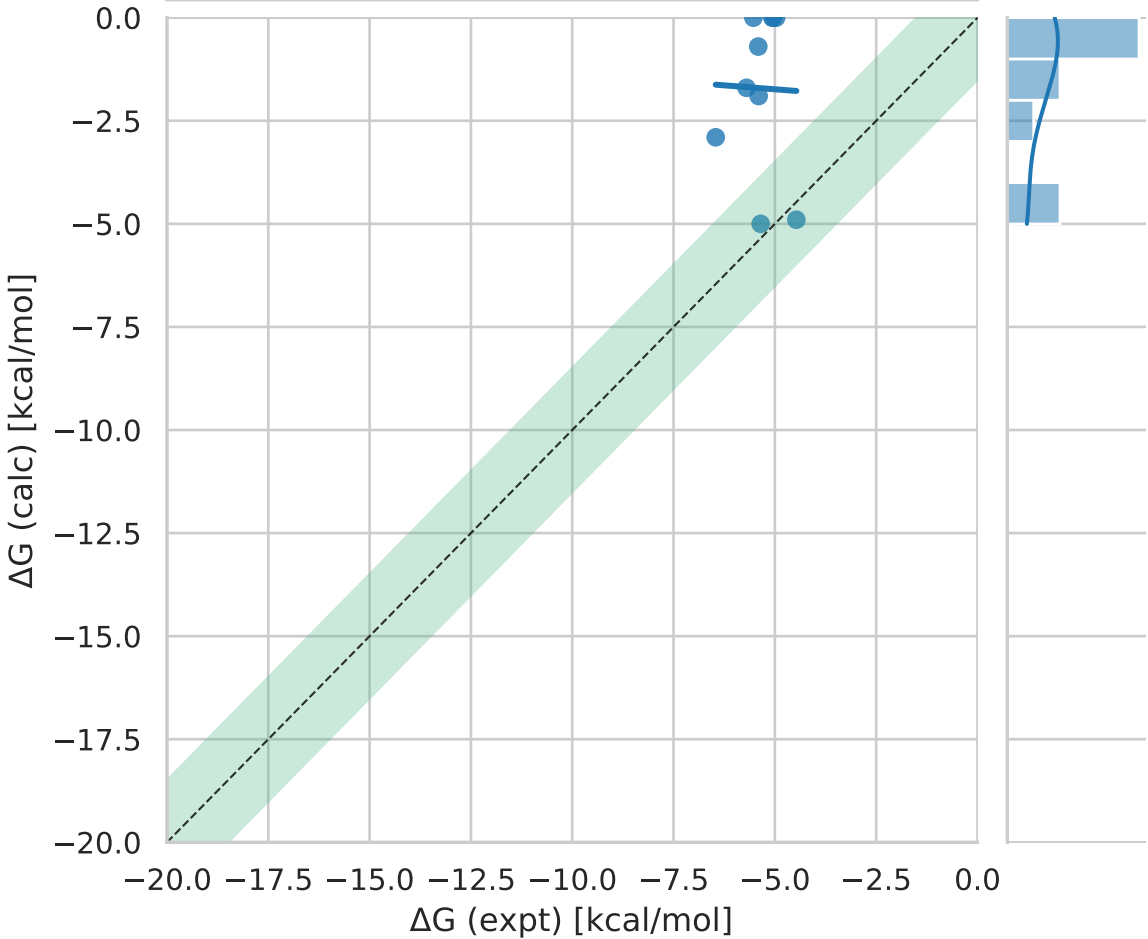

Supplement: CP-026-D3CP05111K-s001 [file CP-026-D3CP05111K-s001.zip › sampl9-supplementary-documents/Users/amezcum1/Desktop/SAMPL9/host_guest/Analysis/Ranked_Accuracy/CD/FreeEnergyCorrelationPlots/11.pdf]

# ATM/FFENGINE/TIP3P/HREM - CD (14)

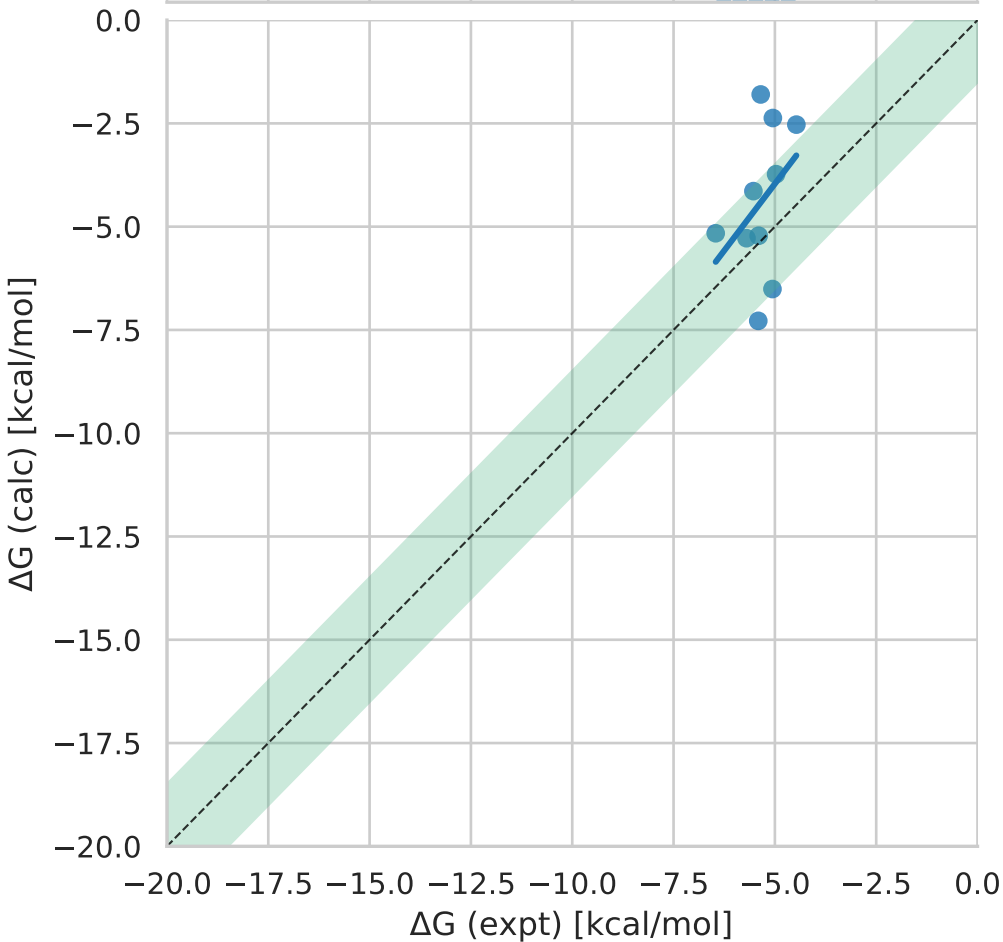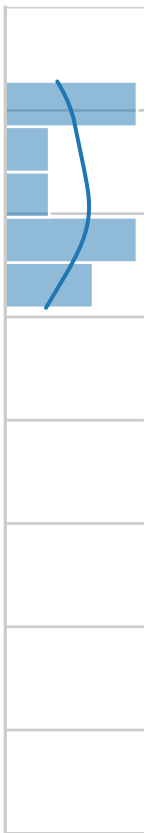

Supplement: CP-026-D3CP05111K-s001 [file CP-026-D3CP05111K-s001.zip › sampl9-supplementary-documents/Users/amezcum1/Desktop/SAMPL9/host_guest/Analysis/Ranked_Accuracy/CD/FreeEnergyCorrelationPlots/14.pdf]

DDM/FEP/MBAR/ParamChem - CD (16)

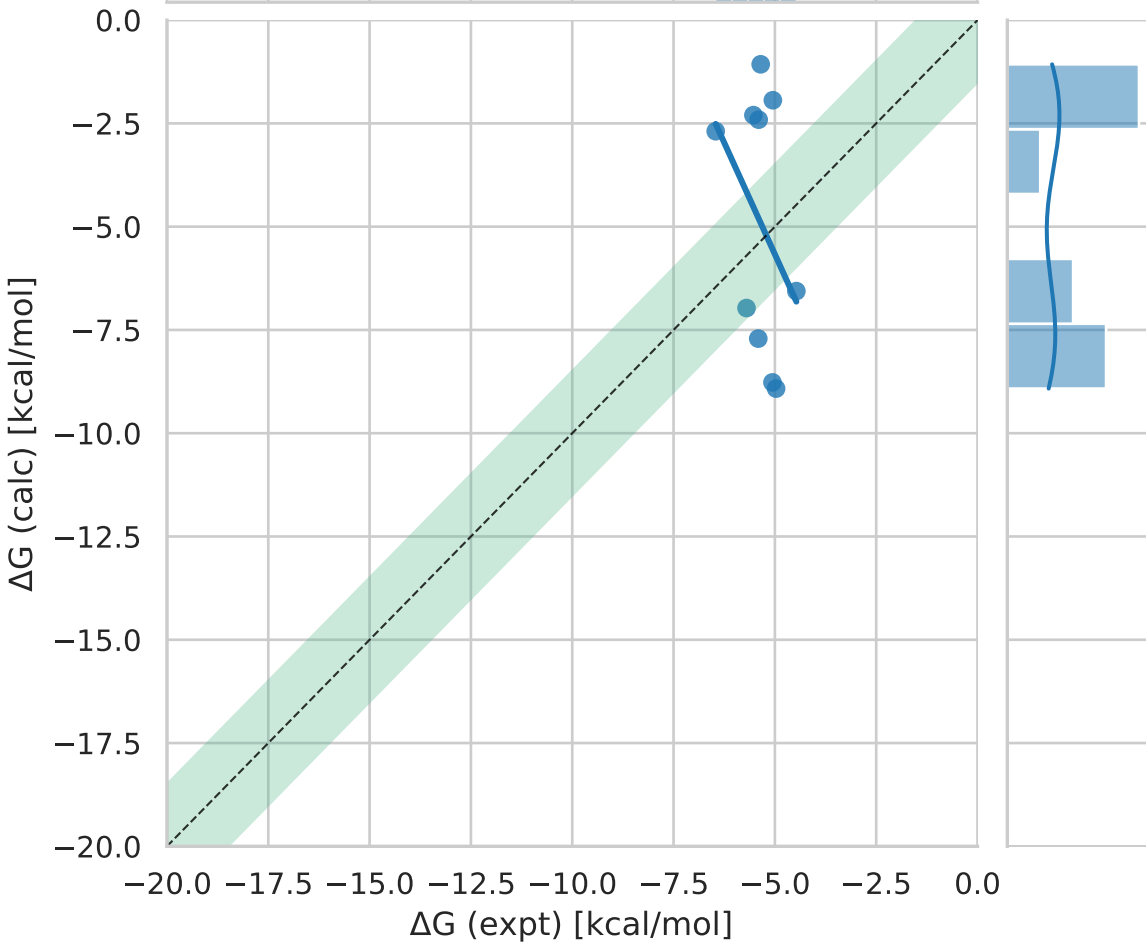

Supplement: CP-026-D3CP05111K-s001 [file CP-026-D3CP05111K-s001.zip › sampl9-supplementary-documents/Users/amezcum1/Desktop/SAMPL9/host_guest/Analysis/Ranked_Accuracy/CD/FreeEnergyCorrelationPlots/16.pdf]

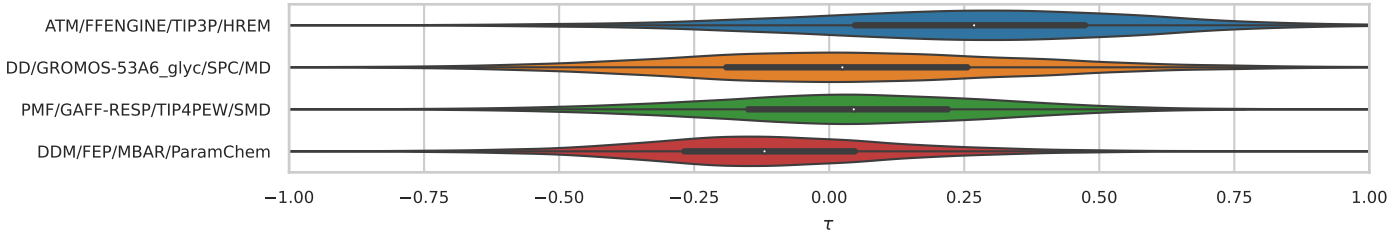

Supplement: CP-026-D3CP05111K-s001 [file CP-026-D3CP05111K-s001.zip › sampl9-supplementary-documents/Users/amezcum1/Desktop/SAMPL9/host_guest/Analysis/Ranked_Accuracy/CD/StatisticsPlots/kendall_tau_bootstrap_distributions.pdf]

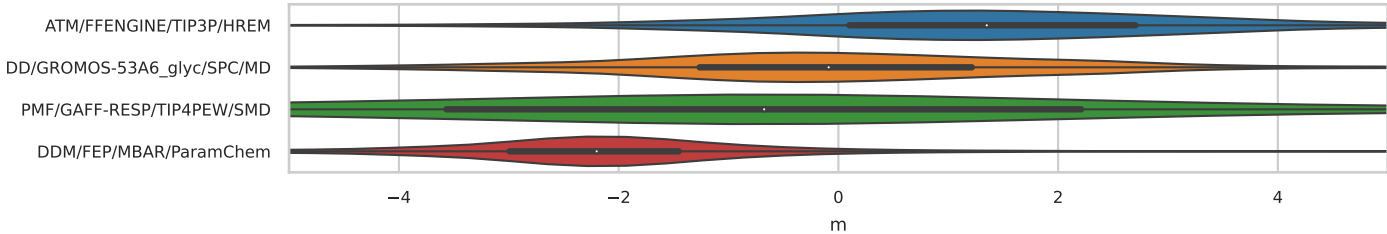

Supplement: CP-026-D3CP05111K-s001 [file CP-026-D3CP05111K-s001.zip › sampl9-supplementary-documents/Users/amezcum1/Desktop/SAMPL9/host_guest/Analysis/Ranked_Accuracy/CD/StatisticsPlots/m_bootstrap_distributions.pdf]

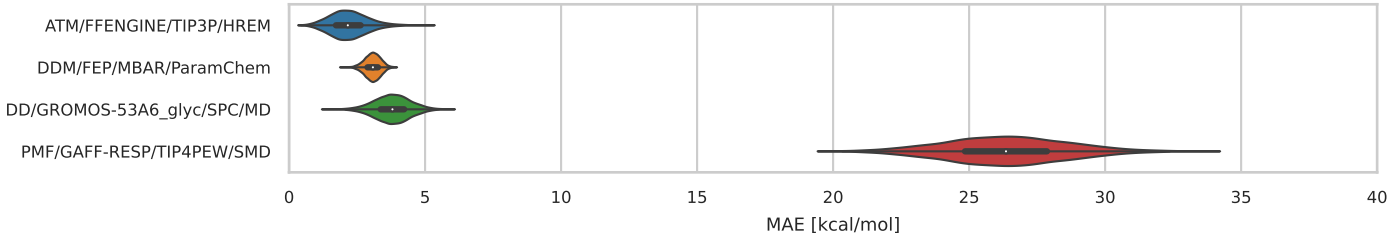

Supplement: CP-026-D3CP05111K-s001 [file CP-026-D3CP05111K-s001.zip › sampl9-supplementary-documents/Users/amezcum1/Desktop/SAMPL9/host_guest/Analysis/Ranked_Accuracy/CD/StatisticsPlots/MAE_bootstrap_distributions.pdf]

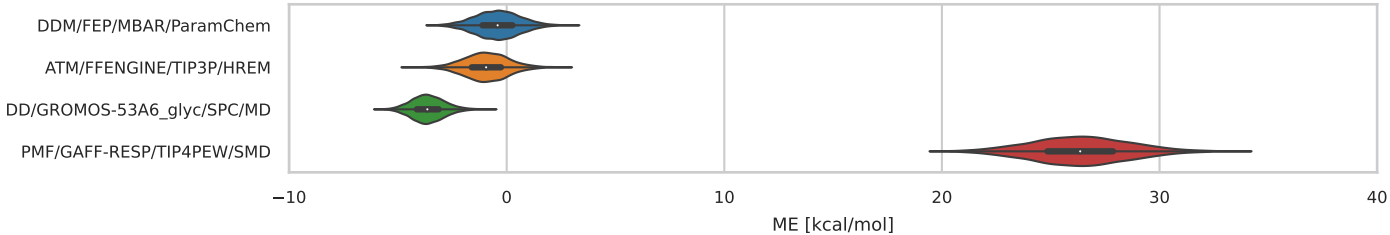

Supplement: CP-026-D3CP05111K-s001 [file CP-026-D3CP05111K-s001.zip › sampl9-supplementary-documents/Users/amezcum1/Desktop/SAMPL9/host_guest/Analysis/Ranked_Accuracy/CD/StatisticsPlots/ME_bootstrap_distributions.pdf]

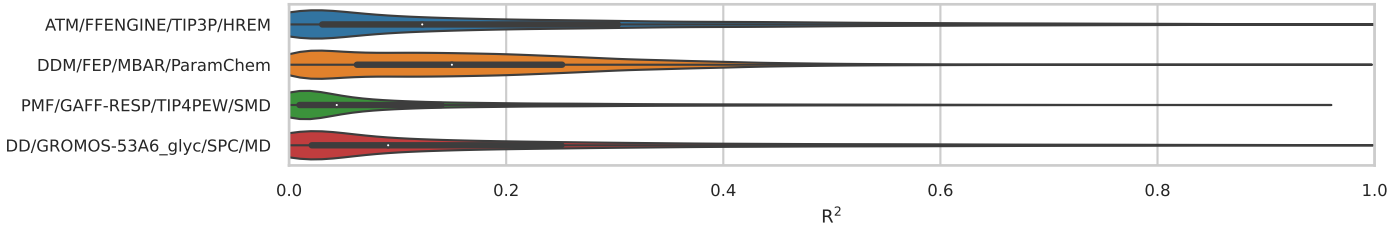

Supplement: CP-026-D3CP05111K-s001 [file CP-026-D3CP05111K-s001.zip › sampl9-supplementary-documents/Users/amezcum1/Desktop/SAMPL9/host_guest/Analysis/Ranked_Accuracy/CD/StatisticsPlots/R2_bootstrap_distributions.pdf]

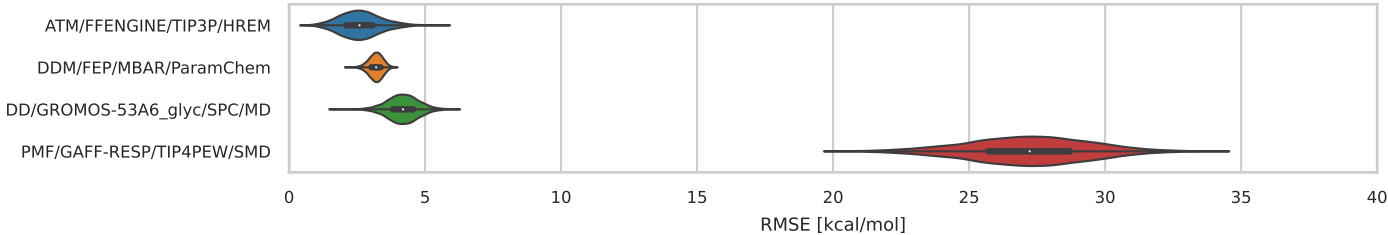

Supplement: CP-026-D3CP05111K-s001 [file CP-026-D3CP05111K-s001.zip › sampl9-supplementary-documents/Users/amezcum1/Desktop/SAMPL9/host_guest/Analysis/Ranked_Accuracy/CD/StatisticsPlots/RMSE_bootstrap_distributions.pdf]

# PMF/GAFF-RESP/TIP4PEW/SMD - HbCD (10)

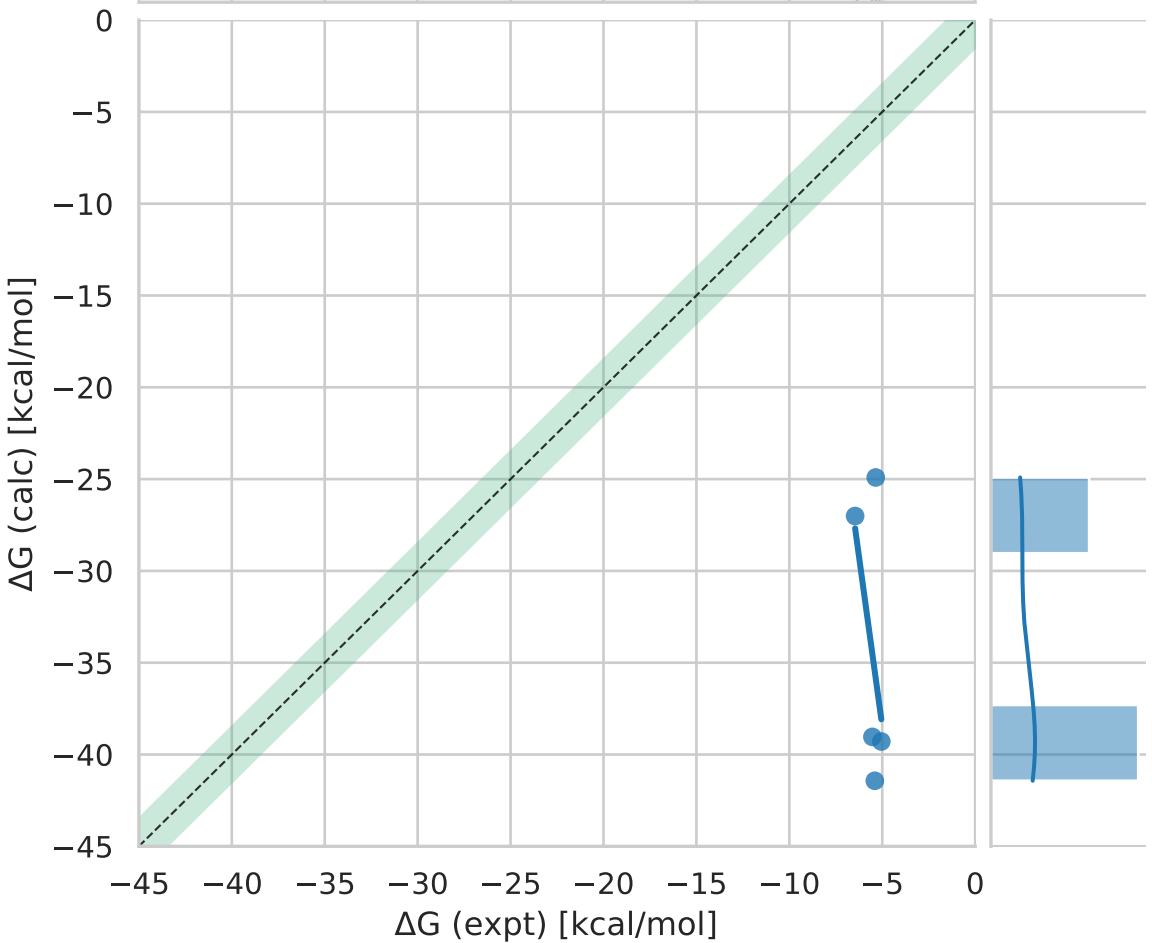

Supplement: CP-026-D3CP05111K-s001 [file CP-026-D3CP05111K-s001.zip › sampl9-supplementary-documents/Users/amezcum1/Desktop/SAMPL9/host_guest/Analysis/Ranked_Accuracy/HbCD/FreeEnergyCorrelationPlots/10.pdf]

DD/GROMOS-53A6\_glyc/SPC/MD - HbCD (11)

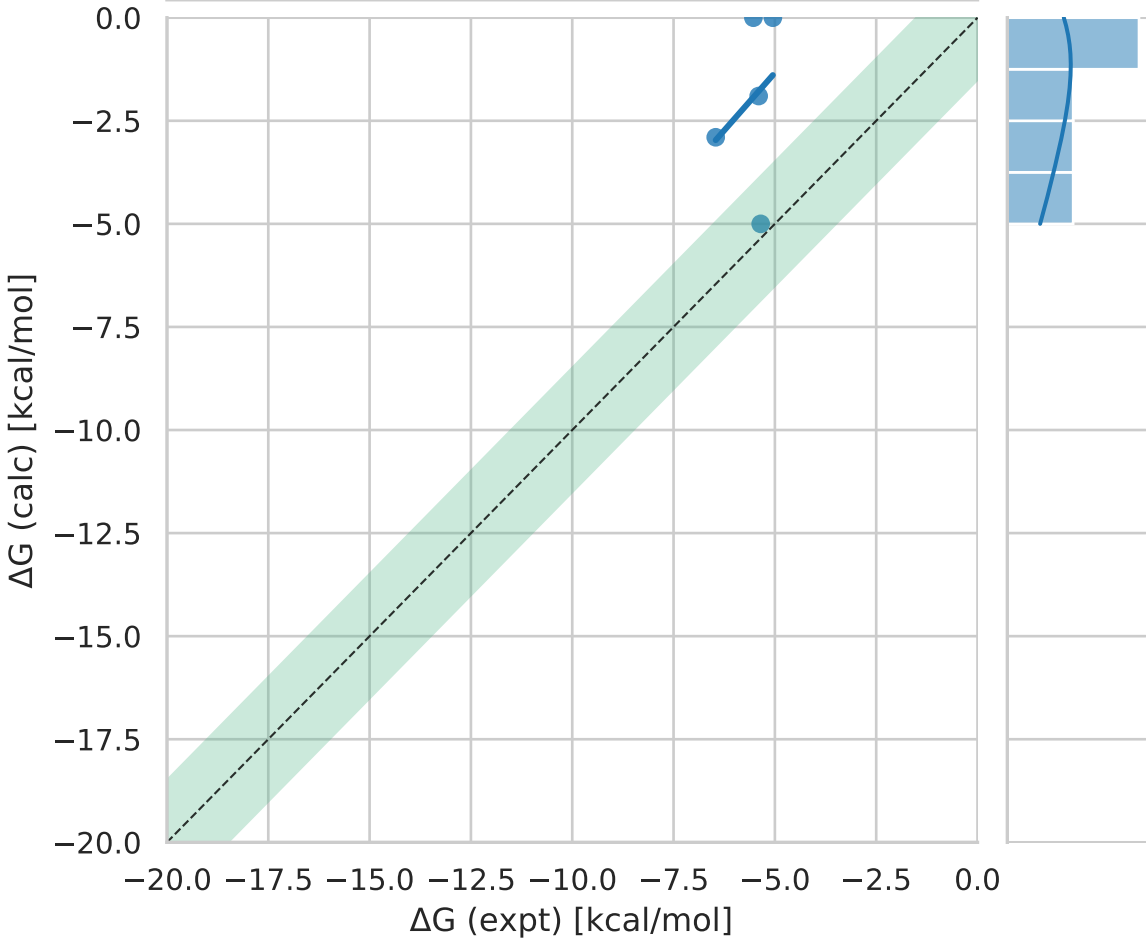

Supplement: CP-026-D3CP05111K-s001 [file CP-026-D3CP05111K-s001.zip › sampl9-supplementary-documents/Users/amezcum1/Desktop/SAMPL9/host_guest/Analysis/Ranked_Accuracy/HbCD/FreeEnergyCorrelationPlots/11.pdf]

# ATM/FFENGINE/TIP3P/HREM - HbCD (14)

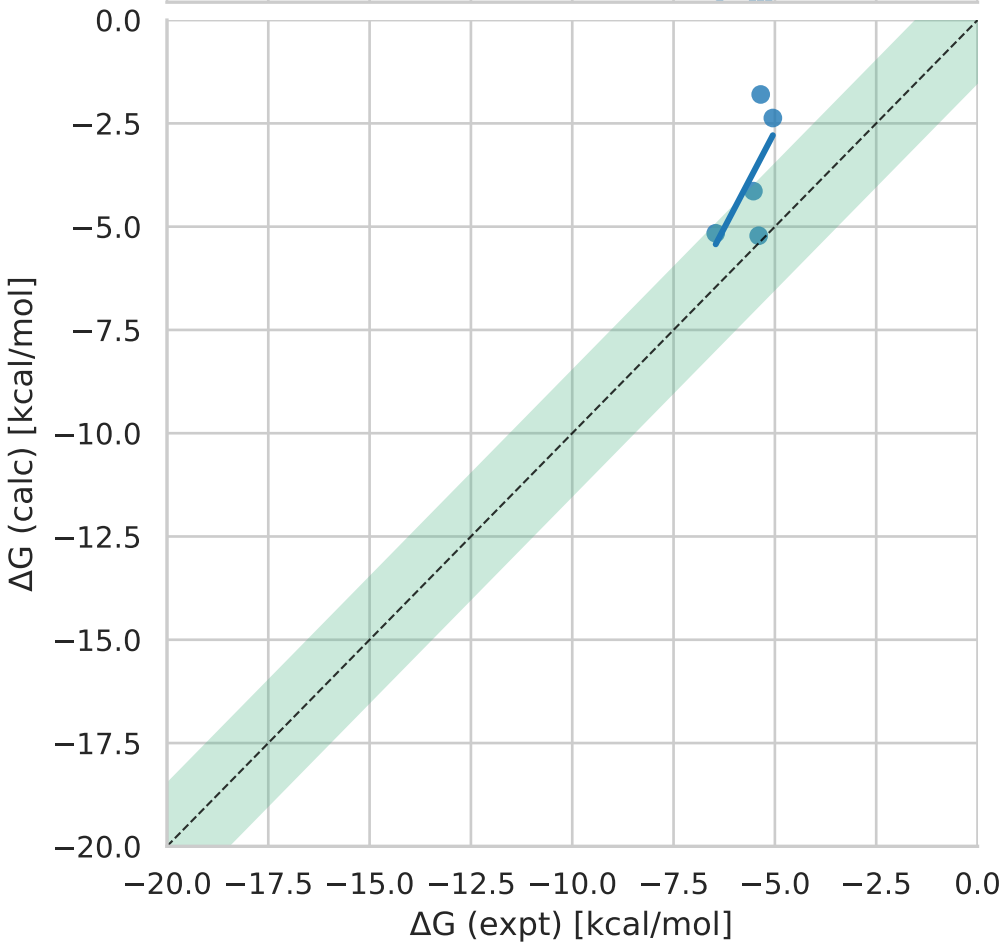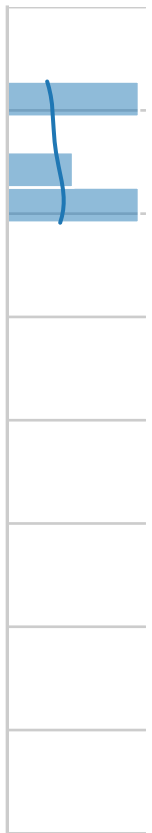

Supplement: CP-026-D3CP05111K-s001 [file CP-026-D3CP05111K-s001.zip › sampl9-supplementary-documents/Users/amezcum1/Desktop/SAMPL9/host_guest/Analysis/Ranked_Accuracy/HbCD/FreeEnergyCorrelationPlots/14.pdf]

DDM/FEP/MBAR/ParamChem - HbCD (16)

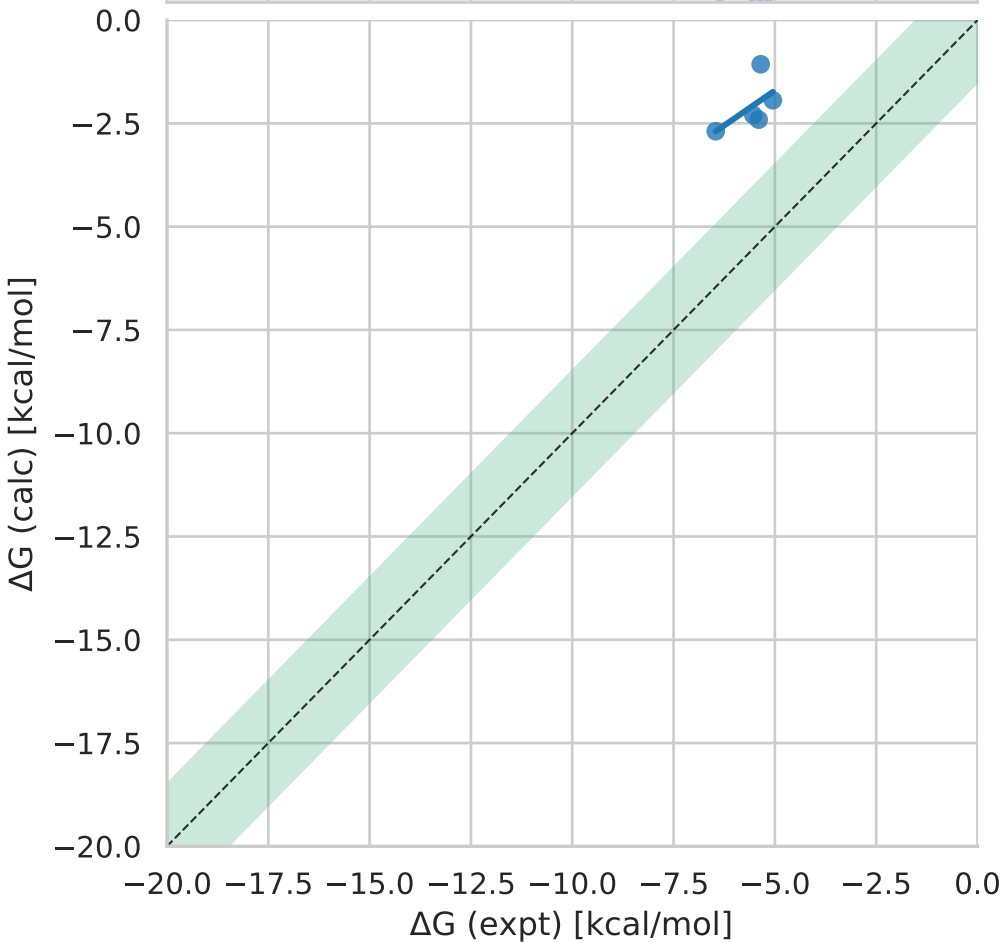

Supplement: CP-026-D3CP05111K-s001 [file CP-026-D3CP05111K-s001.zip › sampl9-supplementary-documents/Users/amezcum1/Desktop/SAMPL9/host_guest/Analysis/Ranked_Accuracy/HbCD/FreeEnergyCorrelationPlots/16.pdf]

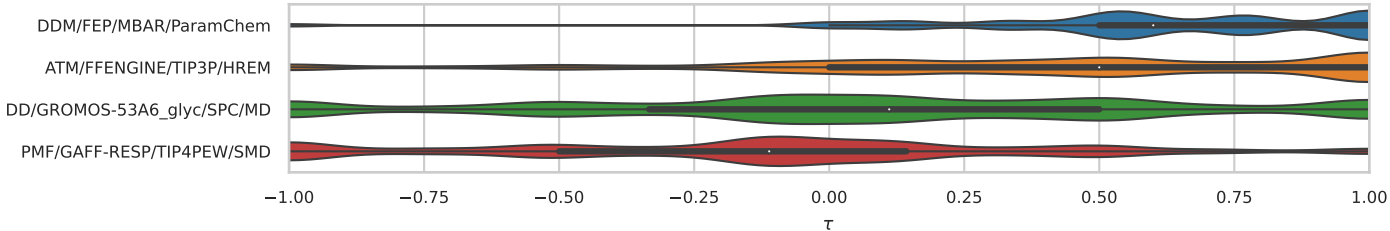

Supplement: CP-026-D3CP05111K-s001 [file CP-026-D3CP05111K-s001.zip › sampl9-supplementary-documents/Users/amezcum1/Desktop/SAMPL9/host_guest/Analysis/Ranked_Accuracy/HbCD/StatisticsPlots/kendall_tau_bootstrap_distributions.pdf]

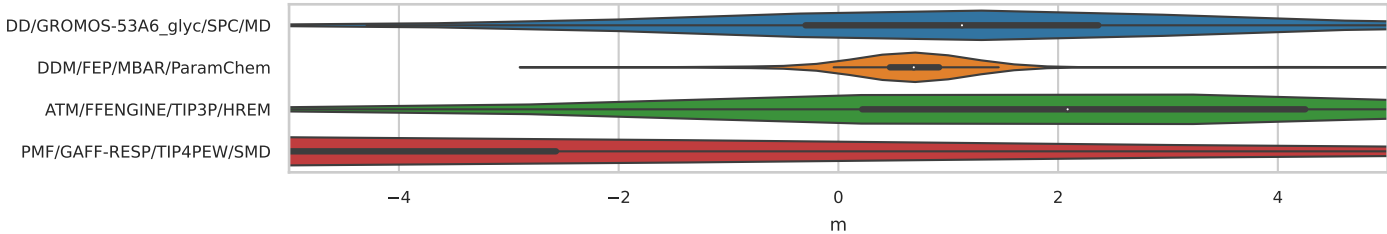

Supplement: CP-026-D3CP05111K-s001 [file CP-026-D3CP05111K-s001.zip › sampl9-supplementary-documents/Users/amezcum1/Desktop/SAMPL9/host_guest/Analysis/Ranked_Accuracy/HbCD/StatisticsPlots/m_bootstrap_distributions.pdf]

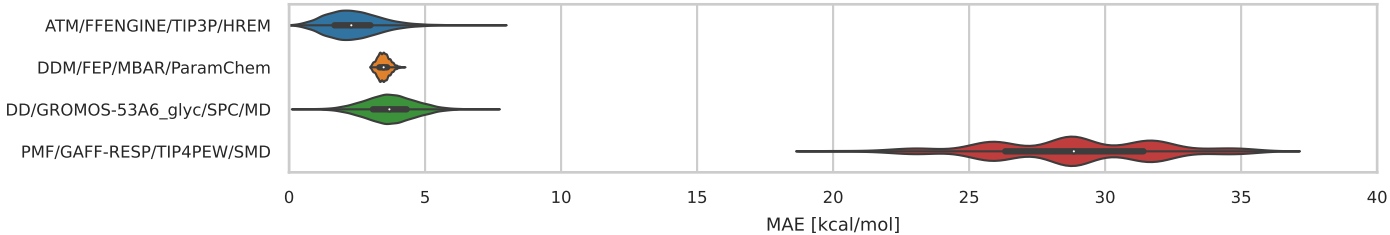

Supplement: CP-026-D3CP05111K-s001 [file CP-026-D3CP05111K-s001.zip › sampl9-supplementary-documents/Users/amezcum1/Desktop/SAMPL9/host_guest/Analysis/Ranked_Accuracy/HbCD/StatisticsPlots/MAE_bootstrap_distributions.pdf]

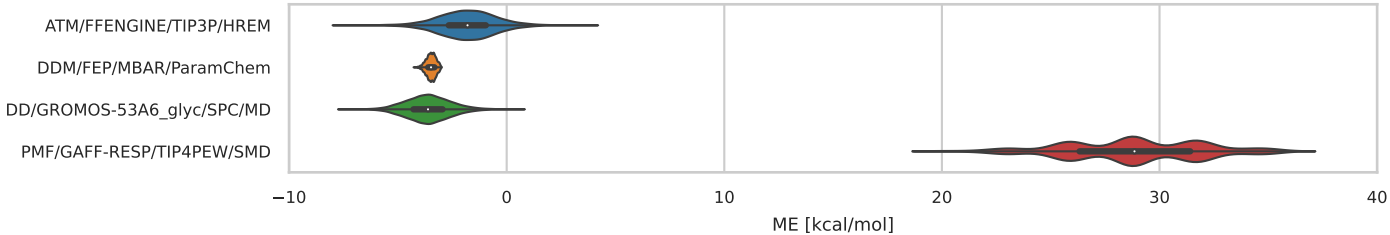

Supplement: CP-026-D3CP05111K-s001 [file CP-026-D3CP05111K-s001.zip › sampl9-supplementary-documents/Users/amezcum1/Desktop/SAMPL9/host_guest/Analysis/Ranked_Accuracy/HbCD/StatisticsPlots/ME_bootstrap_distributions.pdf]

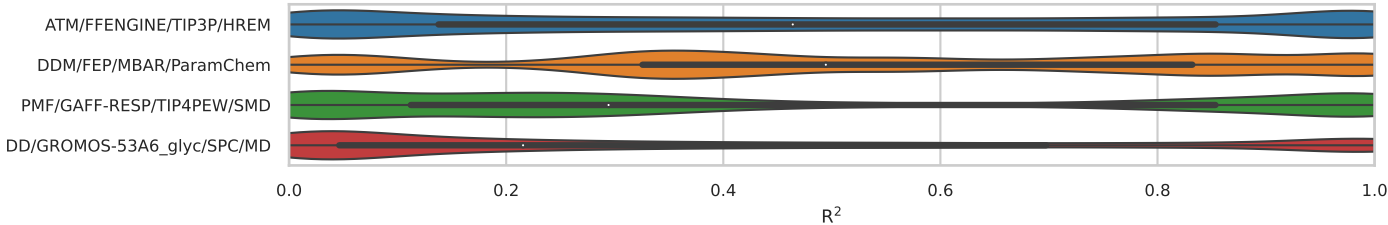

Supplement: CP-026-D3CP05111K-s001 [file CP-026-D3CP05111K-s001.zip › sampl9-supplementary-documents/Users/amezcum1/Desktop/SAMPL9/host_guest/Analysis/Ranked_Accuracy/HbCD/StatisticsPlots/R2_bootstrap_distributions.pdf]

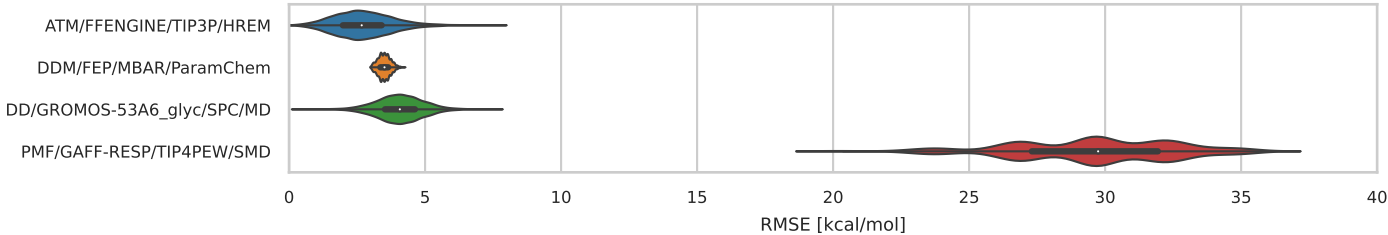

Supplement: CP-026-D3CP05111K-s001 [file CP-026-D3CP05111K-s001.zip › sampl9-supplementary-documents/Users/amezcum1/Desktop/SAMPL9/host_guest/Analysis/Ranked_Accuracy/HbCD/StatisticsPlots/RMSE_bootstrap_distributions.pdf]

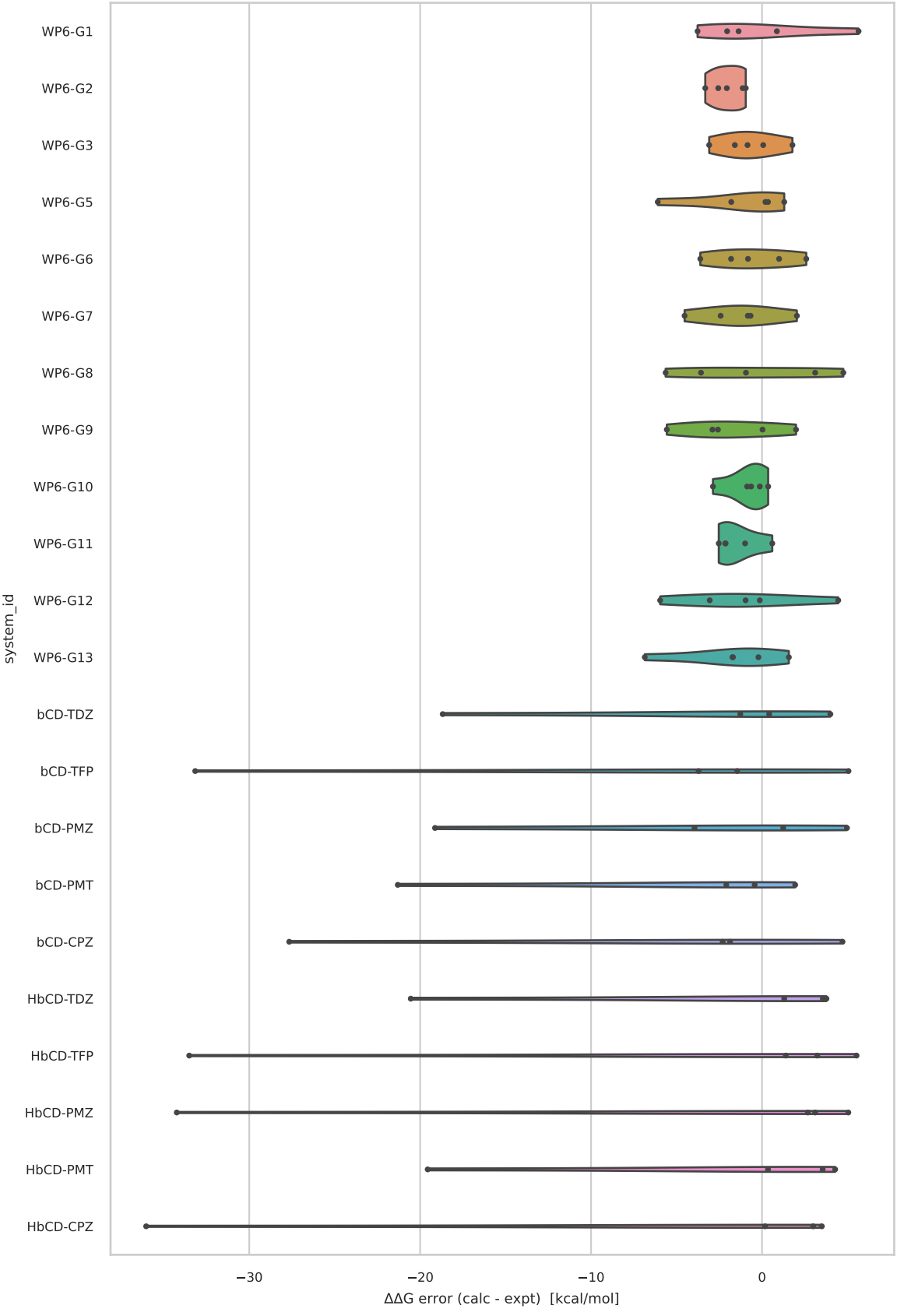

Supplement: CP-026-D3CP05111K-s001 [file CP-026-D3CP05111K-s001.zip › sampl9-supplementary-documents/Users/amezcum1/Desktop/SAMPL9/host_guest/Analysis/Ranked_Accuracy/MoleculesStatistics/molecules_error.pdf]

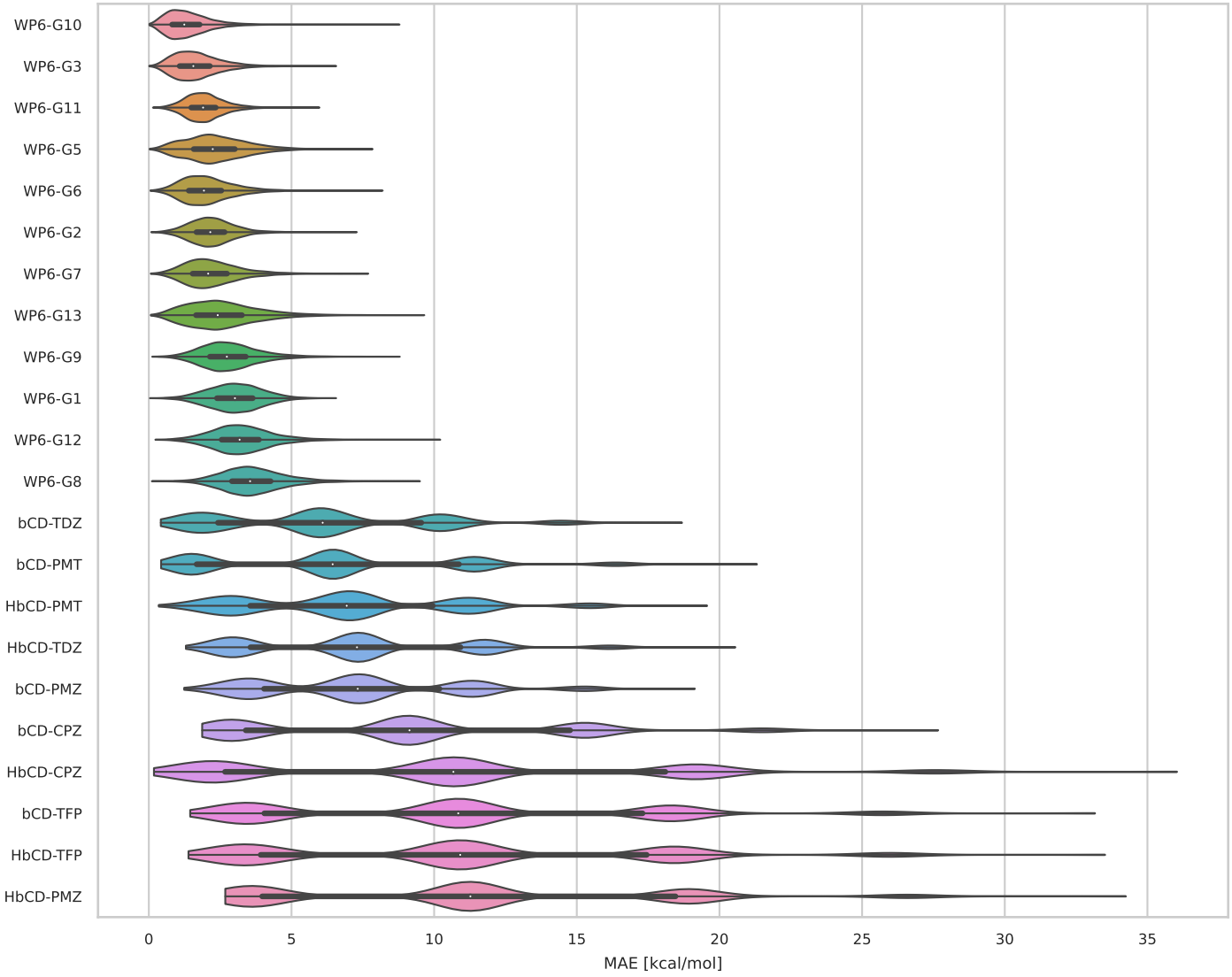

Supplement: CP-026-D3CP05111K-s001 [file CP-026-D3CP05111K-s001.zip › sampl9-supplementary-documents/Users/amezcum1/Desktop/SAMPL9/host_guest/Analysis/Ranked_Accuracy/MoleculesStatistics/StatisticsPlots/MAE_bootstrap_distributions.pdf]

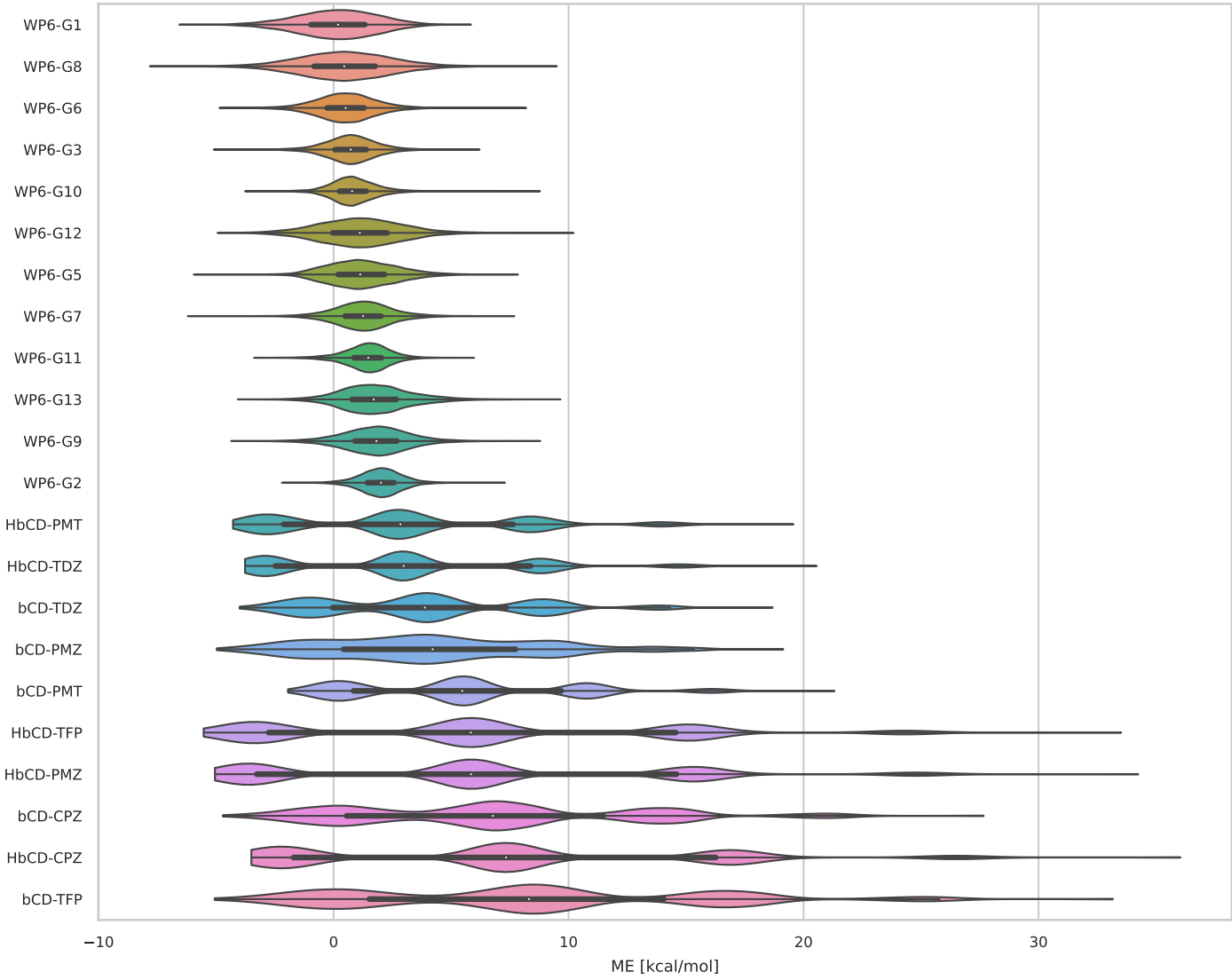

Supplement: CP-026-D3CP05111K-s001 [file CP-026-D3CP05111K-s001.zip › sampl9-supplementary-documents/Users/amezcum1/Desktop/SAMPL9/host_guest/Analysis/Ranked_Accuracy/MoleculesStatistics/StatisticsPlots/ME_bootstrap_distributions.pdf]

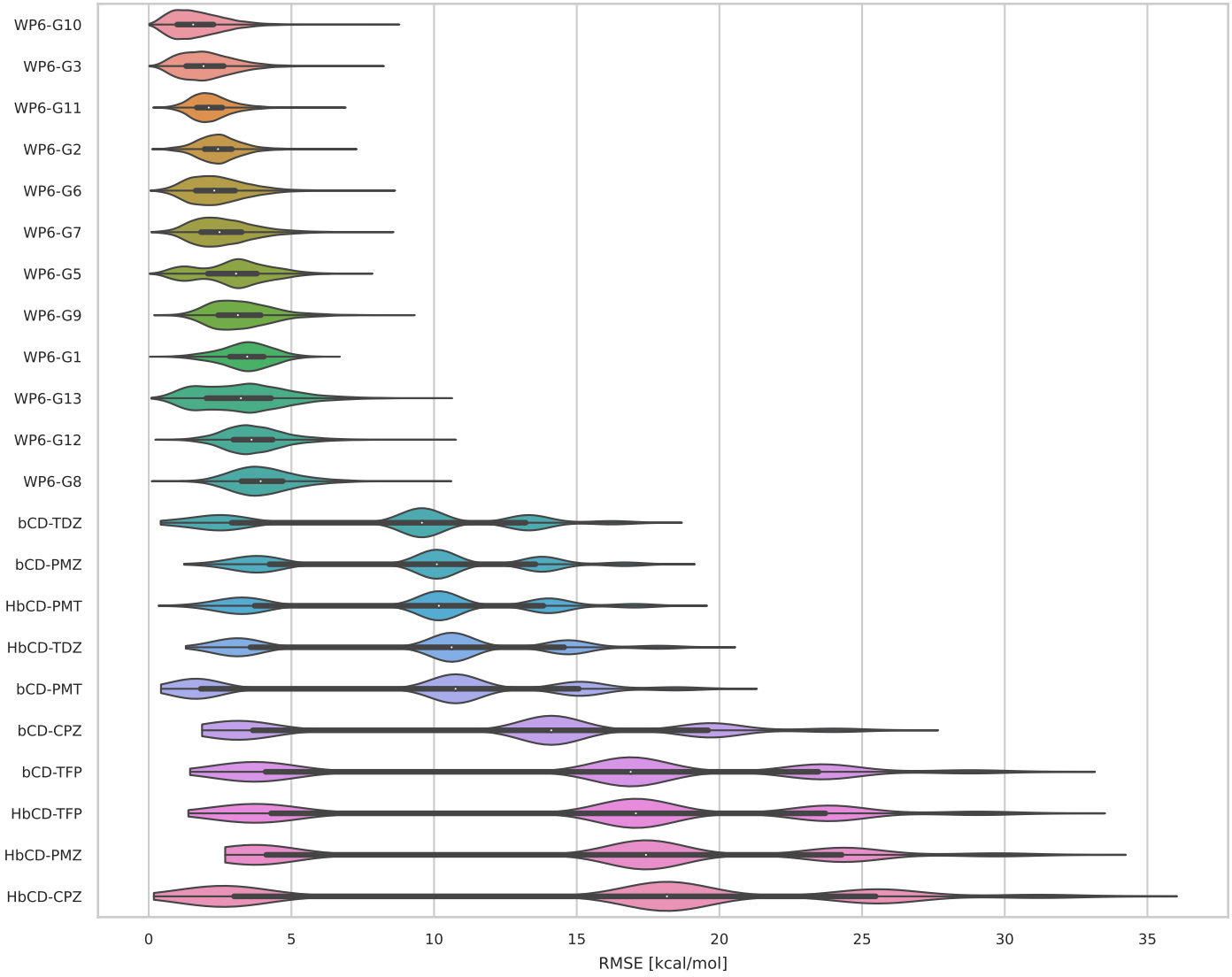

Supplement: CP-026-D3CP05111K-s001 [file CP-026-D3CP05111K-s001.zip › sampl9-supplementary-documents/Users/amezcum1/Desktop/SAMPL9/host_guest/Analysis/Ranked_Accuracy/MoleculesStatistics/StatisticsPlots/RMSE_bootstrap_distributions.pdf]

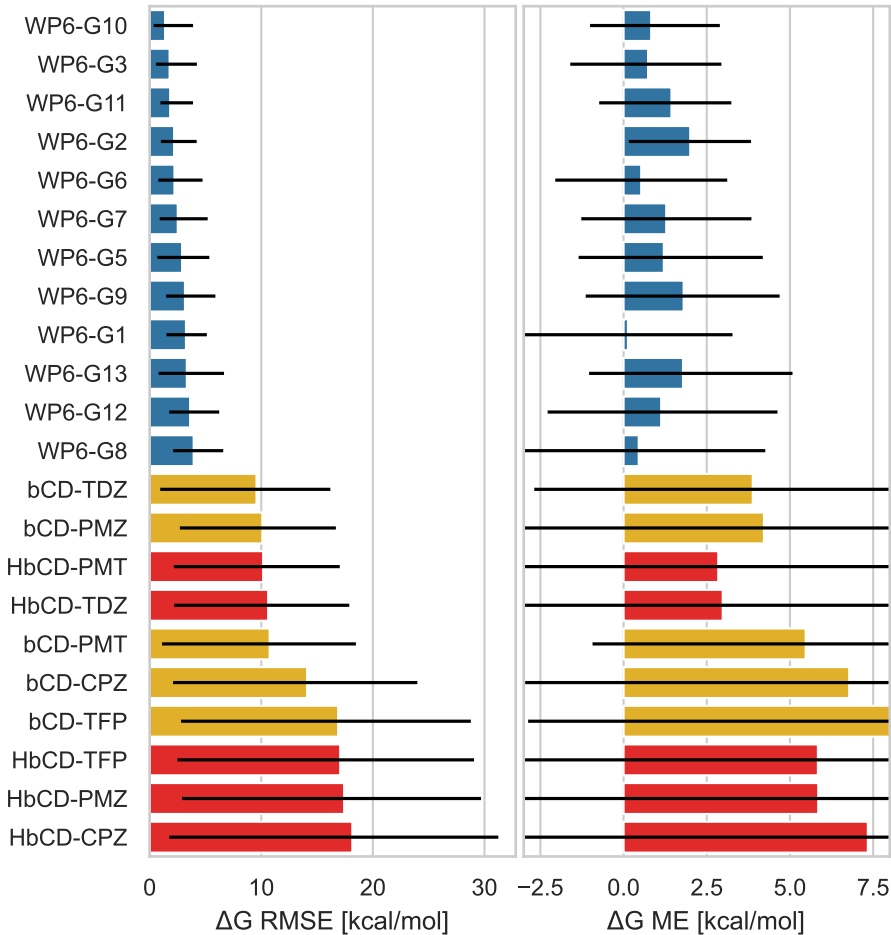

Supplement: CP-026-D3CP05111K-s001 [file CP-026-D3CP05111K-s001.zip › sampl9-supplementary-documents/Users/amezcum1/Desktop/SAMPL9/host_guest/Analysis/Ranked_Accuracy/PaperImages/error_by_molecule.pdf]

$\Delta G$  (calc) [kcal/mol]

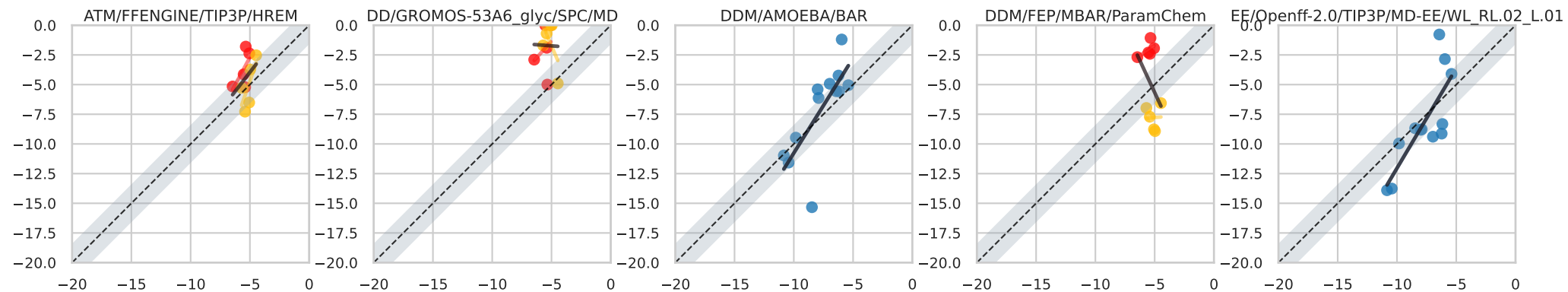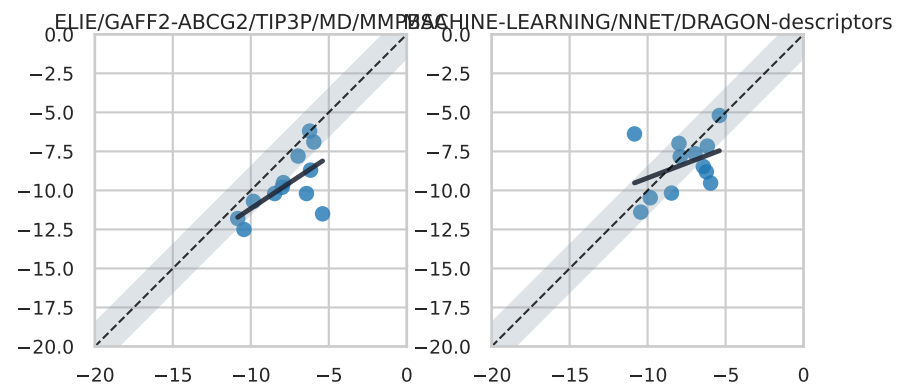

$\Delta G$  (exp) [kcal/mol]

Supplement: CP-026-D3CP05111K-s001 [file CP-026-D3CP05111K-s001.zip › sampl9-supplementary-documents/Users/amezcum1/Desktop/SAMPL9/host_guest/Analysis/Ranked_Accuracy/PaperImages/Figure_correlation_plots_ranked_methods.pdf]

System ID

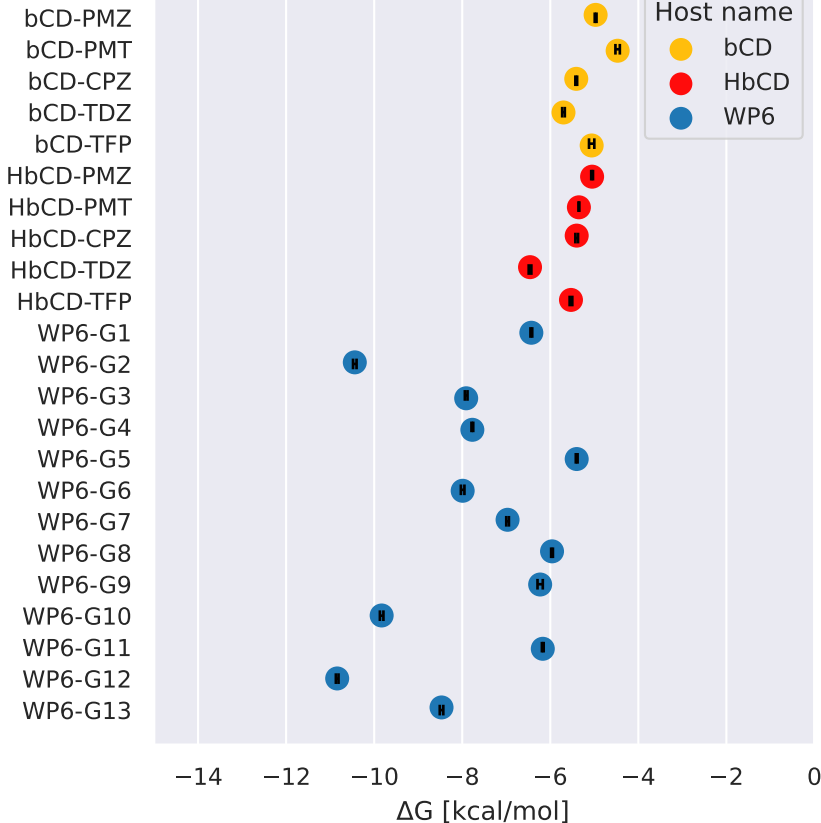

Supplement: CP-026-D3CP05111K-s001 [file CP-026-D3CP05111K-s001.zip › sampl9-supplementary-documents/Users/amezcum1/Desktop/SAMPL9/host_guest/Analysis/Ranked_Accuracy/PaperImages/Figure2_experimental_measurements.pdf]

# MACHINE-LEARNING/NNET/DRAGON-descriptors - WP6 (3)

$\Delta H$  (calc) [kcal/mol]

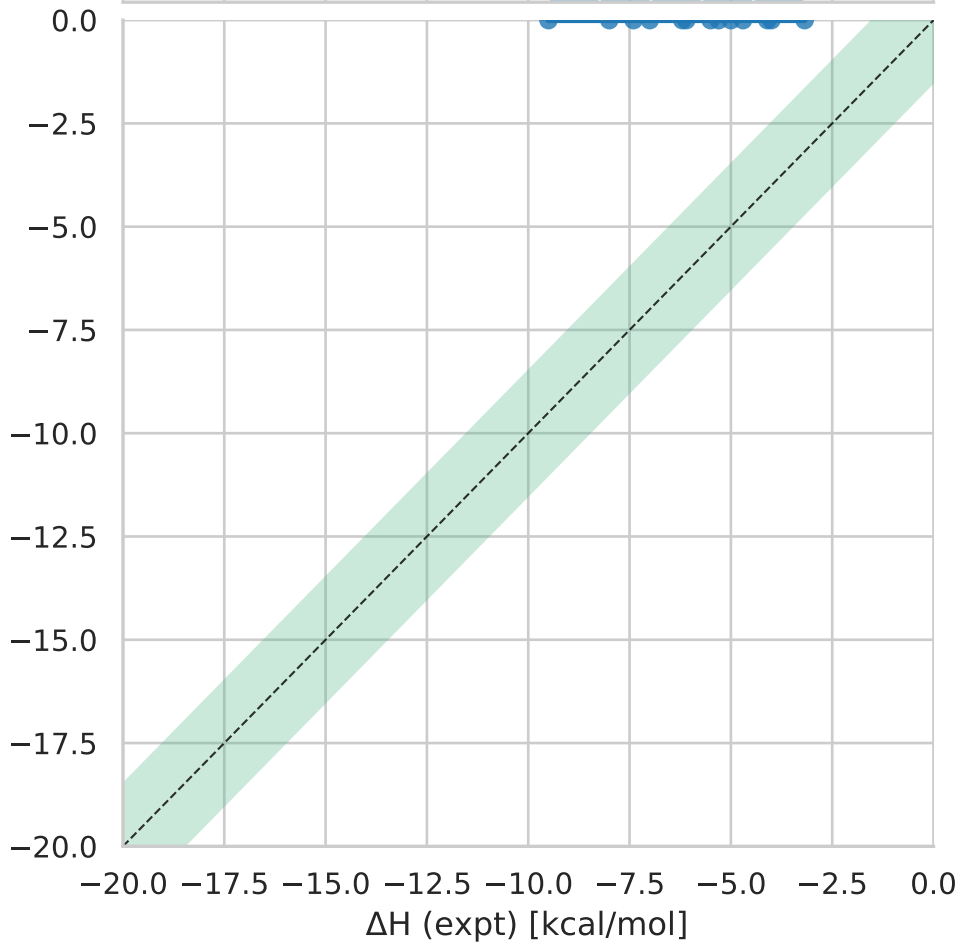

Supplement: CP-026-D3CP05111K-s001 [file CP-026-D3CP05111K-s001.zip › sampl9-supplementary-documents/Users/amezcum1/Desktop/SAMPL9/host_guest/Analysis/Ranked_Accuracy/WP6/EnthalpiesCorrelationPlots/3.pdf]

# vDSSB/GAFF2/OPC3/HREM - WP6 (2)

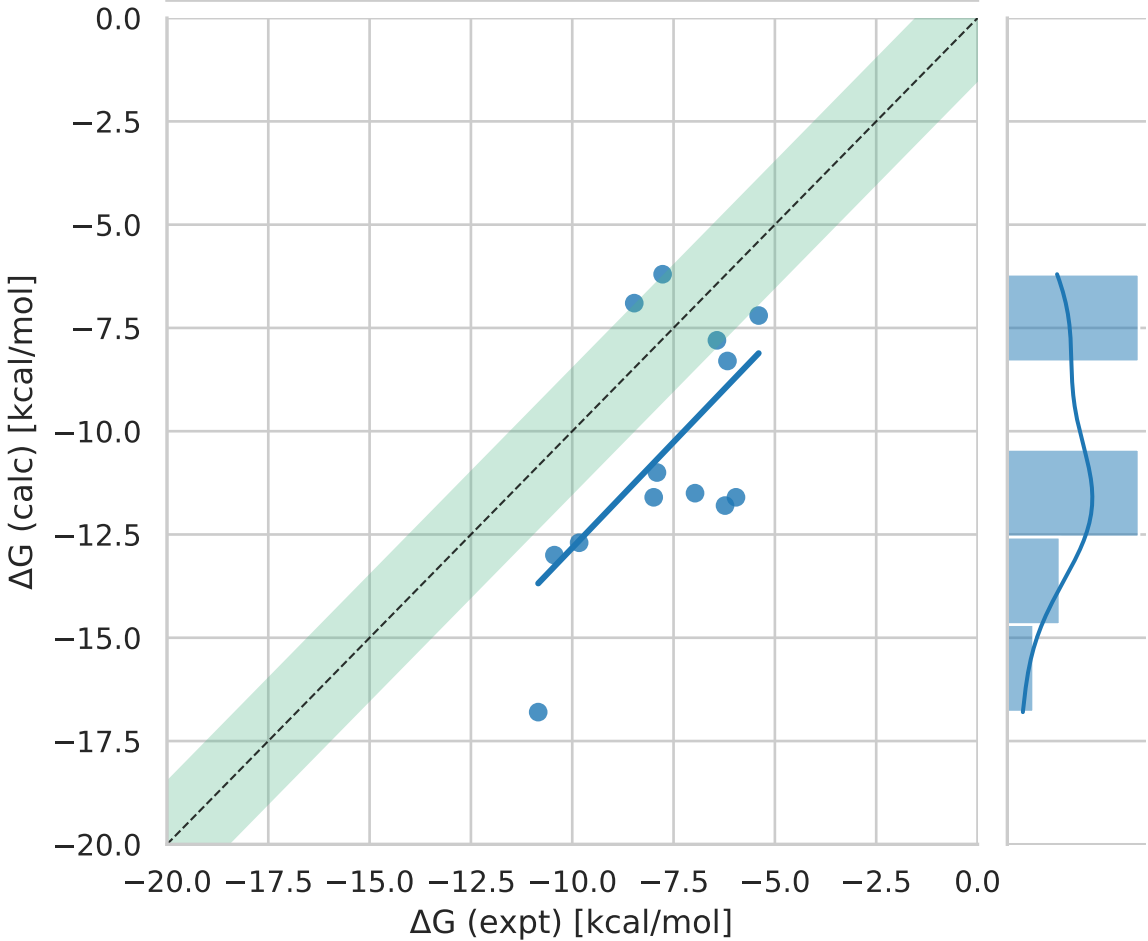

Supplement: CP-026-D3CP05111K-s001 [file CP-026-D3CP05111K-s001.zip › sampl9-supplementary-documents/Users/amezcum1/Desktop/SAMPL9/host_guest/Analysis/Ranked_Accuracy/WP6/FreeEnergyCorrelationPlots/2.pdf]

# MACHINE-LEARNING/NNET/DRAGON-descriptors - WP6 (3)

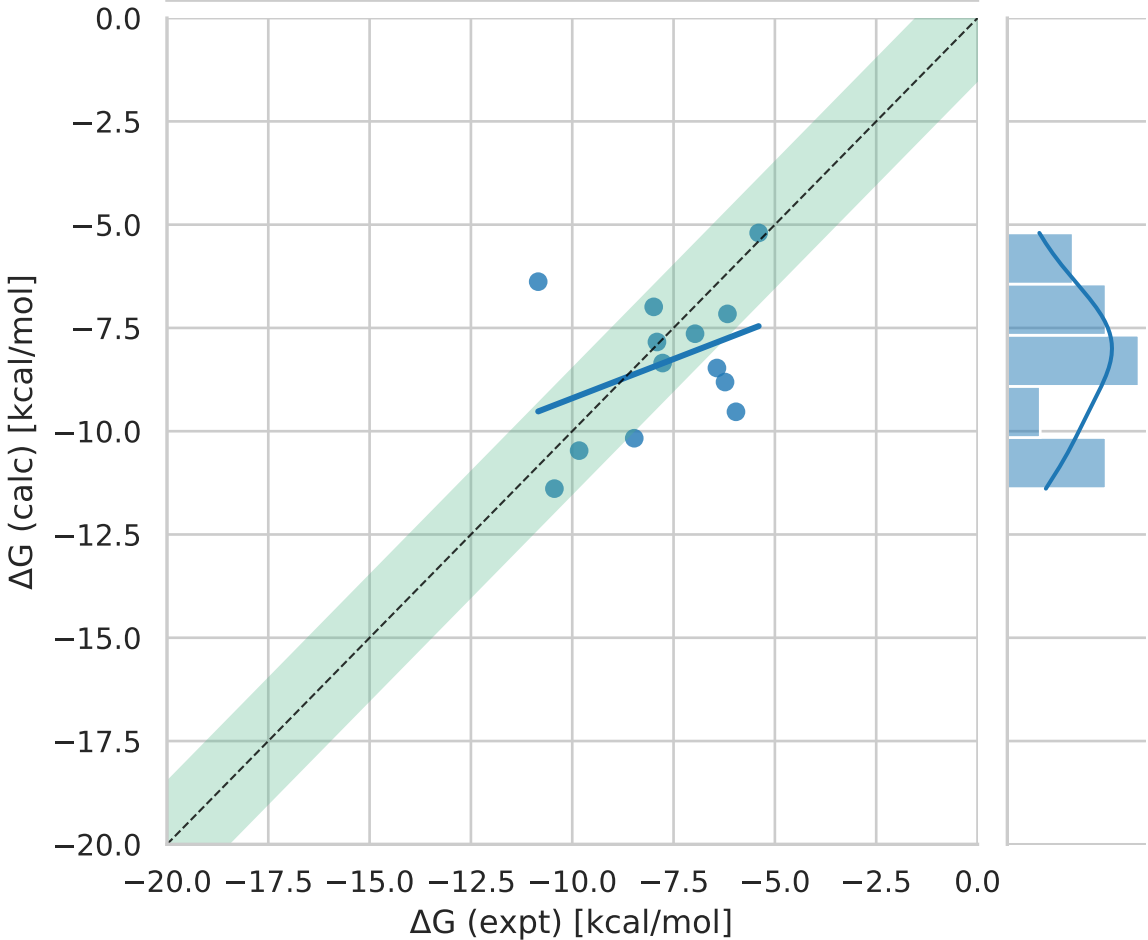

Supplement: CP-026-D3CP05111K-s001 [file CP-026-D3CP05111K-s001.zip › sampl9-supplementary-documents/Users/amezcum1/Desktop/SAMPL9/host_guest/Analysis/Ranked_Accuracy/WP6/FreeEnergyCorrelationPlots/3.pdf]

EE/Openff-2.0/TIP3P/MD-EE/WL\_RL.02\_L.01 - WP6 (4)

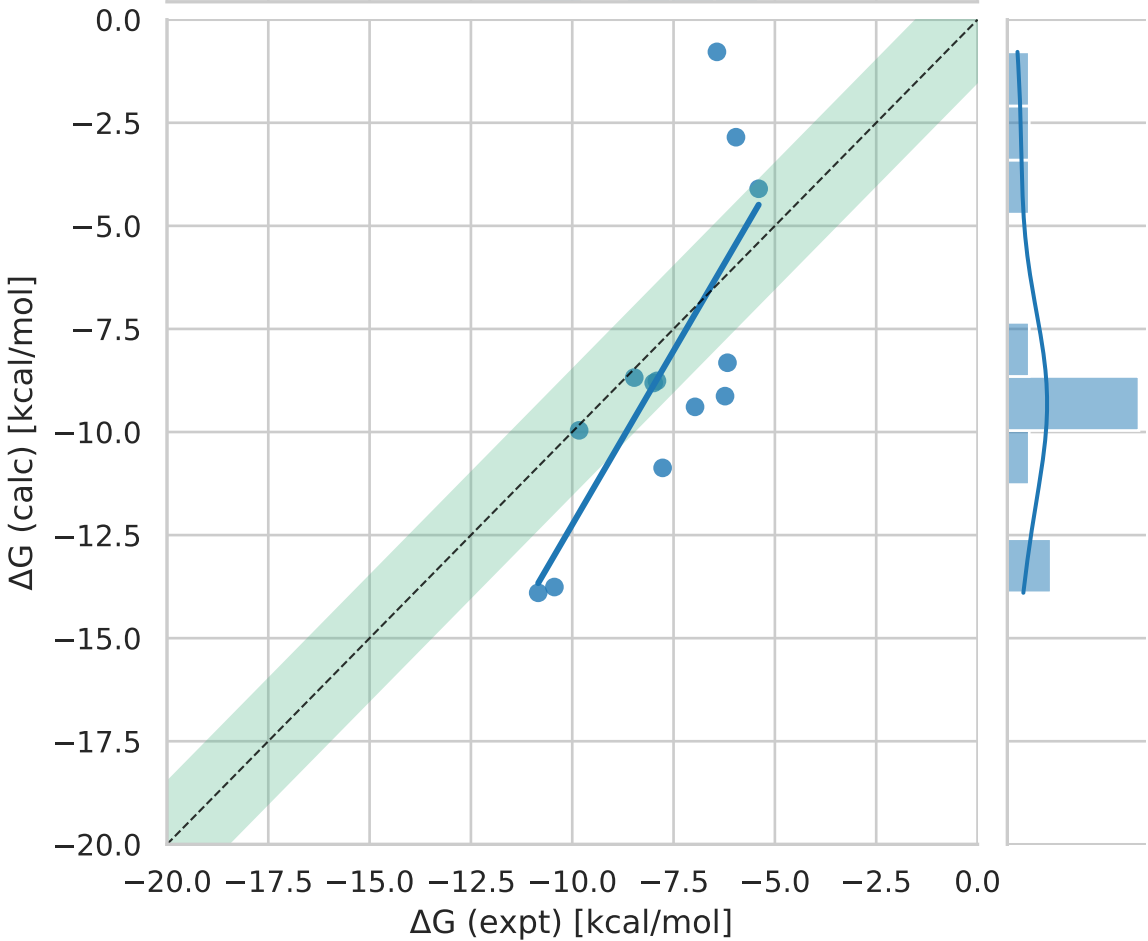

Supplement: CP-026-D3CP05111K-s001 [file CP-026-D3CP05111K-s001.zip › sampl9-supplementary-documents/Users/amezcum1/Desktop/SAMPL9/host_guest/Analysis/Ranked_Accuracy/WP6/FreeEnergyCorrelationPlots/4.pdf]

DDM/AMOEBA/BAR - WP6 (7)

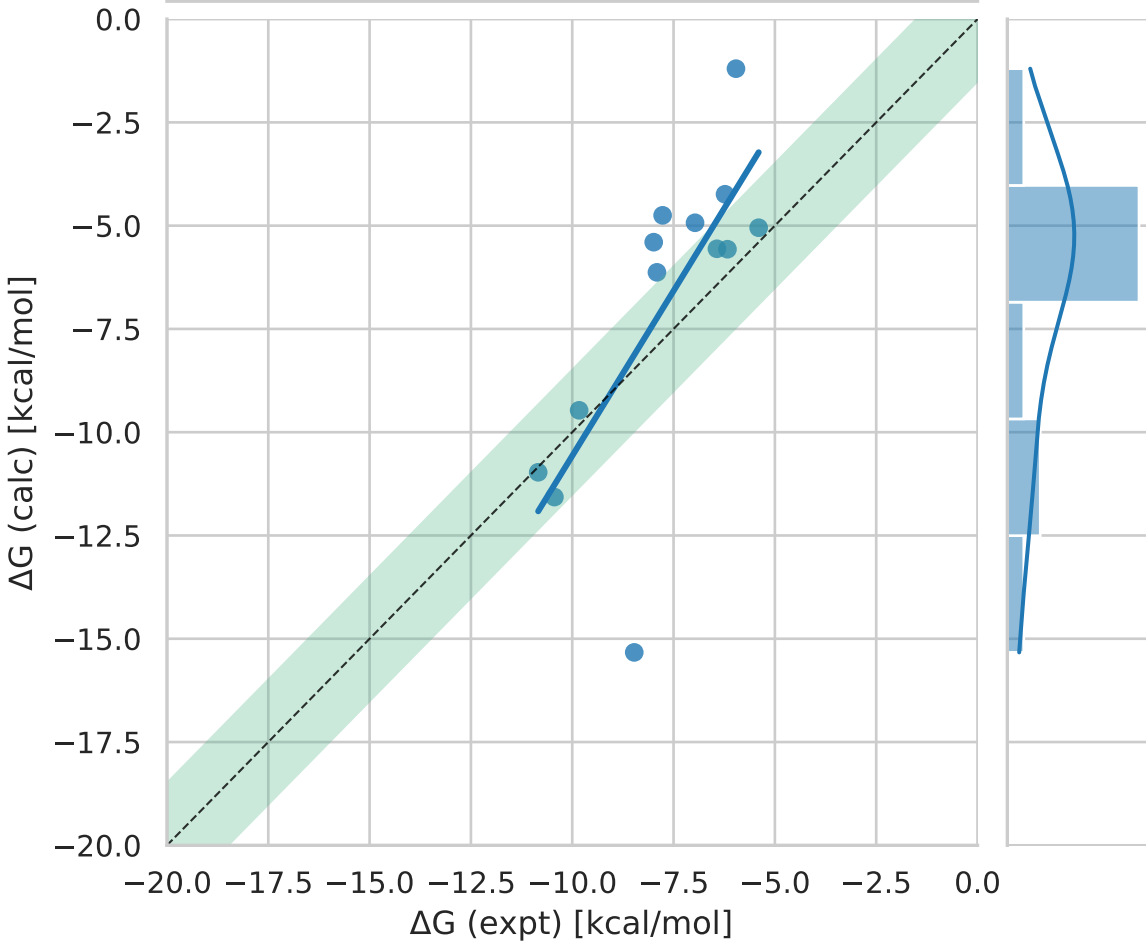

Supplement: CP-026-D3CP05111K-s001 [file CP-026-D3CP05111K-s001.zip › sampl9-supplementary-documents/Users/amezcum1/Desktop/SAMPL9/host_guest/Analysis/Ranked_Accuracy/WP6/FreeEnergyCorrelationPlots/7.pdf]

# ELIE/GAFF2-ABCG2/TIP3P/MD/MMPBSA - WP6 (8)

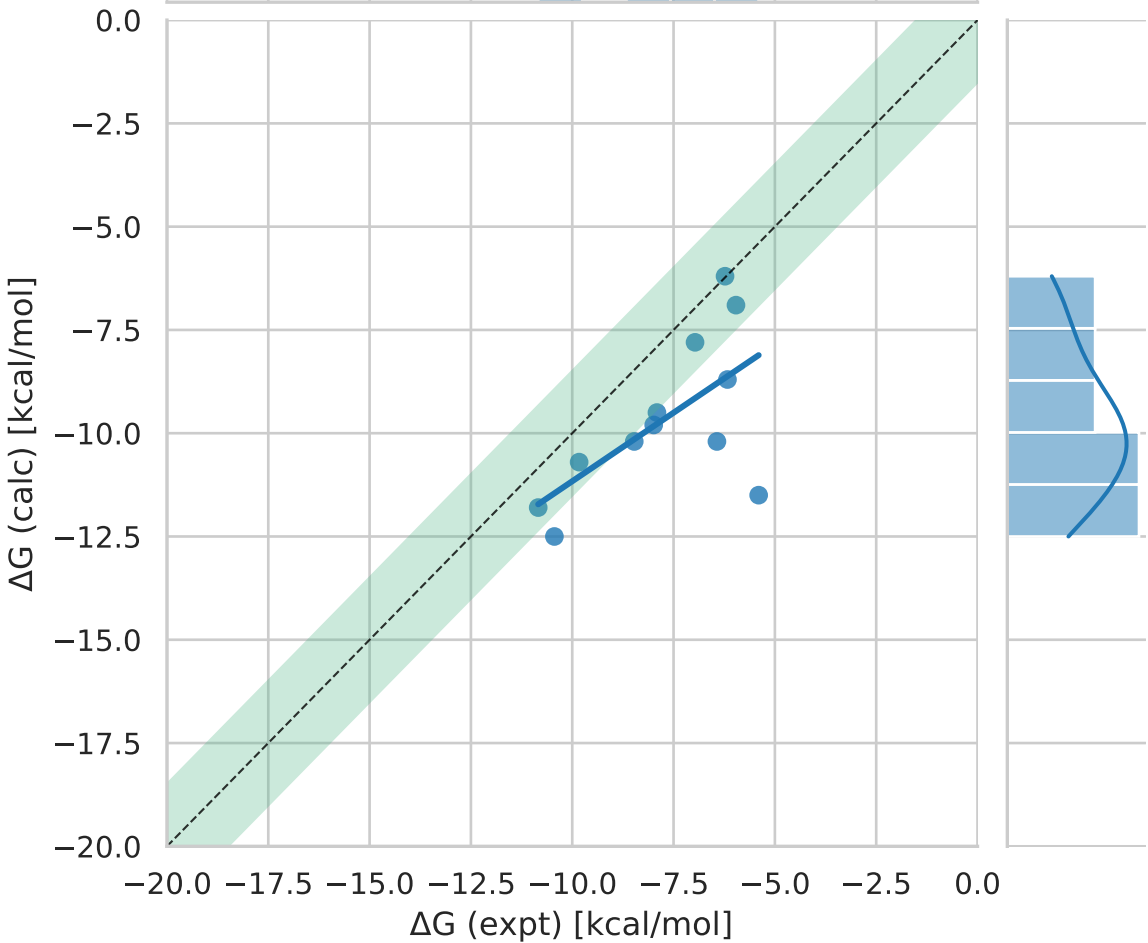

Supplement: CP-026-D3CP05111K-s001 [file CP-026-D3CP05111K-s001.zip › sampl9-supplementary-documents/Users/amezcum1/Desktop/SAMPL9/host_guest/Analysis/Ranked_Accuracy/WP6/FreeEnergyCorrelationPlots/8.pdf]

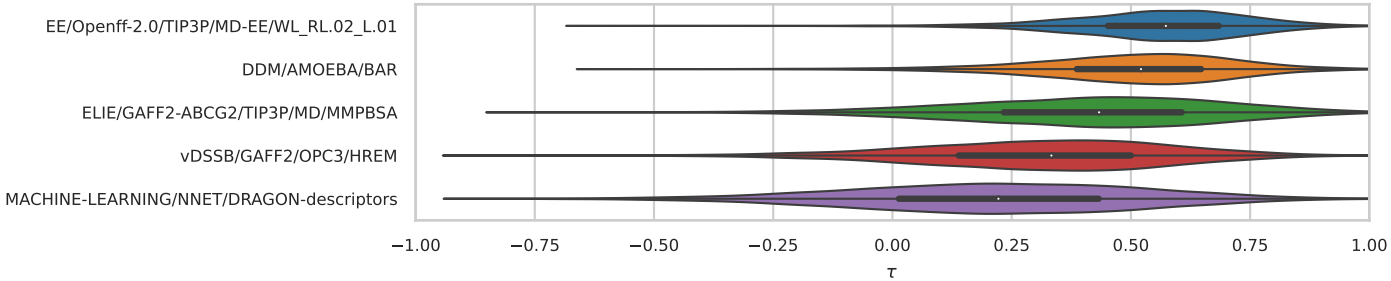

Supplement: CP-026-D3CP05111K-s001 [file CP-026-D3CP05111K-s001.zip › sampl9-supplementary-documents/Users/amezcum1/Desktop/SAMPL9/host_guest/Analysis/Ranked_Accuracy/WP6/StatisticsPlots/kendall_tau_bootstrap_distributions.pdf]

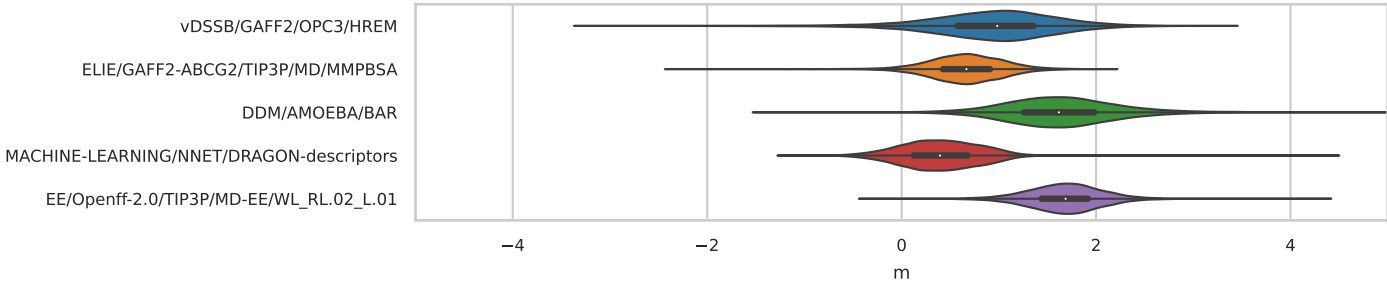

Supplement: CP-026-D3CP05111K-s001 [file CP-026-D3CP05111K-s001.zip › sampl9-supplementary-documents/Users/amezcum1/Desktop/SAMPL9/host_guest/Analysis/Ranked_Accuracy/WP6/StatisticsPlots/m_bootstrap_distributions.pdf]

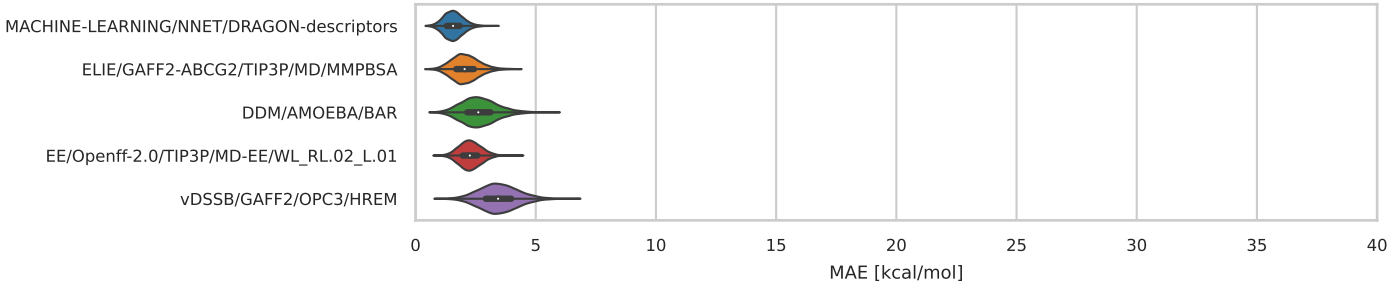

Supplement: CP-026-D3CP05111K-s001 [file CP-026-D3CP05111K-s001.zip › sampl9-supplementary-documents/Users/amezcum1/Desktop/SAMPL9/host_guest/Analysis/Ranked_Accuracy/WP6/StatisticsPlots/MAE_bootstrap_distributions.pdf]

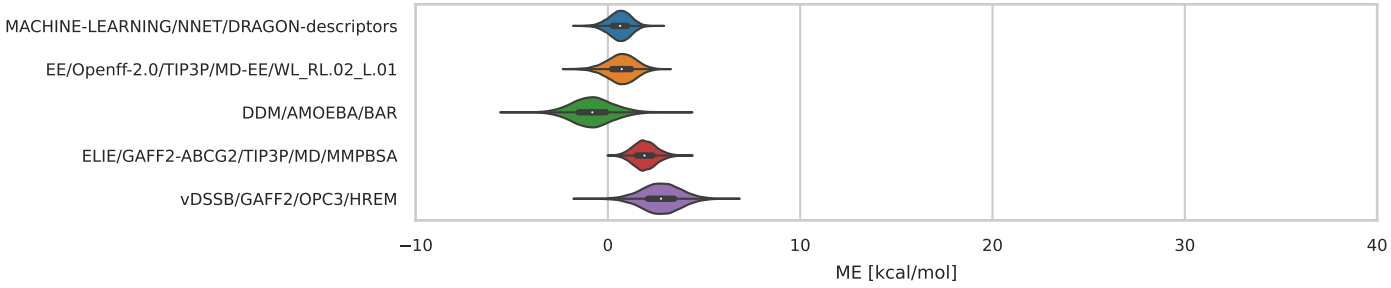

Supplement: CP-026-D3CP05111K-s001 [file CP-026-D3CP05111K-s001.zip › sampl9-supplementary-documents/Users/amezcum1/Desktop/SAMPL9/host_guest/Analysis/Ranked_Accuracy/WP6/StatisticsPlots/ME_bootstrap_distributions.pdf]

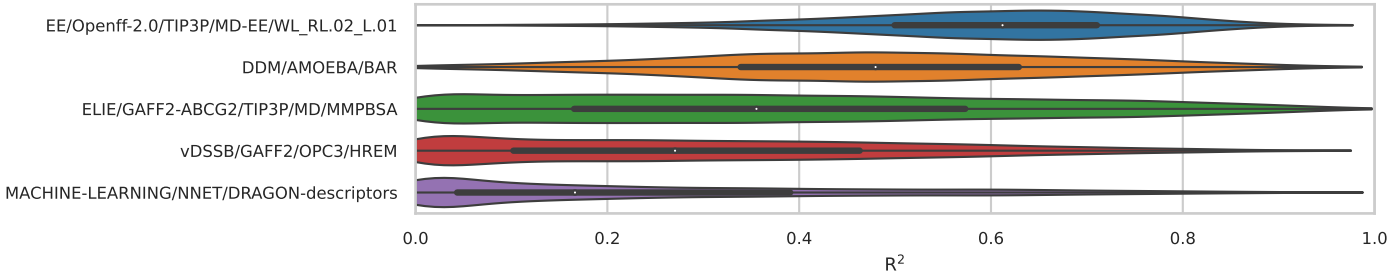

Supplement: CP-026-D3CP05111K-s001 [file CP-026-D3CP05111K-s001.zip › sampl9-supplementary-documents/Users/amezcum1/Desktop/SAMPL9/host_guest/Analysis/Ranked_Accuracy/WP6/StatisticsPlots/R2_bootstrap_distributions.pdf]

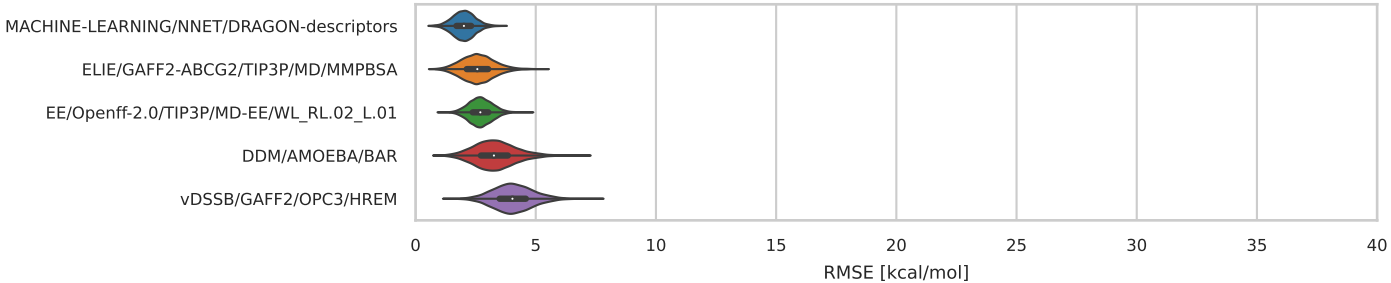

Supplement: CP-026-D3CP05111K-s001 [file CP-026-D3CP05111K-s001.zip › sampl9-supplementary-documents/Users/amezcum1/Desktop/SAMPL9/host_guest/Analysis/Ranked_Accuracy/WP6/StatisticsPlots/RMSE_bootstrap_distributions.pdf]

# MACHINE-LEARNING/NNET/DRAGON-descriptors - WP6 (3)

$\Delta H$  (calc) [kcal/mol]

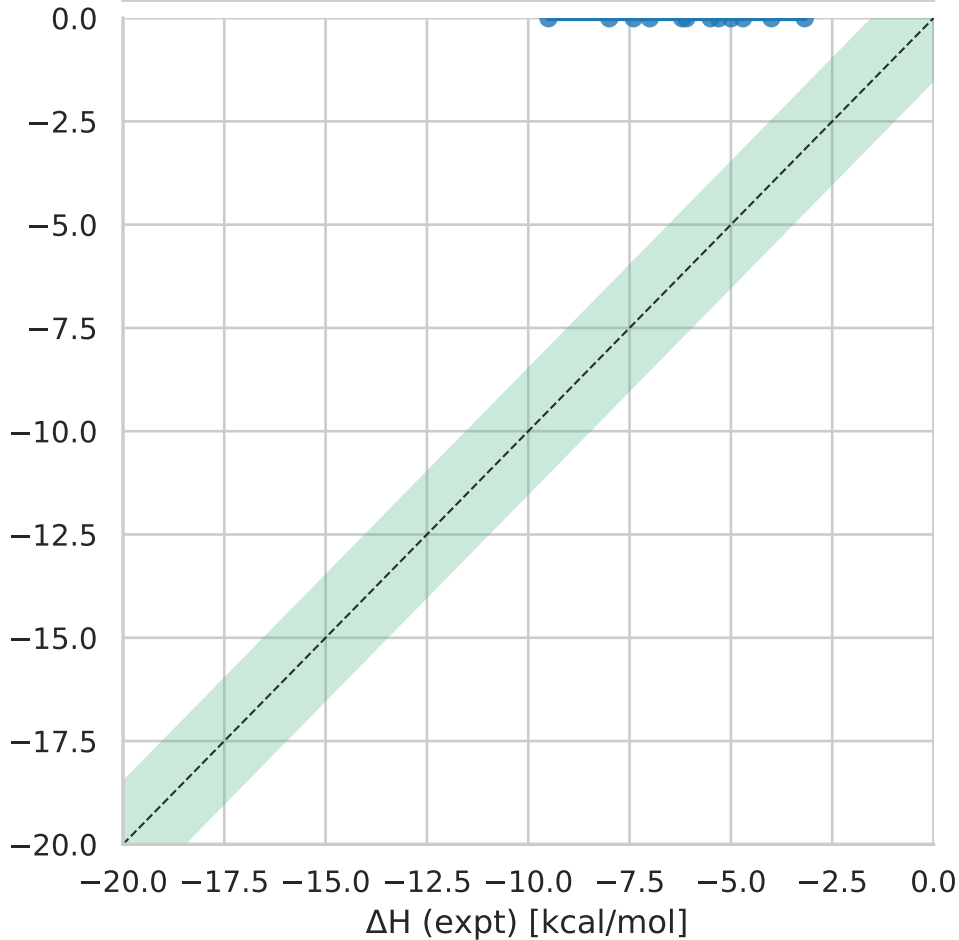

Supplement: CP-026-D3CP05111K-s001 [file CP-026-D3CP05111K-s001.zip › sampl9-supplementary-documents/Users/amezcum1/Desktop/SAMPL9/host_guest/Analysis/Ranked_Accuracy/WP6_no_optional/EnthalpiesCorrelationPlots/3.pdf]

# vDSSB/GAFF2/OPC3/HREM - WP6 (2)

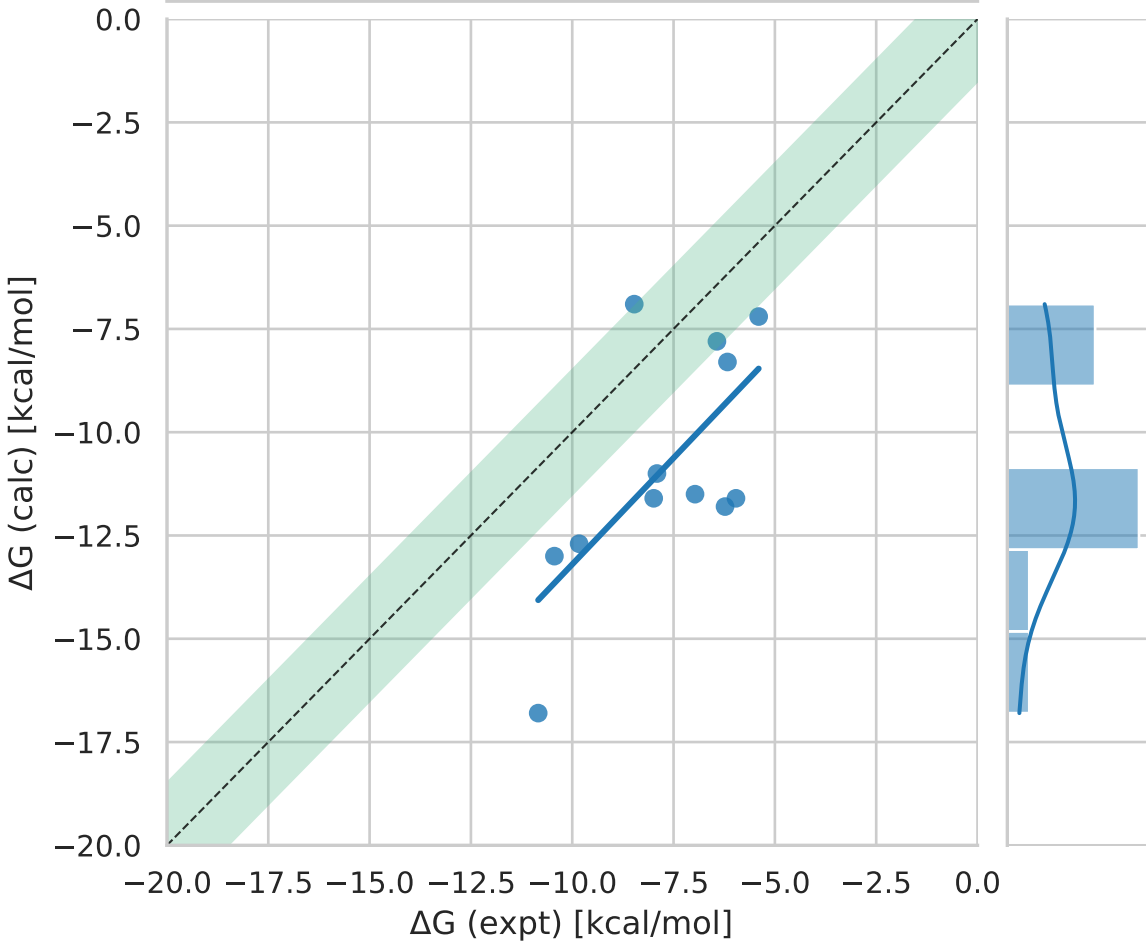

Supplement: CP-026-D3CP05111K-s001 [file CP-026-D3CP05111K-s001.zip › sampl9-supplementary-documents/Users/amezcum1/Desktop/SAMPL9/host_guest/Analysis/Ranked_Accuracy/WP6_no_optional/FreeEnergyCorrelationPlots/2.pdf]

# MACHINE-LEARNING/NNET/DRAGON-descriptors - WP6 (3)

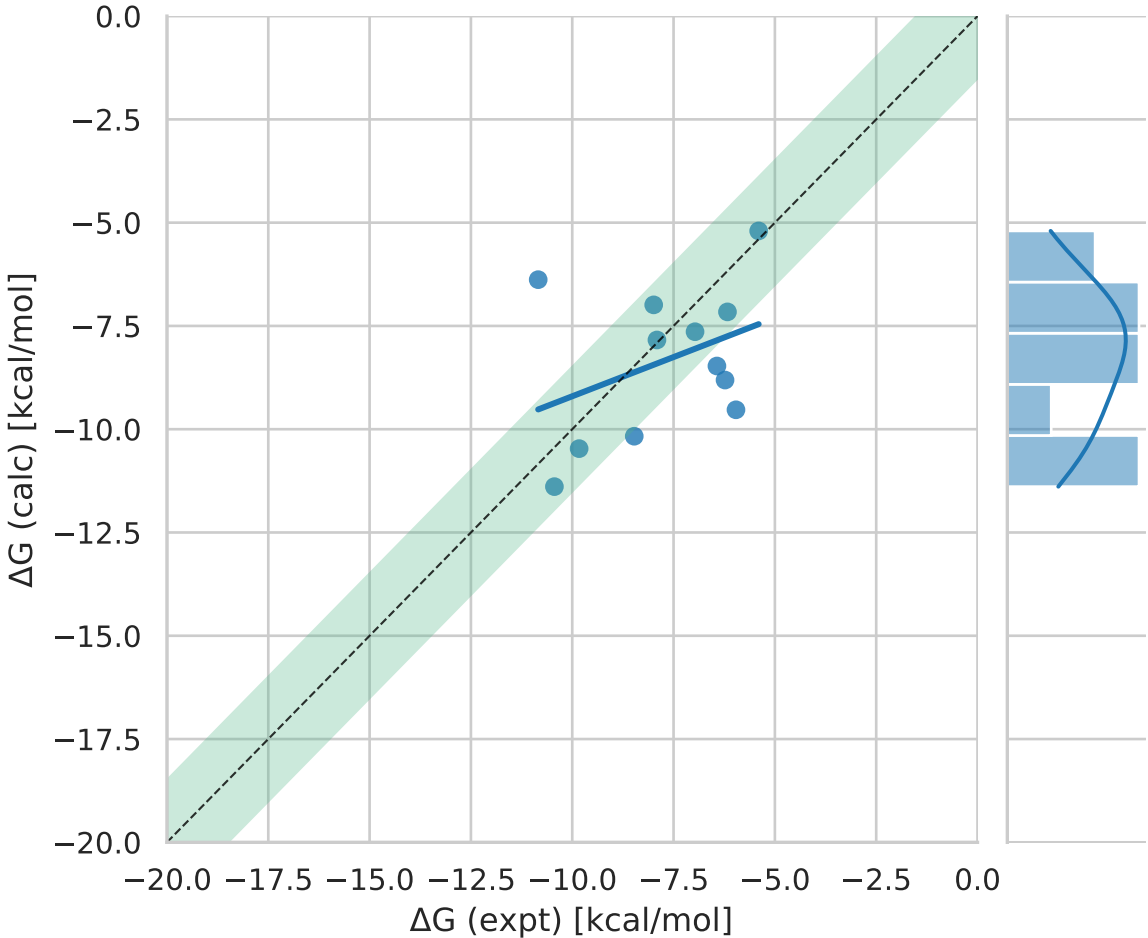

Supplement: CP-026-D3CP05111K-s001 [file CP-026-D3CP05111K-s001.zip › sampl9-supplementary-documents/Users/amezcum1/Desktop/SAMPL9/host_guest/Analysis/Ranked_Accuracy/WP6_no_optional/FreeEnergyCorrelationPlots/3.pdf]

EE/Openff-2.0/TIP3P/MD-EE/WL\_RL.02\_L.01 - WP6 (4)

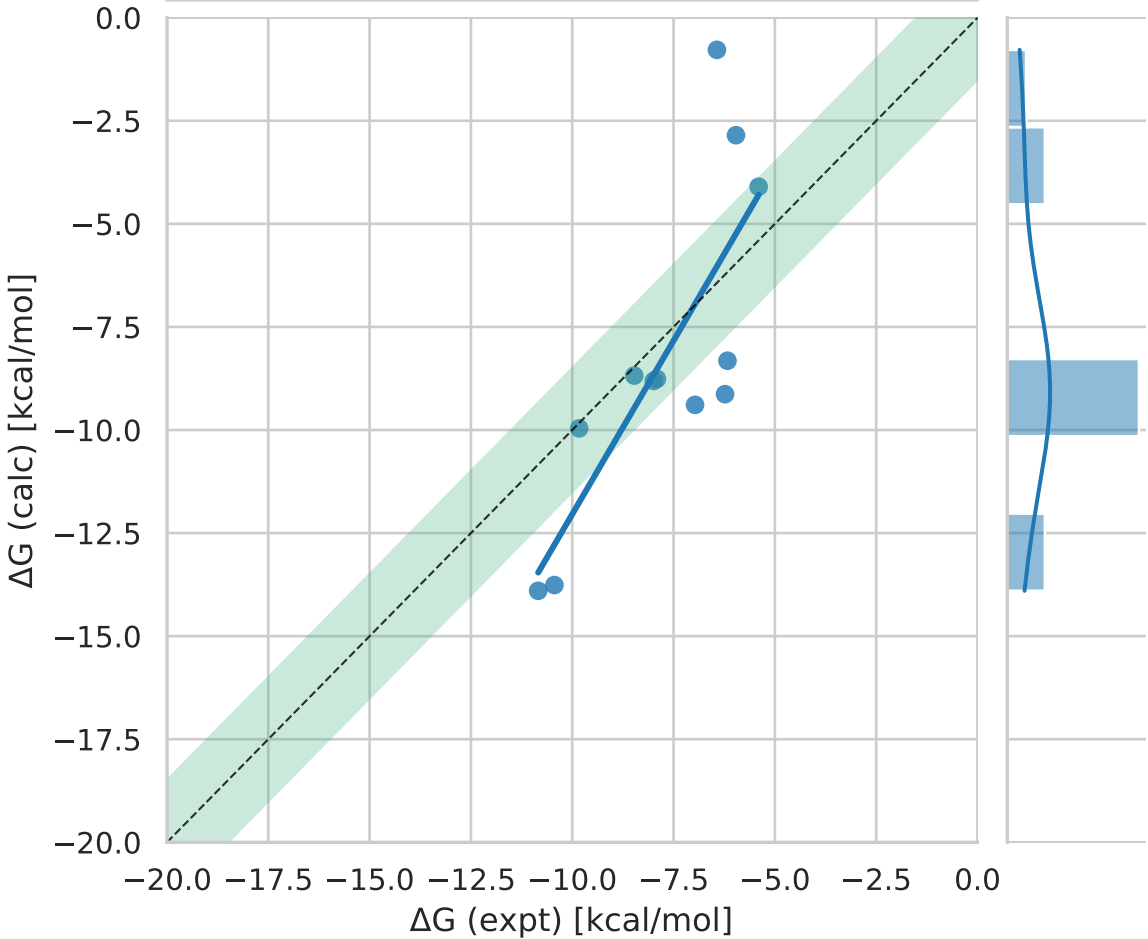

Supplement: CP-026-D3CP05111K-s001 [file CP-026-D3CP05111K-s001.zip › sampl9-supplementary-documents/Users/amezcum1/Desktop/SAMPL9/host_guest/Analysis/Ranked_Accuracy/WP6_no_optional/FreeEnergyCorrelationPlots/4.pdf]

DDM/AMOEBA/BAR - WP6 (7)

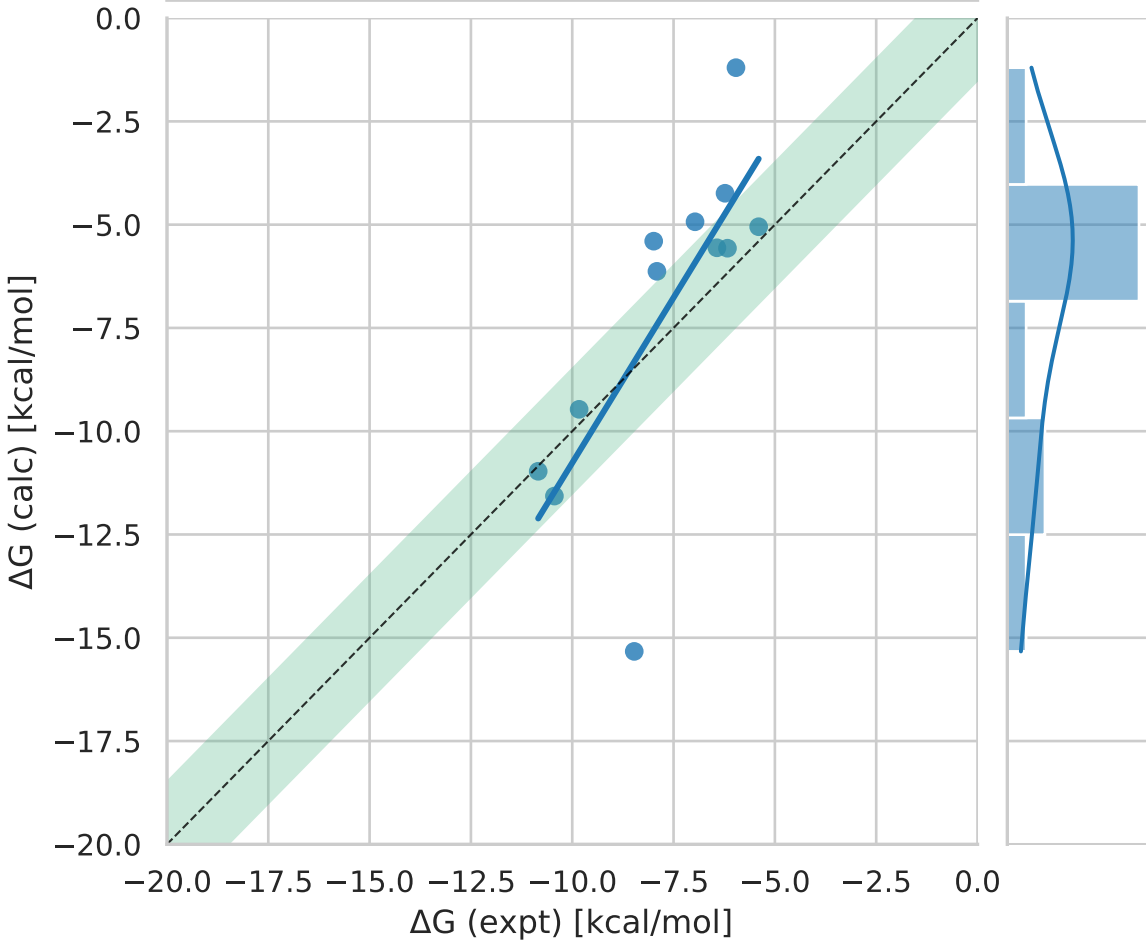

Supplement: CP-026-D3CP05111K-s001 [file CP-026-D3CP05111K-s001.zip › sampl9-supplementary-documents/Users/amezcum1/Desktop/SAMPL9/host_guest/Analysis/Ranked_Accuracy/WP6_no_optional/FreeEnergyCorrelationPlots/7.pdf]

# ELIE/GAFF2-ABCG2/TIP3P/MD/MMPBSA - WP6 (8)

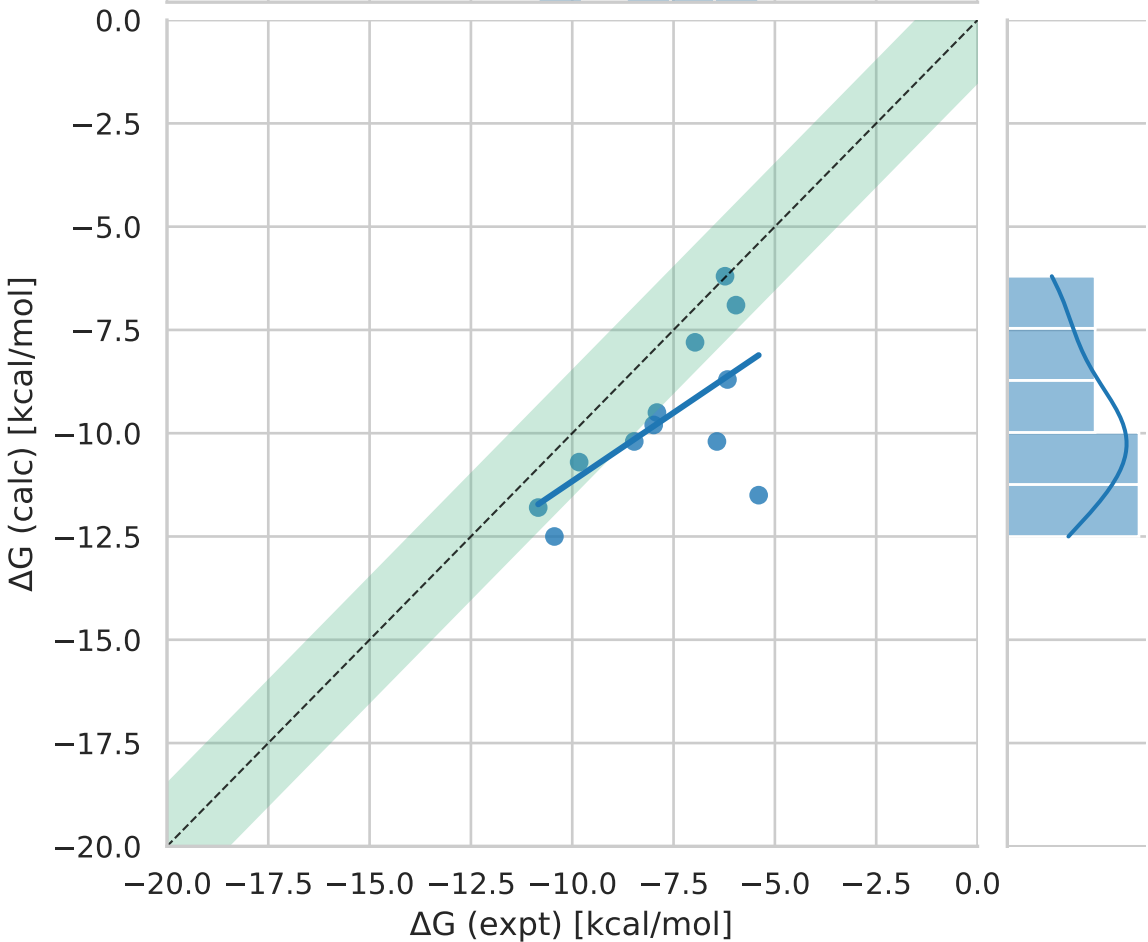

Supplement: CP-026-D3CP05111K-s001 [file CP-026-D3CP05111K-s001.zip › sampl9-supplementary-documents/Users/amezcum1/Desktop/SAMPL9/host_guest/Analysis/Ranked_Accuracy/WP6_no_optional/FreeEnergyCorrelationPlots/8.pdf]

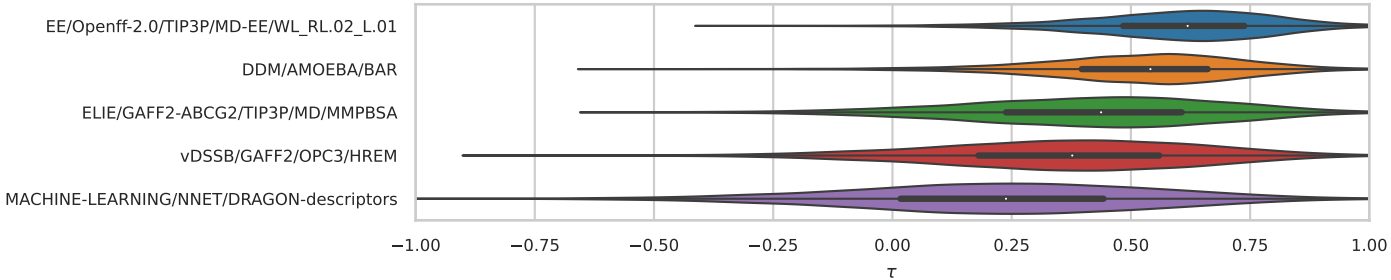

Supplement: CP-026-D3CP05111K-s001 [file CP-026-D3CP05111K-s001.zip › sampl9-supplementary-documents/Users/amezcum1/Desktop/SAMPL9/host_guest/Analysis/Ranked_Accuracy/WP6_no_optional/StatisticsPlots/kendall_tau_bootstrap_distributions.pdf]

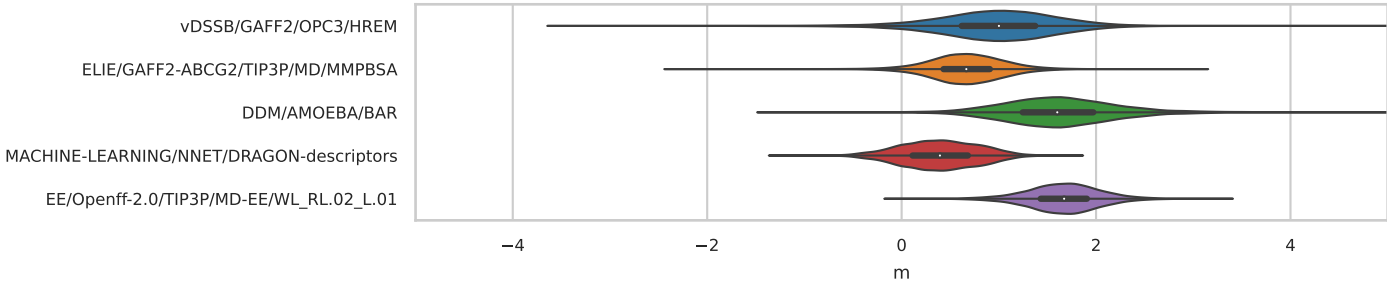

Supplement: CP-026-D3CP05111K-s001 [file CP-026-D3CP05111K-s001.zip › sampl9-supplementary-documents/Users/amezcum1/Desktop/SAMPL9/host_guest/Analysis/Ranked_Accuracy/WP6_no_optional/StatisticsPlots/m_bootstrap_distributions.pdf]

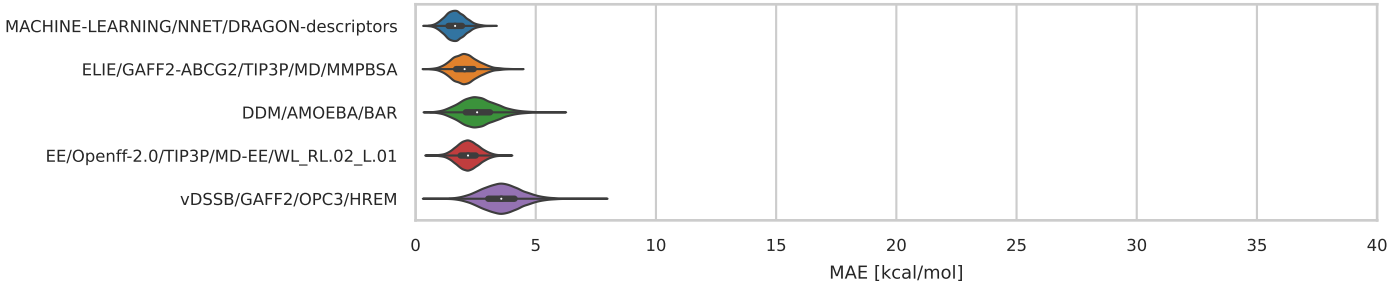

Supplement: CP-026-D3CP05111K-s001 [file CP-026-D3CP05111K-s001.zip › sampl9-supplementary-documents/Users/amezcum1/Desktop/SAMPL9/host_guest/Analysis/Ranked_Accuracy/WP6_no_optional/StatisticsPlots/MAE_bootstrap_distributions.pdf]

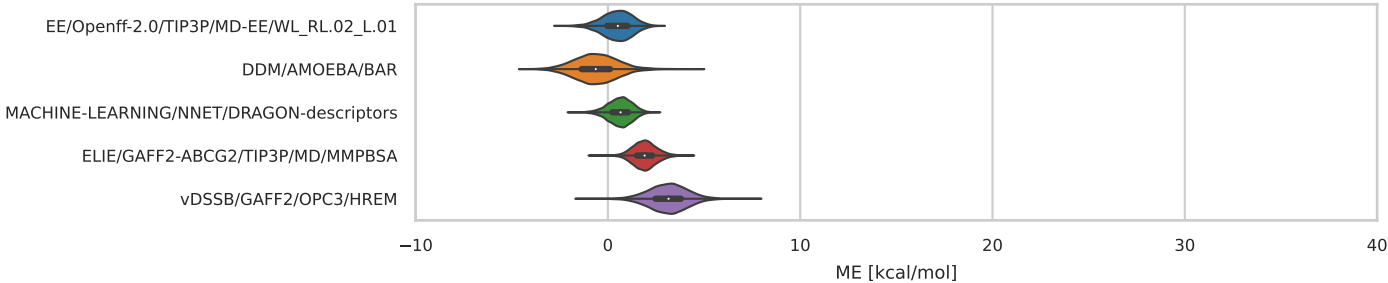

Supplement: CP-026-D3CP05111K-s001 [file CP-026-D3CP05111K-s001.zip › sampl9-supplementary-documents/Users/amezcum1/Desktop/SAMPL9/host_guest/Analysis/Ranked_Accuracy/WP6_no_optional/StatisticsPlots/ME_bootstrap_distributions.pdf]

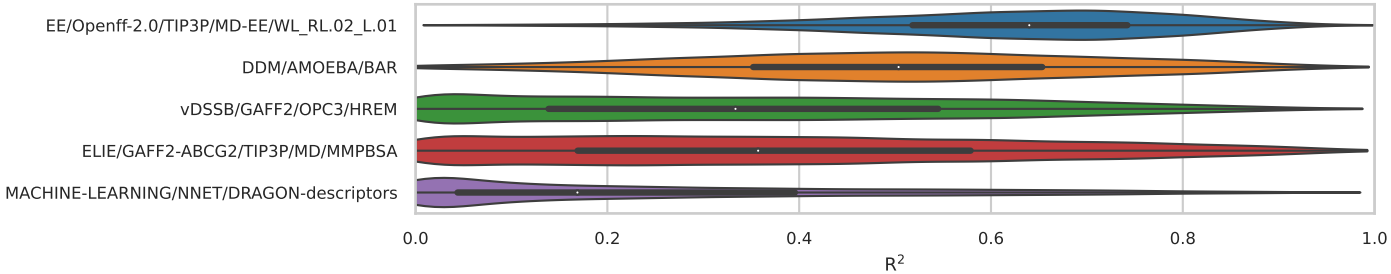

Supplement: CP-026-D3CP05111K-s001 [file CP-026-D3CP05111K-s001.zip › sampl9-supplementary-documents/Users/amezcum1/Desktop/SAMPL9/host_guest/Analysis/Ranked_Accuracy/WP6_no_optional/StatisticsPlots/R2_bootstrap_distributions.pdf]

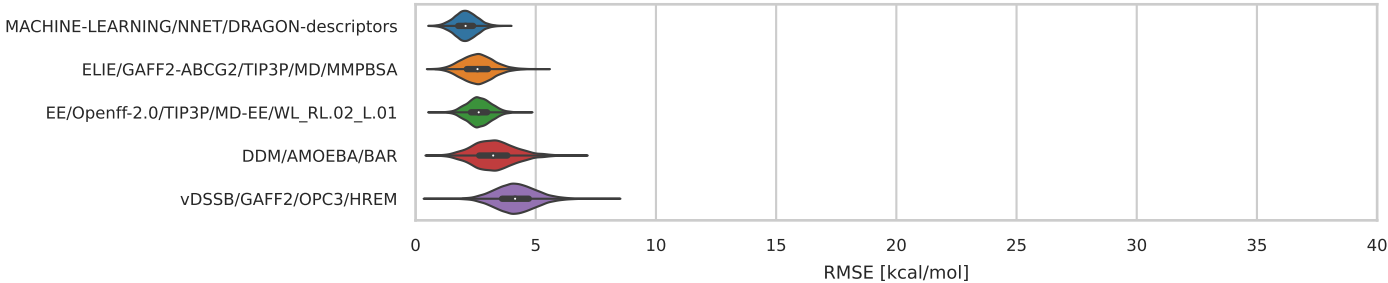

Supplement: CP-026-D3CP05111K-s001 [file CP-026-D3CP05111K-s001.zip › sampl9-supplementary-documents/Users/amezcum1/Desktop/SAMPL9/host_guest/Analysis/Ranked_Accuracy/WP6_no_optional/StatisticsPlots/RMSE_bootstrap_distributions.pdf]
