# Supplementary material for: The SAMPL9 host–guest blind challenge: an overview of binding free energy predictive accuracy
Source: Phys Chem Chem Phys. 2024 Mar 4;26(12):9207–25. doi: 10.1039/d3cp05111k (PMC10954238; doi:10.1039/d3cp05111k)
Supplement: CP-026-D3CP05111K-s002 [file CP-026-D3CP05111K-s002.pdf]

Electronic Supplementary Material (ESI) for Physical Chemistry Chemical Physics.
This journal is © the Owner Societies 2024

Supplemental Information

The SAMPL9 Host-Guest Blind Challenge: An Overview of Binding Free Energy Predictive Accuracy

Martin Amezcua, Jeffry Setiadi, and David L. Mobley

Table S1 Summary of host and guest anchor atoms chosen for reference calculations with APR method. Shown here are the atom names (based on the PDB / MOL2 / SDF files provided in the SAMPL9 GitHub repository) host and guest anchor atoms chosen for reference calculations with the APR method. Three host anchor atoms were chosen (H1, H2, H3) manually. Two guest anchor atoms are chosen (L1 and L2) manually. Reference calculations did not include the optional system WP6-G4.

**HOST ANCHOR ATOMS SYSTEM ID**	**H1**	**H2**	**H3**
WP6	C14	C58	C36
bCD	C4	C16	C25
HbCD	C4	C16	C25
**GUEST ANCHOR ATOMS SYSTEM ID**	**L1**	**L2**	
WP6-G1	C7	C11	
WP6-G2	C3	C1	
WP6-G3	C1	C6	
WP6-G4	-	-	
WP6-G5	C10	C9	
WP6-G6	C8	N1	
WP6-G7	C6	N1	
WP6-G8	C6	C9	
WP6-G9	C7	C2	
WP6-G10	C11	C8	
WP6-G11	C5	N1	
WP6-G12	N1	C5	
WP6-G13	C8	C9	
bCD-PMZ	C1	C9	
bCD-PMT	C2	C10	
bCD-CPZ-pri	C1	C8	
bCD-CPZ-sec	C12	C9	
bCD-TDZ-pri	C1	C8	
bCD-TDZ-sec	C12	C9	
bCD-TFP-pri	C1	C4	
bCD-TFP-sec	C8	C10	
HbCD-PMZ	C1	C9	
HbCD-PMT	C2	C10	
HbCD-CPZ-pri	C1	C8	
HbCD-CPZ-sec	C12	C9	
HbCD-TDZ-pri	C1	C8	
HbCD-TDZ-sec	C12	C9	
HbCD-TFP-pri	C1	C4	
HbCD-TFP-sec	C8	C10	

Table S2 Summary of restraints applied for reference calculations with APR method. The parameters used on the different types of restraints. Within a host class (i.e WP6, bCD, and HbCD), the force constants (k), angle, or distance of each guest restraint were the same. If optional restraints were not applied (i.e Conformation or Wall restraints), the column is left empty.

**Host Static Restraints**		
**SYSTEM ID**	**Distance (kcal/mol/Å^2)**	**Angle (kcal/mol/rad^2)**
WP6	10.0	100.0
bCD	10.0	100.0
HbCD	10.0	100.0
**Guest Translational and Rotational Restraints**		
**SYSTEM ID**	**Distance (kcal/mol/Å^2)**	**Angle (kcal/mol/rad^2)**
WP6	10.0	100.0
bCD	10.0	100.0
HbCD	10.0	100.0
**Host Conformational (Dihedral) Restraints**		
**SYSTEM ID**	**k jack (kcal/mol/rad^2)**	**Dihedral angle jack (°)**
WP6	-	-
bCD-dih1	6.0	-112.5
bCD-dih2	6.0	108.7
HbCD-dih1	6.0	-112.5
HbCD-dih2	6.0	108.7
**Wall Restraints**		
**SYSTEM ID**	**k wall (kcal/mol/Å^2)**	**r wall (Å)**
WP6	50.0	14.0
bCD	50.0	14.0
HbCD	50.0	14.0

Table S3 ΔΔG error of optional WP6-G4 system for all SAMPL9 submissions The predicted binding free energy (calculated) of all methods for optional WP6-G4 system, and ΔΔG error (kcal/mol) from ΔG calculated minus ΔG experimental (-7.77 kcal/mol). Submissions that did not include a WP6-G4 prediction are filled with DNS (did not submit). Each unique method has an assigned submission ID (sid), a ranked submission is denoted with an asterisk next to the method name, and a reference submission is flagged with a double asterisk. Expanded ensemble methods (sids 4, 5, 6, and 17) modeled G4 using GAFF2.11, since OpenFF2.0.0 parameters were not available.

**ID**	**sid**	**ΔG (calc) [kcal/mol]**	**ΔΔG error (calc-expt) [kcal/mol]**
**WP6**			
DOCKING/SMINA/VINARDO	1	-5.11 ± 0.10	2.66
*MACHINE-LEARNING/NNET/DRAGON-descriptors**	3	-8.35 ± 0.57	-0.58
*ELIE/GAFF2-ABCG2/TIP3P/MD/MMPBSA**	8	**DNS**	**DNS**
EE/Openff-2.0/TIP3P/MD-EE/All_data	5	-10.78 ± 1.10	-3.01
*EE/Openff-2.0/TIP3P/MD-EE/WL_RL.02_L.01**	4	-10.87 ± 1.11	-3.10
*DDM/AMOEBA/BAR**	7	-4.75 ± 0.05	3.02
EE/Openff-2.0/TIP3P/MD-EE/RL_8_only	6	-11.14 ± 0.26	-3.37
EE/Openff-2.0/TIP3P/MD-EE/WL_RL.02_L.01/corrected	17	-11.35 ± 1.11	-3.58
*APR/OFF1.2.0/TIP3P/MD-US/MBAR***	19	**DNS**	**DNS**
*vDSSB/GAFF2/OPC3/HREM**	2	-6.20 ± 0.20	1.57
*APR/OFF2.0.0/TIP3P/MD-US/MBAR***	20	**DNS**	**DNS**
*APR/GAFF2/TIP3P/MD-US/MBAR***	18	**DNS**	**DNS**

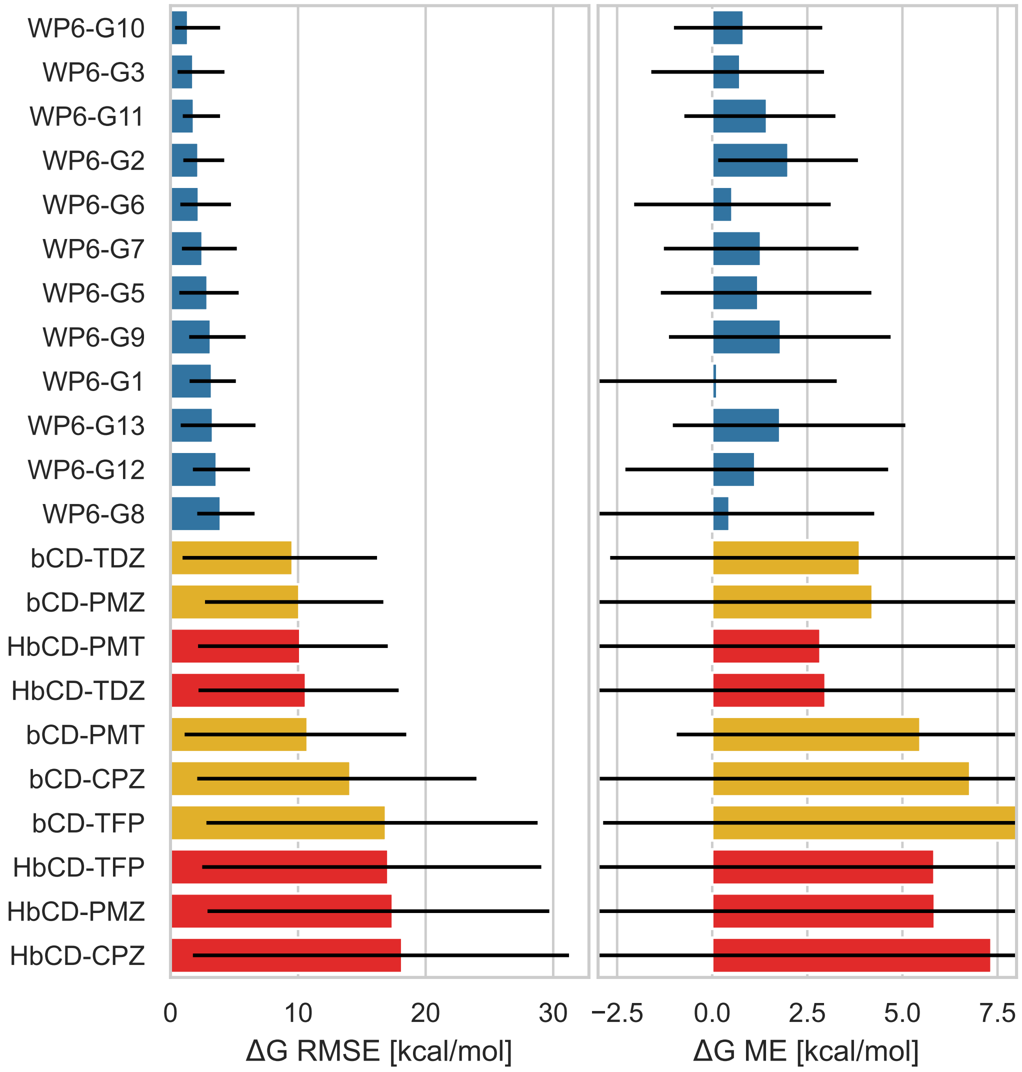

Figure S1 Error statistics (RMSE and ME) of ranked predictions for each host-guest system Binding free energy RMSE and ME by host-guest system, where each host-guest system category is color coded by host (WP6 in blue, bCD in yellow, and HbCD in red). The RMSE and ME for absolute binding ΔG were calculated via bootstrapping with 100,000 replacement samples, including experimental uncertainties, of all host-guest system predictions. The black error bars represent 95-percentile bootstrap confidence intervals. Optional system WP6-G4 was not included.

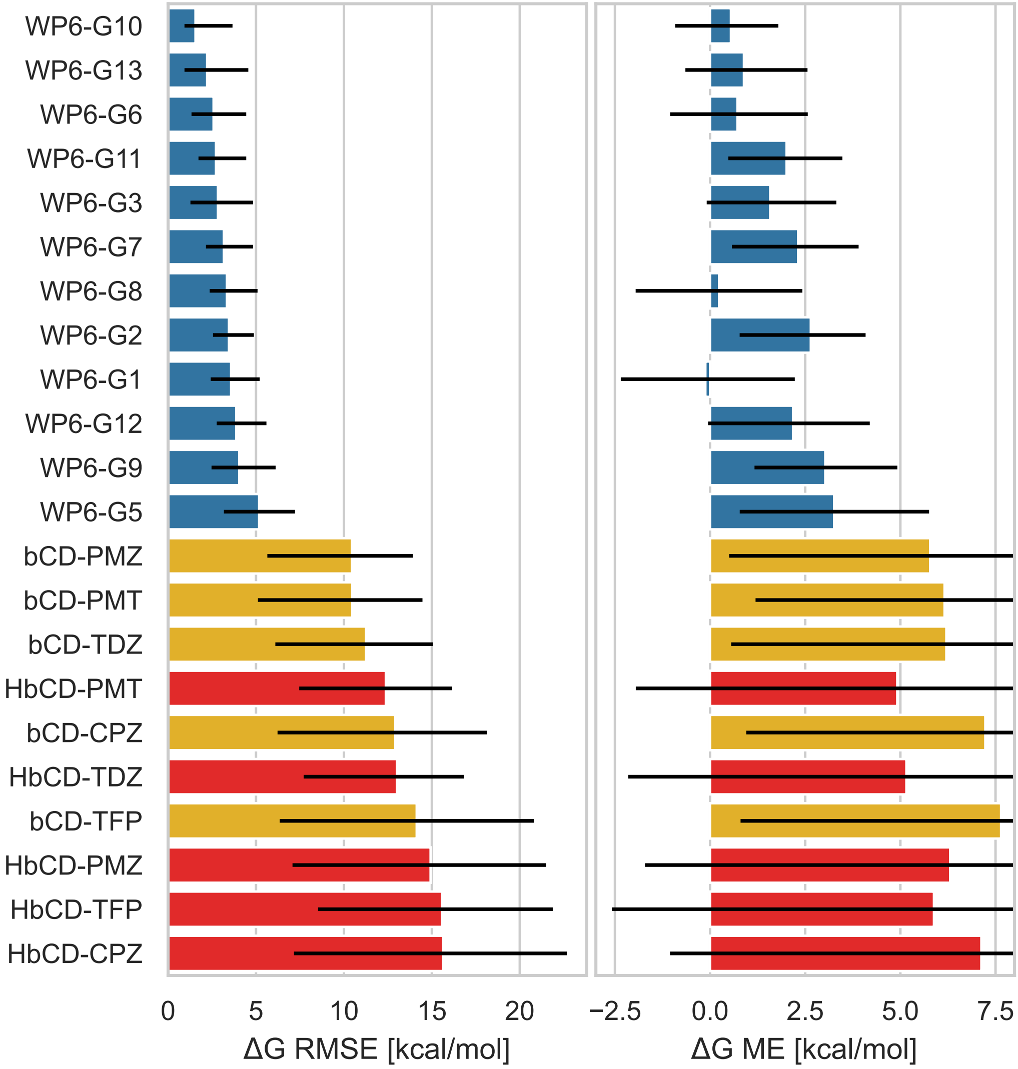

Figure S2 Error statistics (RMSE and ME) of all methods predictions for each host-guest system Binding free energy RMSE and ME by host-guest system for all methods (ranked and non-ranked combined). Each host-guest system category is color coded by host (WP6 in blue, bCD in yellow, and HbCD in red). The RMSE and ME for absolute binding ΔG were calculated via bootstrapping with 100,000 replacement samples, including experimental uncertainties, of all host-guest system predictions. The black error bars represent 95-percentile bootstrap confidence intervals. Optional system WP6-G4 was not included.

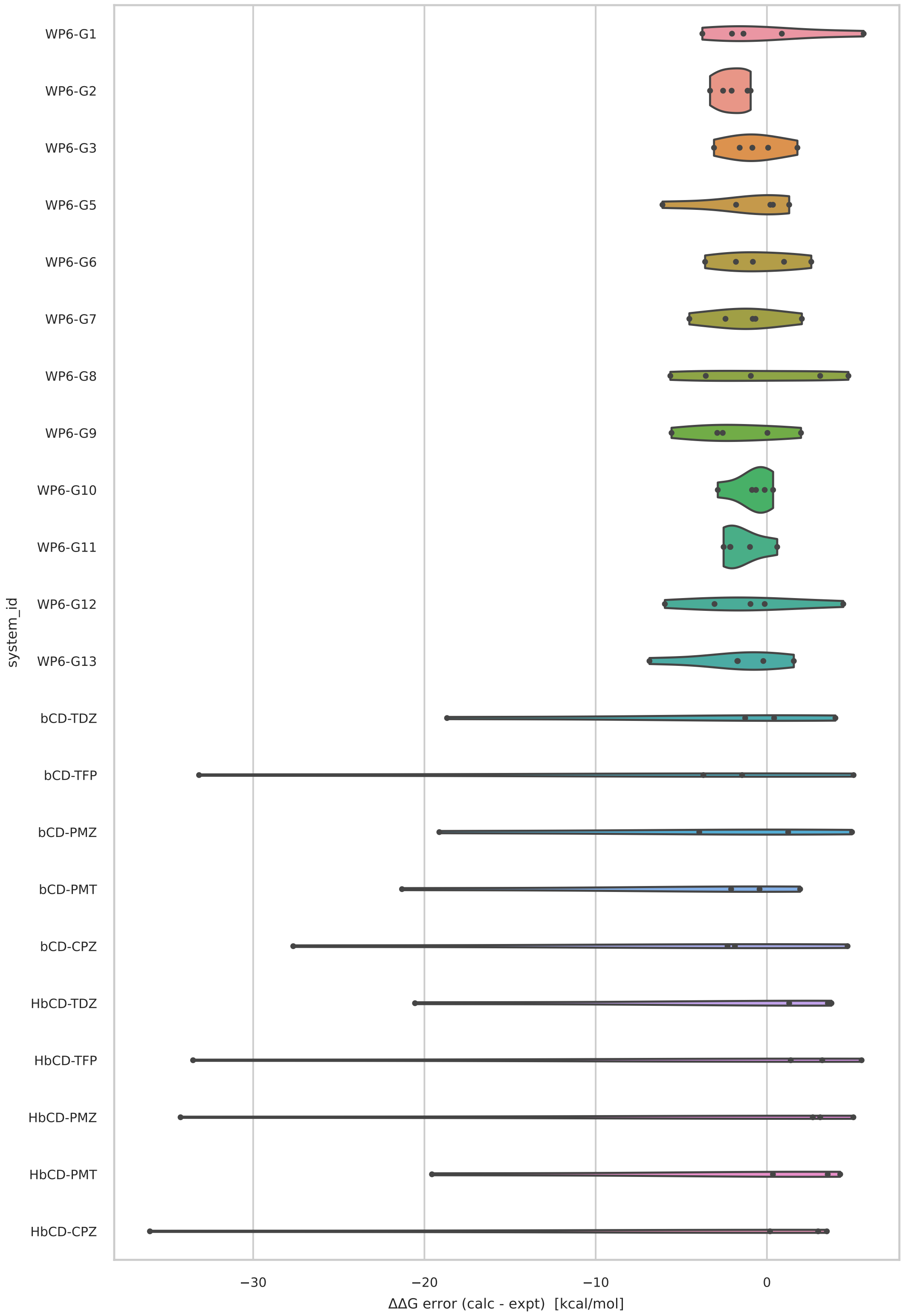

Figure S3 ΔΔG error of ranked predictions for each host-guest system (calculated versus experiment) The ΔΔG error of calculated versus experiment (in kcal/mol) is represented by black dots. The distribution of the ΔΔG is highlighted for each host-guest system

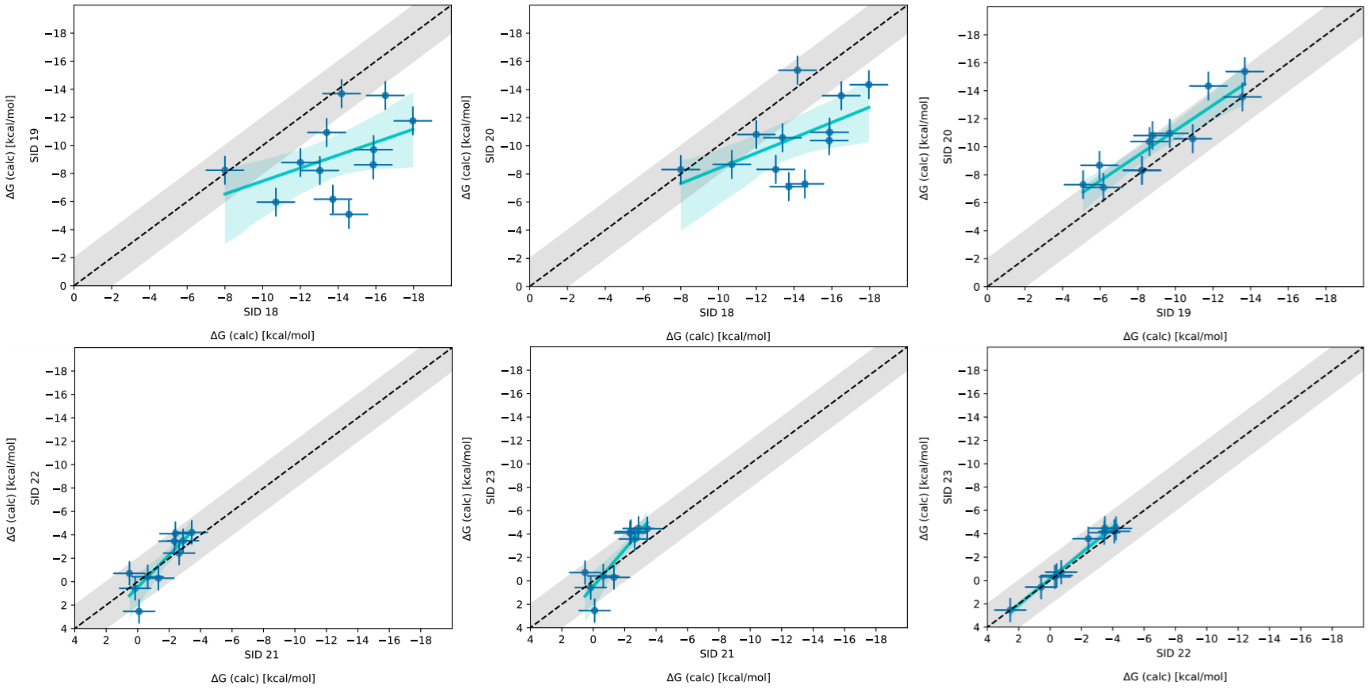

Figure S4 Correlation plots comparing force fields used in reference methods on the WP6 and CD datasets. Plots comparing the force fields used in reference methods for predicting BFEs (kcal/mol) in the SAMPL9 host-guest challenge. The gray shaded regions represents 2 kcal/mol error, the cyan solid line is linear regression, and the cyan shaded region is the confidence interval. The submission IDs (SIDs) are shown in the respective x and y axis. Top row compares force fields used for the WP6 dataset, and the bottom row for the bCD/HbCD dataset. The force fields compared in each plot are: (*left panels*) GAFF2 and OpenFF1.2.0, (*middle panels*) GAFF2 and OpenFF2.0.0, (*right panels*) OpenFF1.2.0 and OpenFF2.0.0.

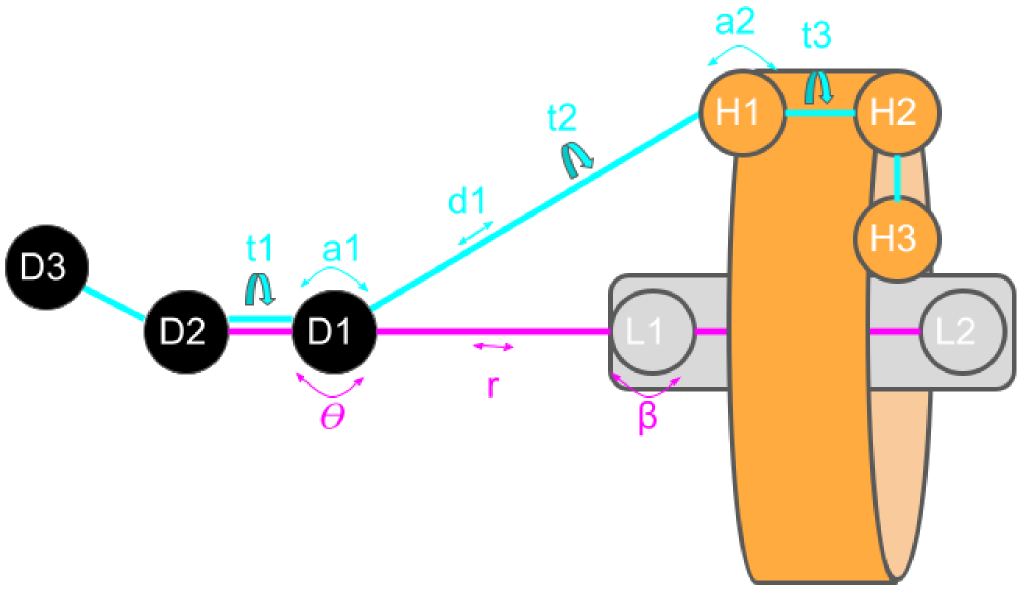

Figure S5 Schematic representation of APR restraints setup of SAMPL9 host-guest datasets. A general schematic of the restraints used for attach-pull-release (APR) binding free energy (BFE) calculations. The host is represented by the orange cylinder shape with anchor atoms (H1, H2, and H3), the guest by the gray rectangle with guest anchor atoms (L1 and L2), and dummy atoms (D1, D2, and D3) in black. The host and guest restraints required for APR simulations (shown in this schematic) are defined and setup with the help of the dummy atoms relative to the host and guest anchor atoms. Shown in cyan are Boresch-style restraints composed of three torsions (**t1**, **t2**, and **t3**), two angles (**a1** and **a2**), and one distance (**d1**) restraints, imposed throughout the entire simulation on the host to control its translational and orientational (rotational) degrees of freedom. We note that these restraints do not perturb the host's conformational degrees of freedom. **t1** is defined by D3, D2, D1, and H1. **t2** is defined by D2, D1, H1, and H2. **t3** is defined by D1, H1, H2, and H3. **a1** is defined by D2, D1, and H1. **a2** is defined by D1, H1, and H2. **d1** is defined by D1 and H1. In addition, shown in magenta are three restraints (two angles (θ and β) and one distance (**r**)) imposed and attached on the guest over a series of simulation windows in the attach phase, and used to translate the guest out of the host cavity by increasing **r** in intervals of 0.4 Å creating the pull phase simulation windows.

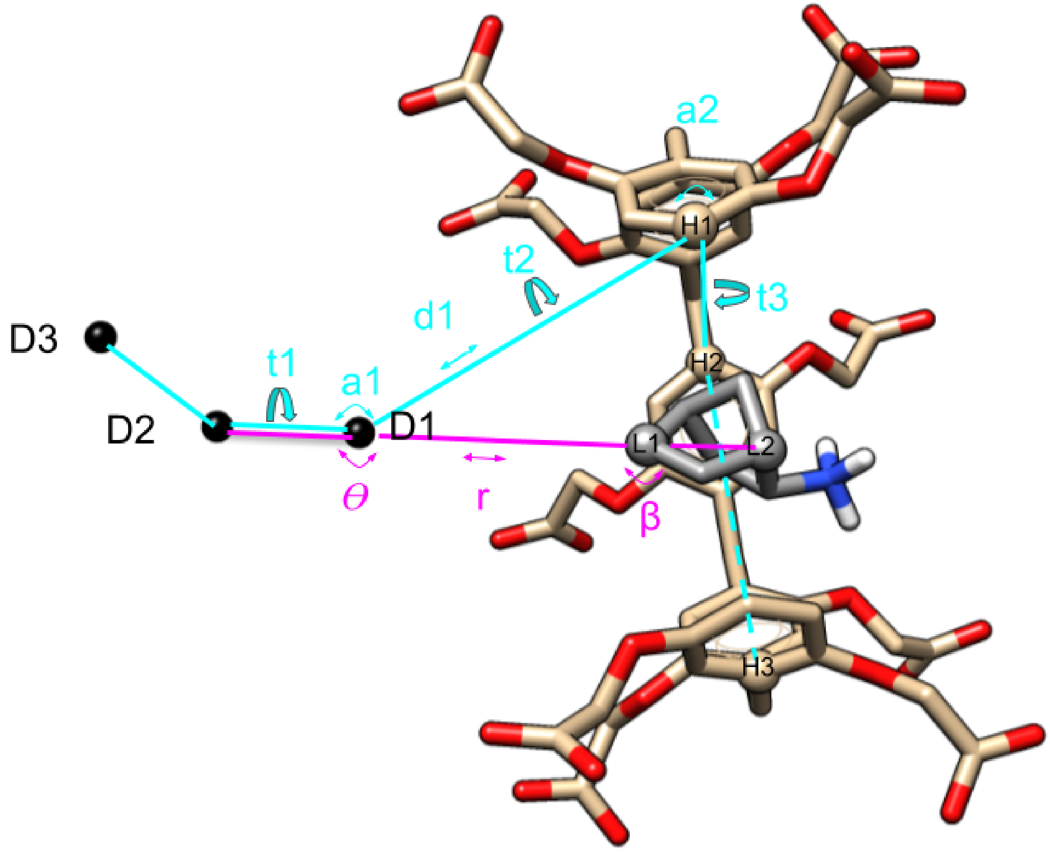

Figure S6 APR host-guest restraint setup for SAMPL9 WP6 dataset. Following the schematic in figure S5, shown is the WP6-G3 complex with the host-guest restraint setup for APR binding free energy calculations for this dataset, and to give the spatial relationship between dummy atoms, and guest and host anchor atoms. The WP6 host (in beige) and guest (in gray) anchor atoms selected are shown by spheres and labeled with H1, H2, H3, L1, or L2. The boresch-style restraints imposed on the host are shown in cyan, and three restraints imposed on the guest are shown in magenta. The cyan dashed line connecting H2 and H3 is for clarity, to prevent the guest from being blocked. A phenylene group and its two carboxylate arms were removed for visualization purposes. The specific WP6 and guest anchor atoms selected are in Table S1 and the force constants in Table S2.

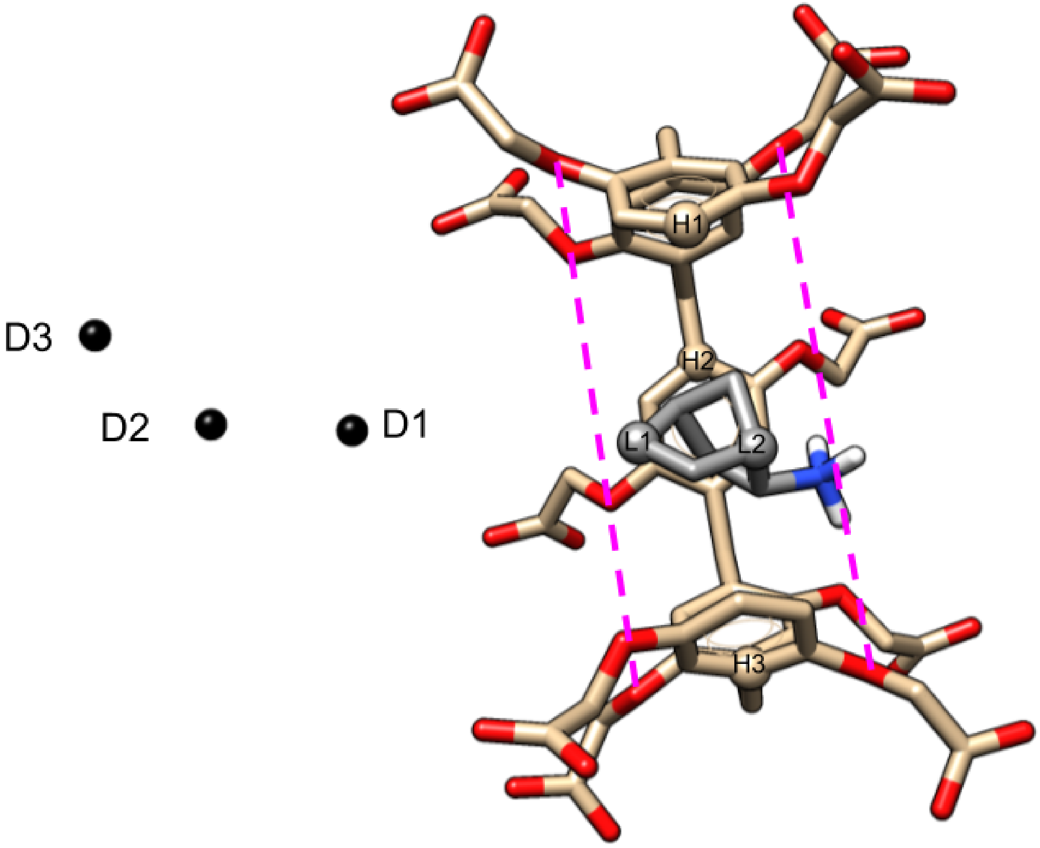

Figure S7 APR wall restraints setup for SAMPL9 WP6 dataset. Following the schematic in figure S5, shown is the WP6-G3 complex with the wall restraints used for APR binding free energy calculations for this dataset. A phenylene group and its two carboxylate arms were removed for visualization purposes. The magenta dashed lines represent the wall created via harmonic flat-bottom restraints imposed to keep the guest (via the L1 guest anchor atom) in the WP6 host cavity. The two ether oxygen atoms (O1, O31, O25, O19, 013, O7, O2, O32, O26, O20, O14, and O8) of each phenylene subunit were used to define the wall restraints. The force constant and distance criteria used for wall restraints are in Table S2.

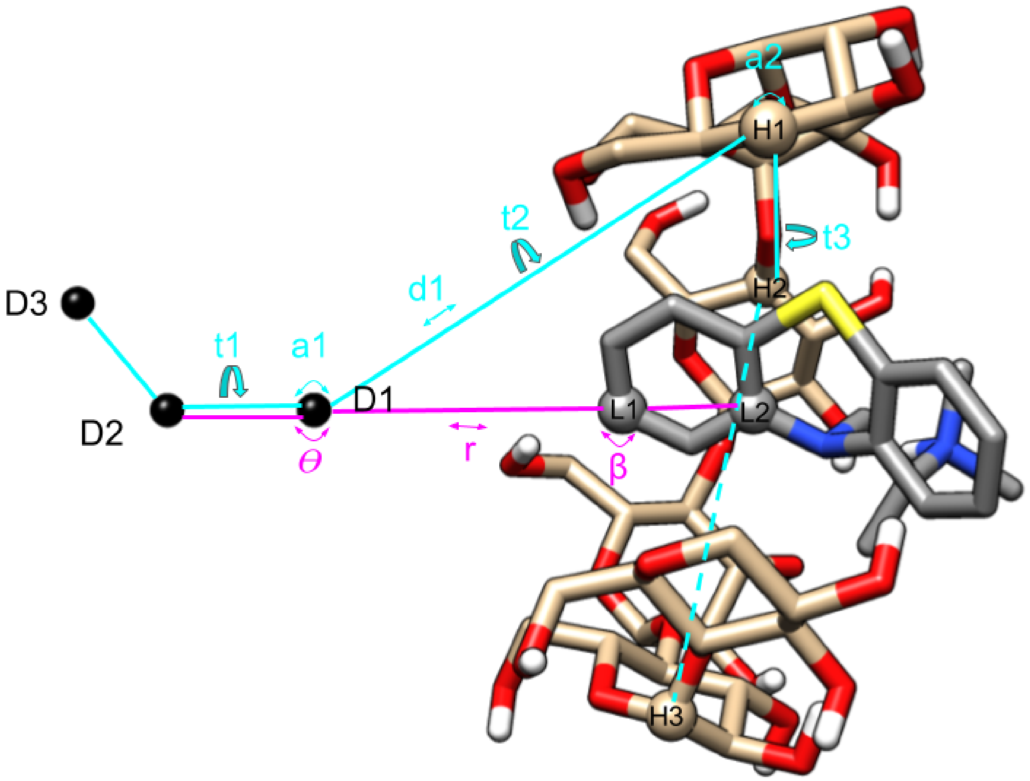

Figure S8 APR host-guest restraint setup for SAMPL9 cyclodextrin (bCD and HbCD) dataset. Following the schematic in figure S5, shown is the bCD-PMT complex with the host-guest restraint setup for APR binding free energy calculations for this dataset, depicting the spatial relationship between dummy atoms, and guest and host anchor atoms. The bCD host (in beige) and guest (in gray) anchor atoms selected are shown by spheres and labeled with H1, H2, H3, L1, or L2. The boresch-style restraints imposed on the bCD host are shown in cyan, and three restraints imposed on the PMT guest are shown in magenta. The cyan dashed line connecting H2 and H3 is for clarity, to prevent the guest from being blocked. A glucose subunit was removed for visualization purposes. The specific bCD, HbCD, and guest anchor atoms selected are in Table S1 and the force constants in Table S2.

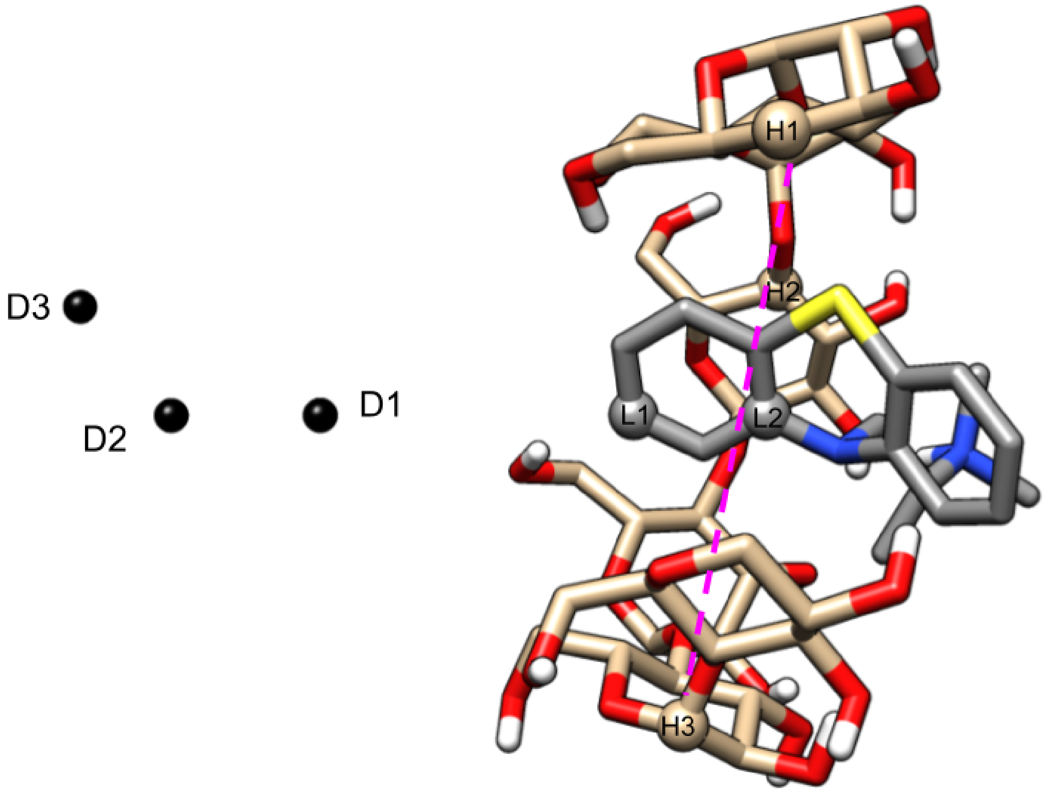

Figure S9 APR wall restraints setup for SAMPL9 cyclodextrin dataset. Following the schematic in figure S5, shown is the bCD-PMT complex with the wall restraints used for APR binding free energy calculations for this dataset. A glucose subunit was removed for visualization purposes. The magenta dashed line represents the harmonic flat-bottom restraints imposed to keep the PMT guest (via the L1 anchor atom) in the bCD cavity. The oxygen atoms (O3, O33, O28, O23, O18, O13, and O8) of each glucopyranoside linker were used to define the wall restraints. The force constant and distance criteria used for wall restraints are in Table S2.

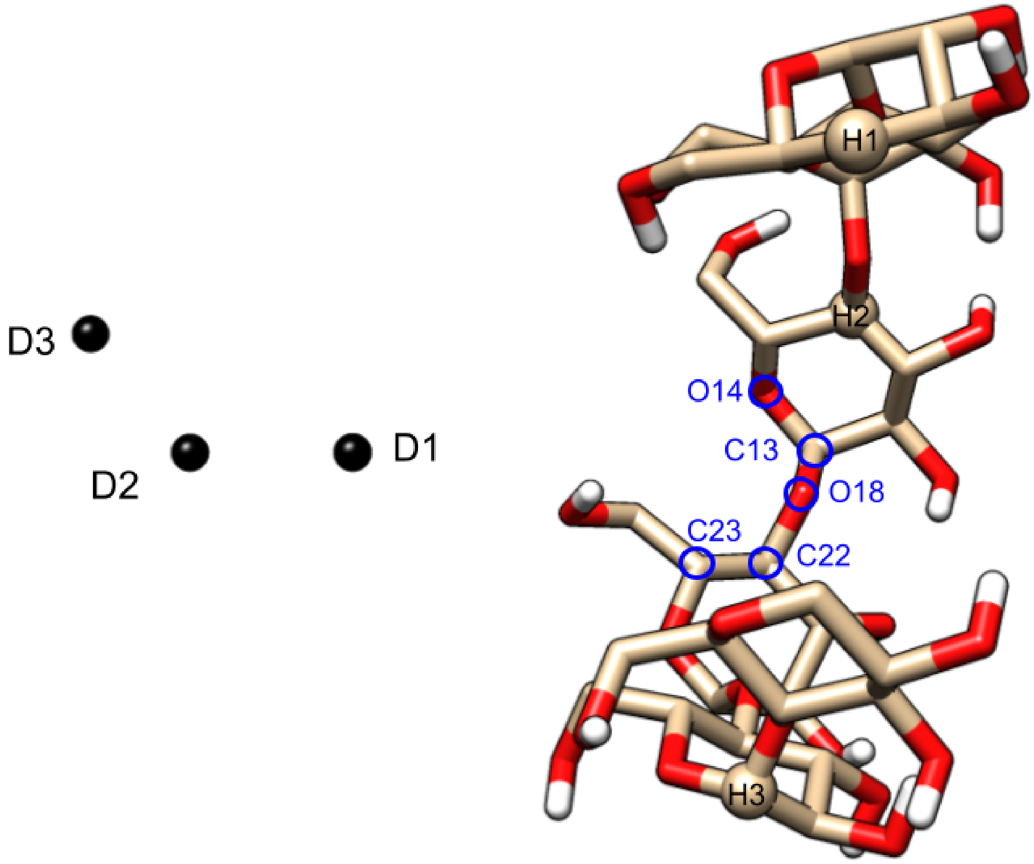

Figure S10 APR host dihedral restraint setup for SAMPL9 cyclodextrin dataset Shown is the bCD host with blue circles highlighting the atoms used to define a set of dihedral restraints on a glucopyranoside linker. One of the dihedrals is defined by atoms O14, C13, O18, and C22, and the second dihedral is defined by atoms C13, O18, C22, and C23. The PMT guest and a glucose subunit were removed for visualization purposes. The force constant and angles used are in Table S2.
